# Divergent regional responses of soil moisture-air temperature coupling under future climate scenarios

**DOI:** 10.1038/s41467-026-74040-w

**Published:** 2026-06-08

**Authors:** Daniel F. T. Hagan, Guojie Wang, Alan T. Kennedy-Asser, João L. Geirinhas, Kirsten L. Findell, Mingxing Li, Chenxia Zhu, Shijie Li, Diego G. Miralles

**Affiliations:** 1 Hydro-Climate Extremes Lab, Ghent University, Ghent, Belgium; 2 School of Remote Sensing & Geomatics Engineering, Nanjing University of Information Science & Technology, 210044 Nanjing, China; 3 School of Geographical Sciences, University of Bristol, Bristol, UK; 4 Cabot Institute for the Environment, University of Bristol, Bristol, UK; 5 Geophysical Fluid Dynamics Laboratory, National Oceanic and Atmospheric Administration, Princeton, NJ USA; 6 Key Laboratory of Regional Climate-Environment for Temperate East Asia, Institute of Atmospheric Physics, Chinese Academy of Sciences, Beijing, China; 7 The fifth Scientific Research Institute of Wuxi, Wuxi, China; 8 Department of Civil and Environmental Engineering, University of Florence, Firenze, Italy

**Keywords:** Climate change, Hydrology, Climate-change impacts

## Abstract

Climate models project that hotspot regions where soil moisture (SM) influences air temperature (T) will shift and strengthen, affecting droughts and heatwaves, yet the mechanisms driving these changes remain uncertain. Here, we use Coupled Model Intercomparison Project Phase 6 output and find divergent regional responses in SM–T coupling to different future emission scenarios during boreal summer. Under the low-end SSP1-2.6 scenario, SM–T coupling expands across all historical hotspots, while under the high-end SSP5-8.5 scenario, warming produces a latitudinal contrast, where SM–T coupling weakens and contracts at low-to-mid latitudes of the Northern Hemisphere but strengthens at higher latitudes. Coupling also strengthens in the humid tropics south of the equator, where disproportionate evaporation increases offset precipitation gains and depletes soil moisture. These shifts are driven by dynamic and thermodynamic changes modulating the atmospheric and land segments of SM–T coupling. Additionally, the influence of the poleward expansion of the Hadley cells, more pronounced under SSP5-8.5, further pushes SM control on surface energy partitioning northward. Together, these divergent responses reveal a nuanced, scenario-dependent future for SM–T coupling.

## Abstract (web summary)

Using climate-model projections, this study finds that regions where soil moisture controls summer heat will shift poleward under strong warming, raising the risk of compound drought– heatwave events at high latitudes and in the humid tropics.

## Introduction

Soil moisture and vegetation influence the state of the lower atmosphere through their control of the surface energy, carbon and water fluxes^1–4^. Decades of studies have demonstrated that land surface conditions may exacerbate the occurrence of climate extremes, such as heatwaves^5–7^, droughts^8–10^ and floods^11^. While changes in soil moisture (SM) modulate precipitation over multiple scales^3,12^, their effect on near surface temperature (T) is more immediate, localised and persistent over time^13,14^. The coupling between SM and T (hereafter referred to as SM–T coupling), defined as the strength of the dynamic control of SM on T anomalies, is modulated by evaporation, primarily by plant transpiration. Both model-based and observation-based studies have shown that SM–T coupling is the strongest during summer and over intermediate (or ‘transitional’) regions between wet and dry climates^15–18^. These regions exhibit large variability in SM, which often drops below the critical SM levels required to meet the atmospheric demand for water^19^. Although SM–T coupling has been widely studied using historical records^7,9,10,20,21^, uncertainties persist in its long-term evolution^20^.

Ongoing global warming is already affecting atmospheric circulation^22–25^, the hydrological cycle^26–28^ and land–atmospheric interactions^29–32^, increasing the overall likelihood of heatwaves and droughts^33^. These changes in the atmospheric circulation include the expansion and weakening of the Hadley cells (HC)^34,35^, implying the widening of the tropics and reduced intensity of tropical circulation, respectively. Consequently, these shifts not only lead to the poleward displacement of global pressure systems, precipitation and prevailing circulation patterns^36^, but also impact SM conditions^30^ and hydroclimatic regimes^28,35,37^, coexisting with the familiar ‘dry gets drier, wet gets wetter’ thermodynamic expectation under global warming^26,38–41^. In fact, the above-mentioned changes have already been detected in observations^24,34,35^, and climate models predict their further intensification in the future, particularly in the Northern Hemisphere^25^. Ultimately, the consequent changes in precipitation are expected to influence SM–T coupling and, in turn, projected increases in T, particularly during hot spells^20,42^.

While most previous studies have focused on the influence of SM on T at multiday time scales^43,44^, long-term SM changes can also partly explain prolonged regional T trends^18,45^. Such trends have been observed in some humid regions, such as Northern Europe and the tropical rainforest, where a shift towards water-limited conditions due to decreasing SM is expected^39,45,46^. Likewise, Central and Eastern Europe could become transitional climate zones resembling the present-day Mediterranean region^47^. However, uncertainties remain regarding the nature of long-term changes in SM–T coupling strength and spatial distribution—specifically, whether and where the influence of SM on T will weaken or intensify under different warming scenarios^48^. Furthermore, the changes in land and atmosphere conditions driving these potential shifts and the large-scale climate processes underlying them also remain unclear. These challenges arise from the complex and nonlinear nature of the SM–T coupling, which results from the combined contribution of two segments, known as the terrestrial and the atmospheric segments^43,49,50^. The first relates to the influence of SM on the surface energy balance, while the second addresses the ultimate impact of changes in the surface energy balance on air temperature. As such, changes in the coupling in response to different scenarios could arise from changes in either or both segments^43,49^.

Here, we aim to understand the SM–T coupling response to climate change by analysing shifts in coupling hotspots by the end of the 21st century. These shifts are particularly relevant for long-term climate predictions of extreme events, and related adaptation and mitigation strategies. An ensemble of 11 CMIP6 models, covering both historical (1981–2014) and future (2066–2100) periods under different future climate scenarios, is employed in this study. We focus on the tropics and NH (20^o^S to 90^o^N) during boreal summer (June–August). This period also aligns with the region and season in which SM–T coupling is more relevant and for which the most significant droughts and heatwaves have occurred over the past four decades^17,33,51^. Finally, we aim to unravel the drivers behind the long-term SM–T coupling changes and the potential role of thermodynamic and dynamical perturbations in these hydroclimatic shifts.

## Results

### Identifying hotspots of past and future boreal summer SM–T coupling

**Fig. 1:**
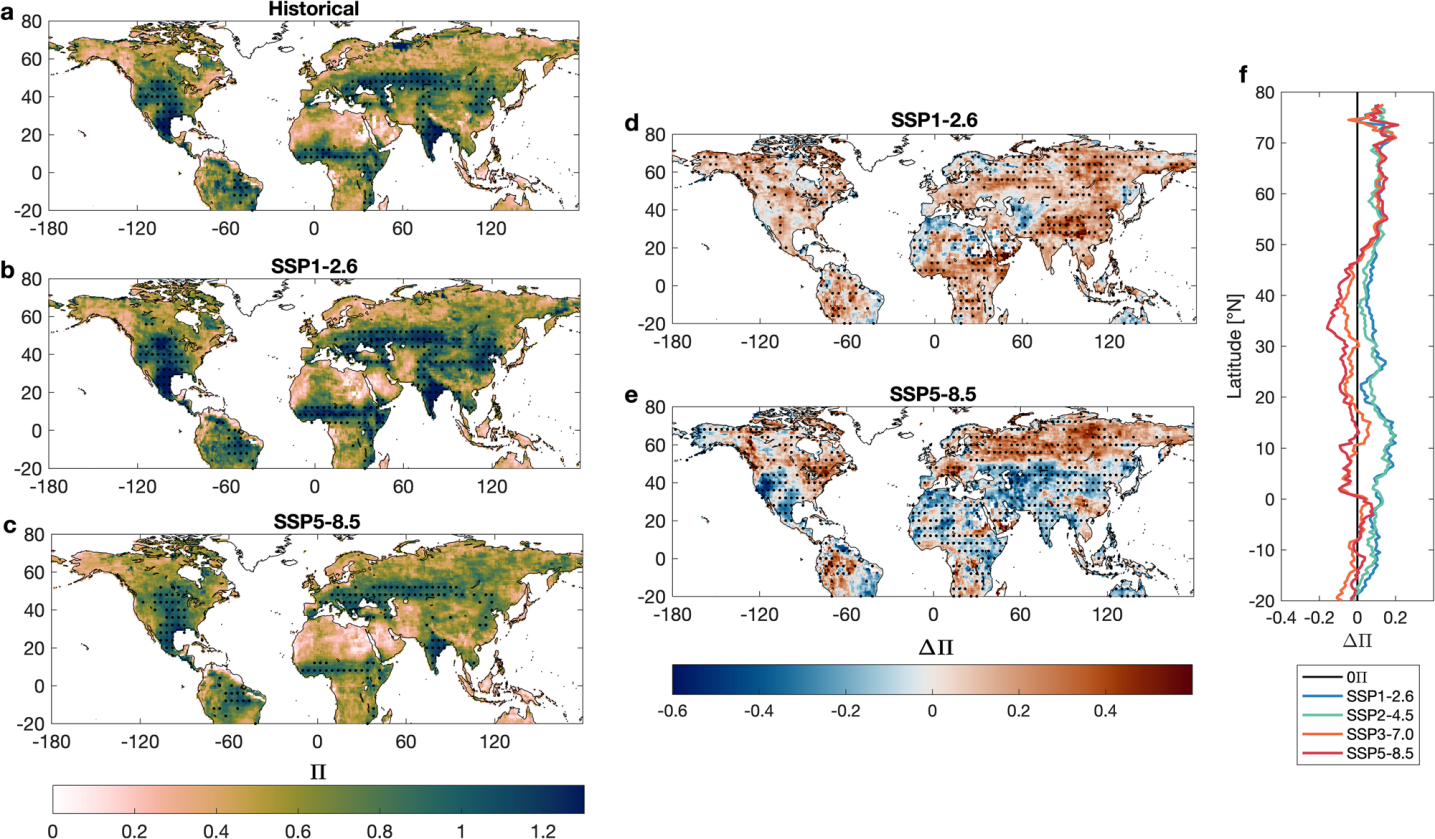
Soil moisture–temperature coupling and projected changes in boreal summer. **a** Soil moisture–temperature (SM–T) coupling ($\varPi$) based on the CMIP6 ensemble mean of the historical period (1981–2014). Same but for future projections (2066–2100) under **b** SSP1-2.6 and **c** SSP5-8.5. Dots indicate hotspot regions where at least 70% of the models agree on $\varPi$ > 0.75. Change in SM–T coupling (i.e. $\Delta \varPi$) during 2066–2100 relative to 1981–2014 under **d** SSP1-2.6 and **e** SSP5-8.5. Dots indicate regions where at least 70% of the models agree on the sign of the change in $\varPi$. **f** Mean latitudinal profile of $\varDelta \varPi$ for the future (SSP1-2.6, SSP2-4.5, SSP3-7.0 and SSP5-8.5) compared to the historical period. The correlations behind the $\varPi$ metric are computed at a 0.01 significance level with non-significant values shown in white.

To explore SM–T coupling, we employ the $\Pi$ diagnostic, which quantifies the influence of SM on T based on the differential skill of actual and potential evaporation to explain T anomalies^21^. This metric leverages the additional explanatory power of sensible heat flux on temperature relative to a hypothetical surface without moisture constraints, while isolating it from the influence of fluctuations in net radiation. As such, $\Pi$ captures the influence of long-term changes in water limitation as well as trends in cloudiness and advection^7,21^. $\Pi$ is a dimensionless quantity, with higher positive values indicating a stronger influence of SM on T. It indirectly incorporates the two segments of the coupling between SM and T: the SM constraint on the surface energy balance partitioning and the subsequent response of air temperature. The mean $\Pi$ during the historical period (1981–2014) is shown in Fig. 1a, computed using CMIP6 outputs and a Priestley and Taylor equation to calculate potential evaporation as per its original formulation^52^. The location of the main coupling hotspots—defined in Fig. 1a–c as the regions with $\Pi$ > 0.75—is consistent with results using observation-based data (Fig. S1a) and reanalysis products (Fig. S1b), revealing the general skill of CMIP6 climate models to represent this coupling. Coupling hotspots concentrate on transitional climates between arid and humid environments, in agreement with previous studies^16,21^. These locations include the Central Great Plains of North America, Northeast Brazil, the Sahelian region, India, southern Europe, Central Asia and central-east China (Fig. 1a). Some differences are found in the southern end of the Congo basin and Kazakhstan in ERA5 (Fig. S1b), possibly owing to unrealistic precipitation patterns over the Congo region in the reanalysis data^53^.

Future (2066–2100) projections show weakened SM–T coupling in low and mid-latitude regions (0°–40°N) under increasing emission scenarios (Fig. 1b, c). Under the low emission scenario (SSP1-2.6), the SM–T coupling becomes stronger and expands (Fig. 1b, Fig. S2a), although it remains concentrated in the same historical SM–T coupling hotspot regions. However, the coupling at those latitudes weakens and contracts under the high emission scenario (SSP5-8.5) (Fig. 1c, Fig. S2a), while intermediate scenarios fall within the envelope set by SSP1-2.6 and SSP5-8.5 (Fig. S2b, c). The European and Central Asian belts, particularly the Mediterranean basin, are expected to either predominantly remain hotspots or strengthen under all SSPs^6^. Under SSP5-8.5, significantly reduced SM–T coupling is found in the Sahel, Eastern China, parts of India and North America (Fig. 1c). These SM–T coupling patterns (Fig. 1a–c) show high agreement among CMIP6 models (Fig. S3).

All projection scenarios reveal a poleward migration of SM–T coupling regions and the emergence of coupling over some intertropical humid regions, such as western Amazonia and southeast China; Fig. 1d, e (and Fig. S2d, e) illustrate the SM–T coupling differences between the future (2066–2100) and historical period (1981–2014). This zonal shift in SM–T coupling aligns with amplified warming across midlatitude and tropical continental interiors^28,38,54^. Trends differ between emission scenarios in intertropical regions: lower emissions lead to increased coupling, while higher emissions lead to decreased coupling. From Fig. 1d, e, we find weakening hotspots in current climate transitional regimes, mainly in the higher emission scenarios, together with increases in SM–T coupling at mid-to-high latitudes. Changes in high latitudes suggest a poleward emergence of SM–T coupling under global warming regardless of the emission scenario, with the strongest emergence found in SSP5-8.5. Additionally, we observe westward movements of the emerging couplings, notably in the high latitudes, the Amazonia and even in southeast China^54^. These tend to characterise an altogether northwestern movement of the emerging regions, though the poleward movement appears more prominent. Overall, stronger SM–T coupling is expected in humid zones under SSP5-8.5, potentially driven by warming-induced shifts in SM climatology^30^.

Overall, the latitudinal variations of changes in $\Pi$, shown in Fig. 1f, reveal a regional difference in the SM–T coupling across different emission scenarios. Under SSP5-8.5, the coupling is weaker in low latitudes and intertropical regions, but stronger over mid-to-high latitudes, with the opposite patterns found under low emissions.

### SM–T coupling dependence on land and atmosphere segments

As discussed above, SM–T coupling is inherently nonlinear and can be decomposed into a land segment and an atmospheric segment^18,43,49,55^. Both segments are expected to adjust under global warming as thermodynamic constraints (soil water availability and radiative energy) and dynamical conditions (boundary-layer and circulation influences) evolve. Here, we use this decomposition as a diagnostic framework to interpret the hotspot changes in $\Pi$ shown in Fig. 1, rather than to provide a direct reconstruction of Δ$\Pi$ from the two sensitivities. Specifically, we characterise the land segment by the sensitivity of evaporative fraction (EF; latent heat divided by net radiation) to SM, δEF/δSM and the atmospheric segment by the sensitivity of near-surface air temperature to EF variability, δT/δEF (plotted with reversed sign for visual consistency)^43,49^. In physical terms, strong SM–T coupling is most likely where (i) EF responds appreciably to SM anomalies and (ii) temperature responds appreciably to EF fluctuations, subject to constraints imposed by soil water supply and available energy^12^.

**Fig. 2:**
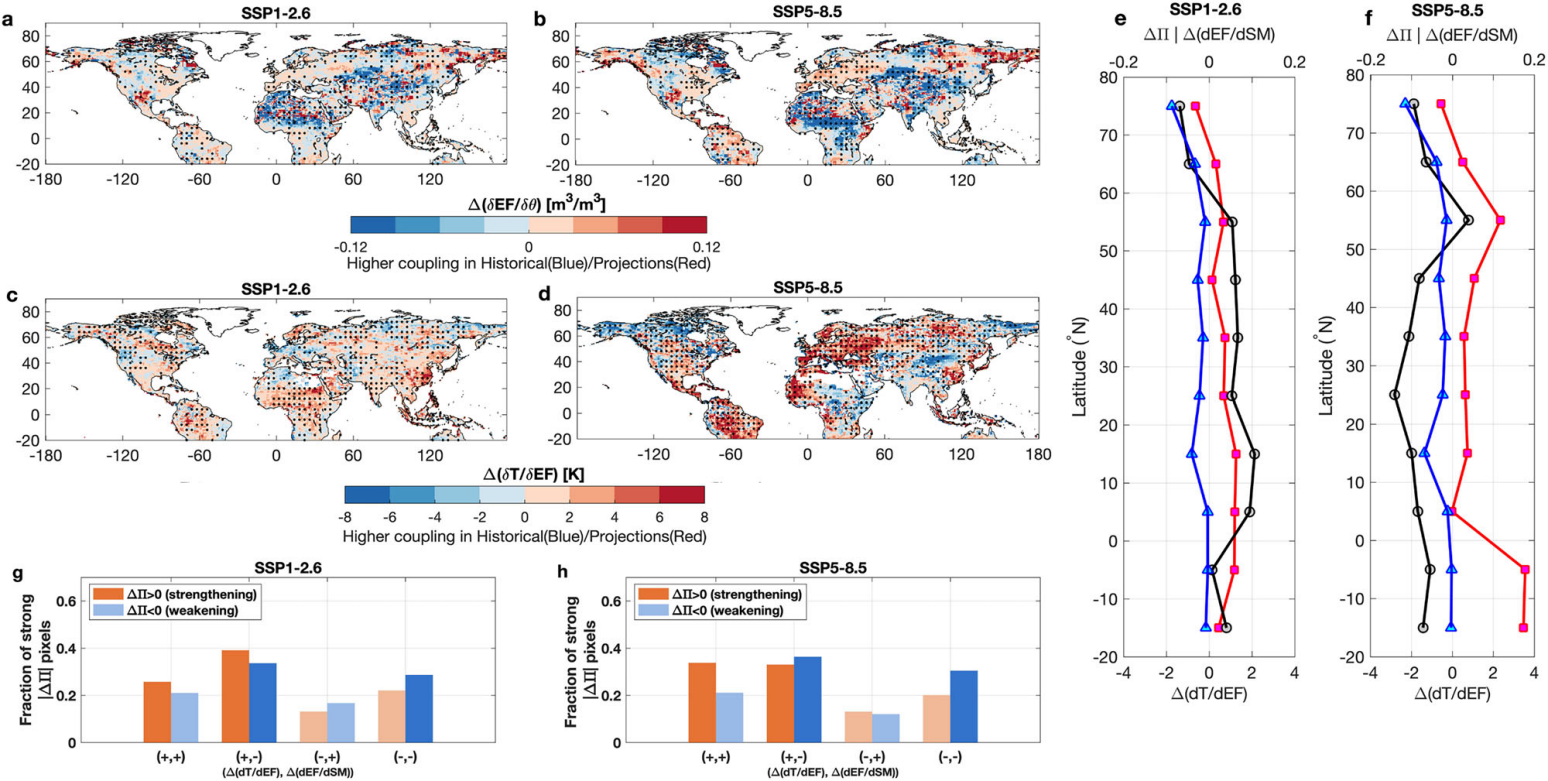
Future changes in the land and atmosphere segments of soil moisture–temperature coupling. **a** Changes in the land segments of the coupling, expressed as the evaporative fraction (EF) sensitivity to soil moisture (SM) changes ($\Delta$(δEF/δSM), m^3^m^−3^), for the period 2066–2100 relative to 1981–2014 under SSP1-2.6. **b** same as (**a**) but for SSP5-8.5. **c** Changes in the atmospheric segments of the coupling, expressed as temperature (T) sensitivity to EF changes ($\Delta$(δT/δEF), K), for the period 2066–2100 relative to 1981–2014 under SSP1-2.6. **d** same as (**c**), but for SSP5-8.5. –(δT/δEF) is used here for visual consistency. Dots indicate regions where at least 8 of the models agree on the sign of the change in the segments. All the segment computations are done at a 0.01 significance level based on a *z*-test. Latitude-binned median changes (10° bins) in Δ$\Pi$, $\Delta$(δEF/δSM) and $\Delta$(δT/δEF), computed within the top 60% of historical |$\Pi$| for **e** SSP1-2.6 and **f** SSP5-8.5. Quadrant analysis of co-occurring segment changes for the strongest 60% of |Δ$\Pi$| pixels for **g** SSP1-2.6 and **h** SSP5-8.5. Bars show the fraction of strengthening (Δ$\Pi$ > 0; orange line) and weakening (Δ$\Pi$ < 0; blue line) pixels falling into each combination of Δ(δT/δEF) and Δ(δEF/δSM) signs. Darker coloured bars highlight the dominant co-occurrence patterns that most strongly characterise $\Pi$ strengthening and weakening.

Figure 2a–d present the projected changes in the two segment sensitivities under SSP1-2.6 and SSP5-8.5, while Fig. 2e–h summarise their zonal behaviour and co-occurrence with Δ$\Pi$. Here, we intend to diagnose the dominant physical sensitivities underlying coupling change, rather than to reproduce the spatial pattern of Δ$\Pi$ itself. The maps reveal that global warming modifies the land and atmospheric segments in distinct and regionally structured ways (Fig. 2a–d). Under SSP1-2.6 (Fig. 2a, c), changes in both segments are generally weak and spatially heterogeneous, indicating only modest adjustments in the sensitivities governing SM–T interactions. The atmospheric segment shows limited coherent change, and variations in the land segment are largely mixed in sign, consistent with the muted and spatially patchy Δ$\Pi$ responses in this scenario. Under SSP5-8.5 (Fig. 2b, d), however, a clearer pattern emerges. The atmospheric segment exhibits widespread strengthening, particularly across the northern mid-latitudes (30–60°N), where Δ(δT/δEF) becomes more positive. This indicates that, in these regions, a given perturbation in EF would elicit a larger temperature response in the future climate, implying that temperature becomes more tightly controlled by local surface fluxes and less moderated by non-local atmospheric processes. In contrast, several semi-arid and subtropical regions display reductions in δEF/δSM, implying that SM anomalies become less effective at modulating EF than in the historical period—an indication of weakening land surface control. Taken together, these patterns suggest two contrasting tendencies: in regions where coupling hotspots emerge or intensify, the dominant signature is enhanced atmospheric sensitivity to EF variability, whereas in regions where hotspots weaken, the prevailing signal is a decline in the efficiency with which SM anomalies translate into evaporative changes, reflecting a lower coupling of EF from SM.

To further clarify how the two segments relate to changes in $\Pi$, we focused on grid cells within the top 60% of historical $|\Pi |$, representing the principal historical SM–T transition belt. Within this subset, we computed median latitude-binned changes in Δ(δEF/δSM), Δ(δT/δEF) and Δ$\Pi$ (Fig. 2e, f). This aggregation indicates a large-scale perspective on how the dominant segment sensitivities evolve across latitude, while reducing local noise and model-specific variability. Under SSP1-2.6 (Fig. 2e), all three fields display only weak and irregular latitudinal structure, consistent with the generally modest changes in $\Pi$ shown in Fig. 1b. The limited coherence of both Δ(δT/δEF) and Δ(δEF/δSM) reflects the relatively small thermodynamic and dynamical perturbations under weak warming, consistent with coupling responses remaining spatially heterogeneous. Under SSP5-8.5 (Fig. 2f), a much clearer signal emerges. Mid- and high-latitude bands exhibit pronounced positive Δ(δT/δEF), indicating a systematic increase in atmospheric sensitivity to evaporative variability. In contrast, Δ(δEF/δSM) remains comparatively moderate and mixed in sign, with only localised reductions in land segment sensitivity. The latitude-binned Δ$\Pi$ closely tracks the pattern of Δ(δT/δEF), consistent with the large-scale amplification and poleward expansion of coupling hotspots being primarily associated with enhanced atmospheric responsiveness, rather than with uniform changes in land surface control. At the same time, deviations between Δ$\Pi$ and Δ(δT/δEF) in specific latitude bands highlight that segment changes alone do not uniquely determine Δ$\Pi$, consistent with the nonlinear and regime-dependent nature of the $\Pi$ metric.

Finally, the co-variation between segment changes and $\Pi$ strengthening (Δ$\Pi$ > 0) or weakening (Δ$\Pi$ < 0) is examined using a contingency-style analysis applied to the top 60% of |Δ$\Pi$ | pixels, representing locations with the strongest coupling changes (Fig. 2g–h). For each scenario, pixels are classified according to the signs of Δ(δT/δEF) and Δ(δEF/δSM), yielding four possible sign combinations: (+,+), (+,−), (−,+) and (−,−). Within the strengthening and weakening subsets, we compute the fraction of pixels falling into each quadrant. Under SSP1-2.6 (Fig. 2g), the four sign combinations occur in broadly comparable proportions for both $\Pi$ strengthening and weakening. Although strengthening pixels exhibit a modest preference for Δ(δT/δEF) > 0, Δ(δEF/δSM) is nearly evenly split between positive and negative values. This lack of a dominant quadrant indicates that, under weak warming, no single segment consistently governs coupling changes, consistent with the muted and spatially heterogeneous Δ$\Pi$ patterns. Under SSP5-8.5 (Fig. 2h), a markedly different structure emerges. Among $\Pi$ strengthening pixels, 70.4% coincide with Δ(δT/δEF) > 0, indicating that coupling intensification preferentially occurs where the atmosphere becomes more sensitive to evaporative variability. A substantial subset of these pixels also exhibits Δ(δEF/δSM) > 0, suggesting that in some regions land and atmospheric segments act in concert. In contrast, $\Pi$ weakening pixels show a strong preference for Δ(δEF/δSM) < 0 (67.1%), demonstrating that a reduction in the efficiency with which SM anomalies modulate EF is the dominant signature of hotspot weakening. Importantly, these associations emerge at the aggregate level despite imperfect grid-scale correspondence, highlighting that $\Pi$ responds nonlinearly to segment changes but nonetheless exhibits clear, scenario-dependent tendencies.

**Fig. 3:**
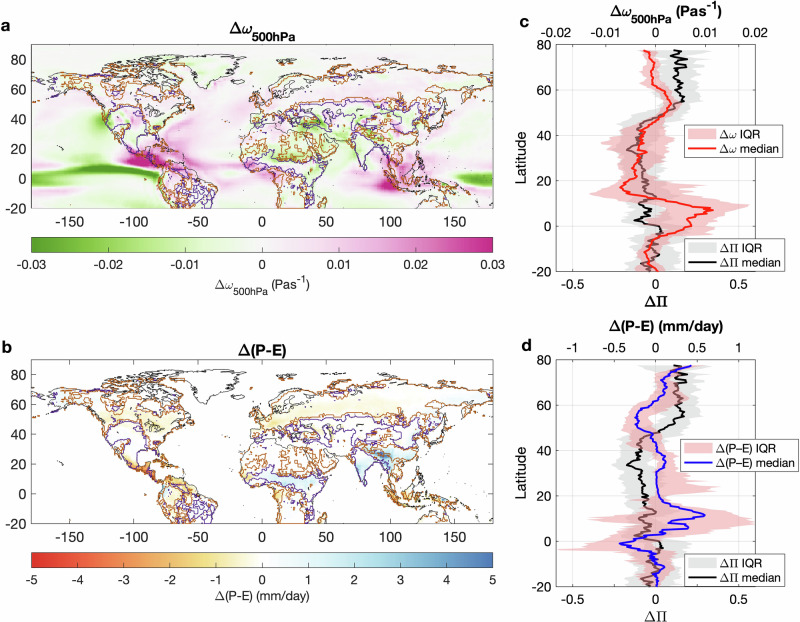
Mechanisms behind the boreal summer soil moisture–temperature coupling changes under high emissions. Change in **a** vertical wind velocity at the 500 hPa level (shading) for boreal summer during 2066–2100 (SSP5-8.5), relative to the historical baseline period 1981–2014 ($\Delta {{\rm{\omega }}}$_500_). Green means increased ascending velocity, while magenta means increased descending velocity. **b** same as (**a**) but for change in precipitation minus evaporation ($\Delta$(P–E)), where blue(red) shades mean increased(decreased) $\Delta$(P–E). The contour overlays represent changes in SSP5-8.5 $\Pi$, where brown represents increases and violet represents regions of decreases. Mean latitudinal profile of future change in **c** vertical winds ($\Delta {{\rm{\omega }}}$_500_: red) and $\Delta \Pi$(black) over terrestrial regions. **d** same as (**c**) but for $\Delta$(P–E) in blue. The inter-model uncertainties are represented by the interquartile ranges and medians, depicted by shades and solid lines, respectively.

Overall, the contingency analysis (Fig. 2g, h) reveals two complementary pathways of SM–T coupling change. Hotspot strengthening occurs predominantly where the atmospheric segment intensifies, such that temperature becomes more responsive to evaporative variability. In contrast, hotspot weakening arises mainly from a decline in land-surface control, where SM anomalies translate less effectively into evaporative changes. These contrasting behaviours explain why coupling responses diverge between scenarios: under weak warming, segment changes are modest and spatially mixed, yielding limited and heterogeneous Δ$\Pi$, whereas under strong warming, coherent increases in atmospheric sensitivity drive the amplification and poleward migration of coupling hotspots. Figure 2 therefore demonstrates that the two segments play distinct and scenario-dependent roles. Under SSP5-8.5, the emergence and expansion of hotspots are primarily associated with increases in δT/δEF, while the decay of historically strong hotspots is more often linked to reductions in δEF/δSM. At the same time, the imperfect grid-scale correspondence between segment changes and Δ$\Pi$ underscores that coupling evolution also depends on broader dynamical adjustments. These dynamical controls, which modulate how segment sensitivities translate into coupling outcomes, are examined next in Fig. 3. Beyond the aggregate co-occurrence statistics, further examination over four canonical regions (Sahel, Mediterranean, Northern Europe and Western Amazonia) confirm via conditional probability analysis that segment signs shift the odds of Δ$\Pi$ direction by factors of 2–3 under SSP5-8.5, with the land segment specifically requiring strong warming to engage (Fig. S4).

### Large-scale dynamics driving the future SM–T coupling deviation patterns

Changes in SM–T coupling—whether expressed through the land segment (δEF/δSM), the atmospheric segment (δT/δEF), or their combined influence—ultimately arise from underlying thermodynamic and dynamical forcing. Long-term shifts in coupling hotspots therefore reflect evolving patterns of temperature, precipitation and atmospheric circulation driven by greenhouse-gas forcing and internal variability. While the segment analysis in Fig. 2 identifies which sensitivities tend to strengthen or weaken, it does not explain why those sensitivities change. Understanding the physical drivers of Δ$\Pi$ thus requires examining the broader climate dynamics that modulate surface energy and moisture budgets. In principle, radiative forcing and associated feedbacks can reorganise large-scale atmospheric circulation—for example through enhanced poleward heat transport, shifts in mid-latitude jet streams, or expansion of the Hadley circulation^26,56–59^. Such changes alter vertical motion, moisture convergence and the balance between precipitation and evaporation, all of which influence the effectiveness with which SM anomalies affect temperature. To investigate these mechanisms, we focus on two key indicators of dynamical adjustment: changes in mid-tropospheric vertical velocity at 500 hPa ($\Delta {{\rm{\omega }}}$_500_) and changes in the surface water balance (Δ(P–E)). These diagnostics are analysed primarily under the high-emission SSP5-8.5 scenario, where Δ$\Pi$ exhibits the most pronounced and spatially coherent responses (Fig. 1e).

Figure 3a shows future changes in $\Delta {{\rm{\omega }}}$_500_ relative to the historical baseline period (green-magenta shadings), where green represents increased ascent and magenta indicates enhanced descent. Regions largely maintain their respective vertical motion directions in the future scenarios, although with varying magnitudes (Fig. S5). Northern United States, Central and Southern Europe and the Amazon basin exhibit positive $\Delta {{\rm{\omega }}}$_500_, indicating stronger subsidence (and potentially more anticyclonic conditions) in the future. Similarly, parts of Southern Sahel, particularly in the east, show weaker ascent, which would likely reduce convective rainfall. However, SSP5-8.5 projections also suggest stronger low-level tropospheric winds (Fig. S6) facilitating enhanced moisture transport from the Gulf of Guinea to the Sahel, thereby increasing the likelihood of precipitation in the region. Conversely, the northern Sahel, India and the intertropical United States display stronger rising air motion, which could enhance convective precipitation and potentially shift these regions towards more humid conditions. Notably, there are indications of a northward displacement of the Sahelian rainfall belt, possibly reflecting the expanding Hadley circulation (Fig. 3a).

Overlaying $\Delta {{\rm{\omega }}}$_500_ with the $\Delta \Pi$(Fig. 1d–f) reveals that regions of decreased coupling strength (violet contours) align with regions of increased ascent (green shades) where $\Delta$(P–E) (Fig. 3b) increases under SSP5-8.5. These include intertropical historical hotspots such as the Sahel and India, as well as temperate western United States and the central-eastern Huabei region (China). These historical hotspots weaken in the future due to increased P–E, leading to wetter surface conditions and a consequent shift towards an energy-limited evaporation regime, thereby reducing the control of SM on the energy balance partitioning. Conversely, regions experiencing increased coupling strength correspond to intensified subsidence areas. The increased subsidence is likely a key factor behind rainfall decreases and drier surface conditions. Over time, these regions transition from historically humid conditions towards semi-humid conditions enhancing the sensitivity of the energy balance partitioning to SM conditions and thereby strengthening SM–T coupling. The estimated future decreases in the near surface relative humidity over these areas (negative values of $\Delta$SurfRH in Fig. S7b) reflect these changing patterns. As a summary, the latitudinal variations in Fig. 3c, d highlight distinct changes in atmospheric circulation and P–E, demonstrating their influence on coupling shifts and particularly on a poleward emergence of the SM–T coupling potentially linked to the expansion of the HC circulation.

## Discussion

Under future emission scenarios, regional responses are distinct, leading to different trajectories of the SM–T coupling. Under low-emission pathways, present-day coupling patterns are largely preserved and even amplified, whereas they diminish under high-emission pathways. Specifically, global warming drives a latitudinal contrast marked by SM–T coupling decline at historical low latitudes hotspots and emerging hotspots towards higher latitudes. In the 21st century, these emerging hotspots are most pronounced under strong-warming scenarios across mid- to high-latitude regions of North America and northern Europe (Fig. 1, Fig. S8). Additionally, certain regions located between the declining and emerging zones—such as parts of the Mediterranean—are projected to remain persistent SM–T coupling hotspots under high-emission scenarios (Fig. S8). By contrast, under low-emission scenarios, intertropical transitional zones are estimated to witness an intensification and expansion of the current SM–T coupling conditions. This emission-scenario dependence produces regional differences in SM–T feedbacks. Furthermore, the high-emission pathway also yields nuanced, region-specific responses that further illustrate these divergent changes (Fig. S8).

**Fig. 4:**
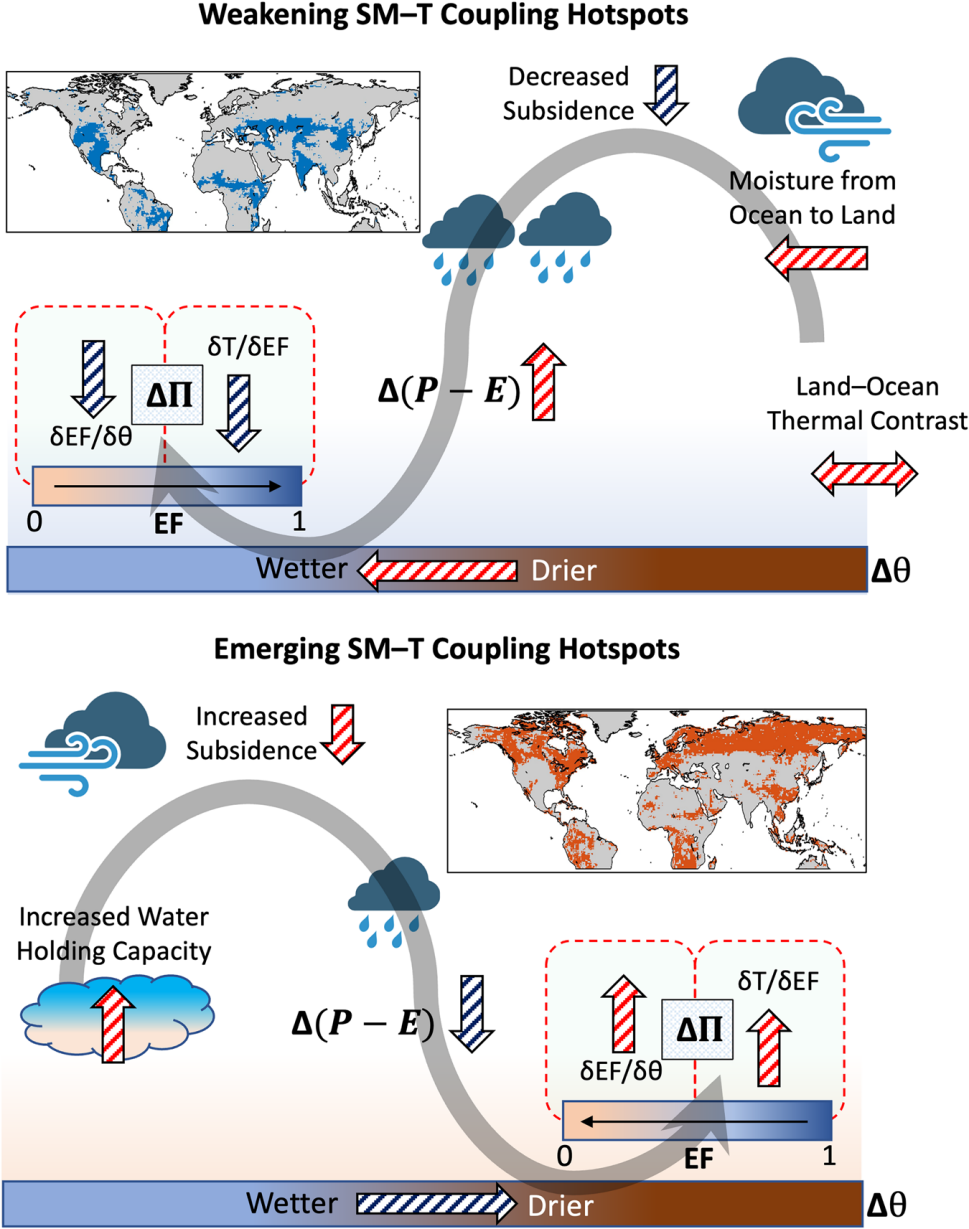
Regional divergence of soil moisture–temperature coupling under global warming. Warming in the future (under SSP5-8.5) yields distinct soil moisture–temperature (SM–T) coupling responses. In historical hotspot regions with increased inland moisture transport and reduced subsidence in the future (top figure), precipitation is expected to increase, leading to wetter soil conditions. This reduces the sensitivity of evaporative fraction to soil moisture (δEF/δSM) resulting in a weakening of SM–T coupling and a steering towards energy-limited conditions. In contrast, regions experiencing a future enhancement of land heating and atmospheric subsidence are expected to witness a heightened evaporation, drier soils and strengthened land–atmosphere feedbacks, reinforcing emergent SM–T coupling (bottom figure).

Figure 4 illustrates the potential physical processes governing future declining and emerging regions of SM–T coupling. Under high emissions (SSP5-8.5), regions transitioning from stronger to weaker SM–T coupling (top panel) such as the Sahel and India, exhibit shifts in large-scale atmospheric circulation, which might draw in more moisture from the ocean and enhanced vertical ascent (decreased subsidence). This will lead to an increased P–E and persistently higher SM. The resulting wetter soil surface weakens the sensitivity of evaporation to SM changes, in part owing to increased P–E (Fig. 3a) and relative humidity (Fig. S7). Conversely, most regions with emergent SM–T coupling undergo increased subsidence, driving the descent of warm air, which elevates surface temperatures. Consequently, this results in increased atmospheric demand, enhanced evaporation (decreased P–E) and, ultimately, decreases in SM. Although historically energy-limited, these regions are expected to progressively stir towards water-limited conditions due to increased T dependence on EF. This strengthens land–atmosphere feedbacks and reinforces SM control over T. Together, these opposing patterns demonstrate widespread hydroclimatic shifts in evaporative control, leading to regionally divergent patterns of the SM–T coupling regimes across the emission scenarios. In addition, the apparent extension of tropical conditions^25,60^—consistent with a poleward expansion of the HC^24,34,60^—provides a coherent explanation for the poleward migration of SM–T coupling, as this enhances SM variability in these regions such as Northern Europe and the Amazon Basin.

Here, we demonstrate that the future distribution of SM–T coupling hotspots exhibits a clear scenario-dependent bifurcation, driven by the contrasting warming and drying trajectories across climate projections. Under the high-end emissions scenario, this leads to divergent regional responses, with pronounced latitudinal contrasts and zonal shifts in the spatial pattern of SM–T coupling hotspots. An important implication of these findings is a higher likelihood of heatwaves and compound dry–hot events modulated by SM–T coupling changes in the emerging coupling regions. These appear to be found in humid high latitude and tropical humid areas via a disruptive partitioning of the surface heat fluxes^21^. Under these conditions, temperature increases are expected to be amplified by land dry-outs, which constrain evaporation and increase sensible heat flux, exacerbating heat extremes^3,61^. Additionally, understanding SM–T coupling changes is crucial in formulating adaptation and mitigation strategies in agriculture to ensure food security and water availability in hotspot regions. To adequately quantify this, human activities such as irrigation and land use and cover changes may also have to be considered to fully capture changes in the coupling, especially in its terrestrial segment.

## Methods

### CMIP6 projections

In this study, we use eleven climate model outputs from the CMIP6 project (see Supplementary Table S1), covering historical (1981–2014) and projection (2066–2100) periods. The future period (2066–2100) is chosen because $\varPi$ is stable during this timeframe in the scenarios after a period of transitioning. We use seasonal mean net radiation, near-surface mean air temperature and land surface evaporation to compute the $\varPi$ coupling diagnostic of SM–T coupling for boreal summer (June, July and August) at the annual scale. We also use latent and sensible heat fluxes to compute the evaporative fraction, and total SM content. Four climate change pathways, SSP1-2.6, SSP2-4.5, SSP3-7.0 and SSP5-8.5 from the CMIP6 projections^62^ are considered. SSP1-2.6 and SSP5-8.5 describe the low- and high-end emissions, respectively. The former represents a future world moving towards sustainable practices, while the latter represents a future world with an energy-intensive, fossil-based economy. SSP2-4.5 represents a world where projection trends continue the historical patterns, and SSP3-7.0 describes a middle-road emission scenario. For model consistencies, models with all the variables at the first (or second, if the first is absent, i.e. r1i1p1f1 or r1i1p1f2) realisation for all four scenarios are selected (Table S1). All the computations are conducted at the original spatial resolution of individual CMIP6 models, then resampled to a common 0.5° x 0.5° spatial resolution using bilinear interpolation to compute the ensemble means.

### Observation and reanalysis products

We use two sets of independent datasets to validate our results from the CMIP6 coupling computations. Monthly temperature observations are obtained from the Climate Research Unit (CRU) archives^63^, which have a spatial resolution of 0.5° × 0.5°. Actual and potential evaporation are obtained from the Global Land Evaporation Amsterdam Model (GLEAM3.6a), which considers a combination of satellite and reanalysis data^64,65^. These datasets are then resampled to a 0.5° × 0.5° spatial resolution to match the CRU and CMIP6 data. Global atmospheric reanalysis products from the European Center for Medium-range Weather Forecast (ERA5) are also used to compute the coupling diagnostic; these high-quality, high-resolution products have been validated consistently^66^. Here, we use monthly datasets (June, July and August) from 1981 to 2014 for the historical computations. Parameter data preprocessing steps follow the steps used for the CMIP6 datasets.

### The SM–T coupling diagnostic$(\varPi )$

The $\varPi$ coupling diagnostic is used to quantify the impact of changes in SM on the overlaying near-surface mean air temperature (*T*). It uses the difference between the actual (*E*) and potential evaporation (${E}_{p}$) to explain changes in *T*. Miralles, et al.^21^ defined this metric as:

1 $$\varPi=\rho (H,T)-\rho ({H}_{p},T)$$

where $\rho$ is the Pearson correlation coefficient, $H={R}_{n}-\lambda E$, and ${H}_{p}={R}_{n}-\lambda {E}_{p}$. $\lambda$ represents the latent heat of vaporisation and ${R}_{n}$ represents net radiation. The metric is a dimensionless quantity ranging between minimum and maximum values of –2 and 2, respectively. Higher positive values indicate stronger SM–T coupling, where SM is the limiting factor for the coupling. Negative values are infrequent and generally masked out. Here, we used ${R}_{n},\ \lambda{E}_{p}\ {and\ T}$ outputs from both the climate models and observational data (i.e. GLEAM, CRU, ERA5).

Potential evaporation, $\lambda {E}_{p},$ is estimated based on the Priestley and Taylor (PT) formulation^52^, following Miralles, et al.^21^. The PT formulation here uses ${R}_{n}\ {and\ T}$ as inputs such that

2 $$\lambda {E}_{p}=\alpha \left(\frac{\varDelta }{\varDelta+\gamma }\right){R}_{n}-G$$

where $\alpha$ parameterises the resistance to evaporation (dimensionless), $\varDelta$ is the gradient of the function $\delta {q}_{s}$/$\delta T$ (where $\delta {q}_{s}$ is the saturated vapour pressure) in kPa °C^-1^, $\gamma$ is the psychrometric constant in kPa °C^−1^ and *G* is the ground heat flux (here assumed to be 0.2 ⋅ *R*_*n*_). $\varDelta$ can be calculated as follows:

3 $$\varDelta=\frac{4098{q}_{s}}{{(237.3+T)}^{2}}$$

And ${q}_{s}$ is calculated (in kPa) as:

4 $${q}_{s}=0.6108\,exp\left(\frac{17.27\,T}{237.3}\right)$$

The $\varPi$ coupling diagnostic is a process-based metric that has been thoroughly validated using model-based and observational datasets^16,21^. Although it provides a robust understanding of SM–T coupling, some limitations persist. $\lambda {E}_{p}$ is computed based on a number of assumptions (Eqs. 2–4) and, as a result, several measures are taken to constrain the computation of $\varPi$. ${R}_{n}$ and $\lambda {E}_{p}$ are constrained to be ≥ ${0}$. Since $\lambda E$ cannot exceed $\lambda {E}_{p}$ theoretically, $\lambda E$ is set to $\lambda {E}_{p}$ in cases the Priestley and Taylor equation yields values smaller than $\lambda E$ (even though in such cases no coupling will be predicted by the $\varPi$ metric). Statistical significance is computed for the correlation values at 0.01% based on the Student’s *t* test.

While we consistently inspect agreement in the CMIP6 multi-model analyses (at least, 80% of the models agree), CMIP6 ensembles are known to over-represent SM constraints on evaporation and overestimate, which has implications for the SM–T coupling computations over mid-latitudes^37^. While our historical evaluation against observations lends confidence, our findings should be interpreted as model-conditioned tendencies rather than absolute magnitudes, acknowledging that consensus can mask the structural biases shared across models. Furthermore, due to an assumption as the constant value of $\alpha$ within the PT formulation, the impact of climate change in the future SM–T coupling might be biased. Even so, the consistency of future SM–T estimates obtained using the $\varPi$ diagnostic lends confidence in our results. Additionally, SM–T coupling patterns in previous studies agree with the patterns we find here in $\varPi$^16,43,49^.

### The SM–T partitioning

We follow the approach from previous studies, which suggested that the sensitivity of near-surface air temperature (*T*) to SM ($\theta$) changes could be divided into two segments^43,49^ as shown in Eq. (5), which quantifies potential pathways of SM–T coupling:

5 $$\frac{\delta T}{\delta \theta }=\frac{\delta T}{\delta {EF}}\frac{\delta {EF}}{\delta \theta }$$

where $\delta {EF}/\delta \theta$ relates to the sensitivity of changes in the evaporative fraction ($\delta$EF) to changes in SM, and $\delta T/\delta {EF}$ relates to the influence of EF changes on T changes. When both segments are high, the influence of SM on T is the largest^43^, however, this combination is also shaped by physical limitations in water and energy availability which may vary across climate models. Furthermore, the use of EF limits a biased influence of Rn in the interaction, which may contribute to differences in the overall patterns with $\varPi$. These partial derivatives are computed based on statistically significant slopes using a *z*-test. The first segment is in Kelvin (K), while the second segment can be assumed to be unitless. Unlike correlation-based metrics which may respond linearly to background warming, obscuring regime-dependent nonlinearities that dominate land–atmosphere interactions, this approach explicitly computes the sensitivity of EF to SM changes across dry, transitional and wet regimes^18,43,48^. Thus, it provides a more physically nuanced view of the terrestrial segment of the SM–T coupling under climate change. Nonetheless, differences between the changes obtained from this partitioning and those in $\Delta \varPi$ are expected, as the former is intrinsically more responsive to changes in SM regime occupancy, while $\varPi$ reflects shifts in the actual–versus–potential evaporation contrast.

## Supplementary information

Supplementary Information

Transparent Peer Review file

## Data Availability

All data needed to evaluate the conclusions in the paper are present in the paper and/or the Supplementary Materials. Additional figures that may help interpret the results are available in the Supplementary Materials. In addition, CMIP6 climate model data are publicly available from the ESGF. Data to reproduce the figures of this paper are available on zenodo at 10.5281/zenodo.20122979.

## Code availability

The codes to compute the coupling diagnostics and datasets to plot the figures in the paper are available on Zenodo at 10.5281/zenodo.20122979.
