# Supplementary material for: Divergent regional responses of soil moisture-air temperature coupling under future climate scenarios
Source: Nat Commun. 2026 Jun 8;17:7311. doi: 10.1038/s41467-026-74040-w (PMC13402682; doi:10.1038/s41467-026-74040-w)
Supplement: Supplementary file 1 — Supplementary Information [file 41467_2026_74040_MOESM1_ESM.pdf]

Supplementary Information for

Divergent regional responses of soil moisture-air temperature coupling under future climate scenarios

Daniel F.T. Hagan[1] (daniel.hagan@ugent.be), Guojie Wang[2], Alan T. Kennedy-Asser[3,4], João L. Geirinhas[1], Kirsten L. Findell[5], Mingxing Li[6], Chenxia Zhu[2], Shijie Li[7], Diego G. Miralles[1]

[1]Hydro-Climate Extremes Lab, Ghent University, Ghent, Belgium.

[2]Collaborative Innovation Center on Forecast and Evaluation of Meteorological Disasters, Nanjing University of Information Science & Technology, Nanjing 210044, People's Republic of China.

[3]School of Geographical Sciences, University of Bristol, Bristol, United Kingdom.

[4]Cabot Institute for the Environment, University of Bristol, Bristol, United Kingdom.

[5]Geophysical Fluid Dynamics Laboratory, National Oceanic and Atmospheric Administration, Princeton, NJ 08540, USA.

[6]Key Laboratory of Regional Climate-Environment for Temperate East Asia, Institute of Atmospheric Physics, Chinese Academy of Sciences, Beijing, China.

[7]Department of Civil and Environmental Engineering, University of Florence, Firenze 50139, Italy.

Number	Model	Institute
1	ACCESS-CM2	Commonwealth Scientific and Industrial Research Organization (CSIRO)-Australian Research Council Centre of Excellence for Climate System Science (ARCCSS), Australia
2	ACCESS-ESM1-5	Commonwealth Scientific and Industrial Research Organization (CSIRO), Australia
3	BCC-CSM2-MR	National Climate Center, China Meteorological Administration
4	CanESM5	Canadian Centre for Climate Modeling and Analysis (CCCma), Canada
5	EC-Earth3-Veg	EC-Earth-Consortium, Europe
6	GISS-E2-1-G	NASA Goddard Institute for Space Studies (NASA-GISS), USA
7	IPSL-CM6A-LR	Institute Pierre-Simon Laplace (IPSL), France
8	MIROC6	Model for Interdisciplinary Research on Climate (MIROC), Japan
9	MRI-ESM2-0	Meteorological Research Institute (MRI), Japan
10	MPI-ESM-1-2-HR	Deutsches Klimarechenzentrum (DKRZ), Germany
11	MPI-ESM1-2-LR	Max Planck Institute for Meteorology (MPI-M), Germany

Table S1. List of the CMIP6 models used in this study.

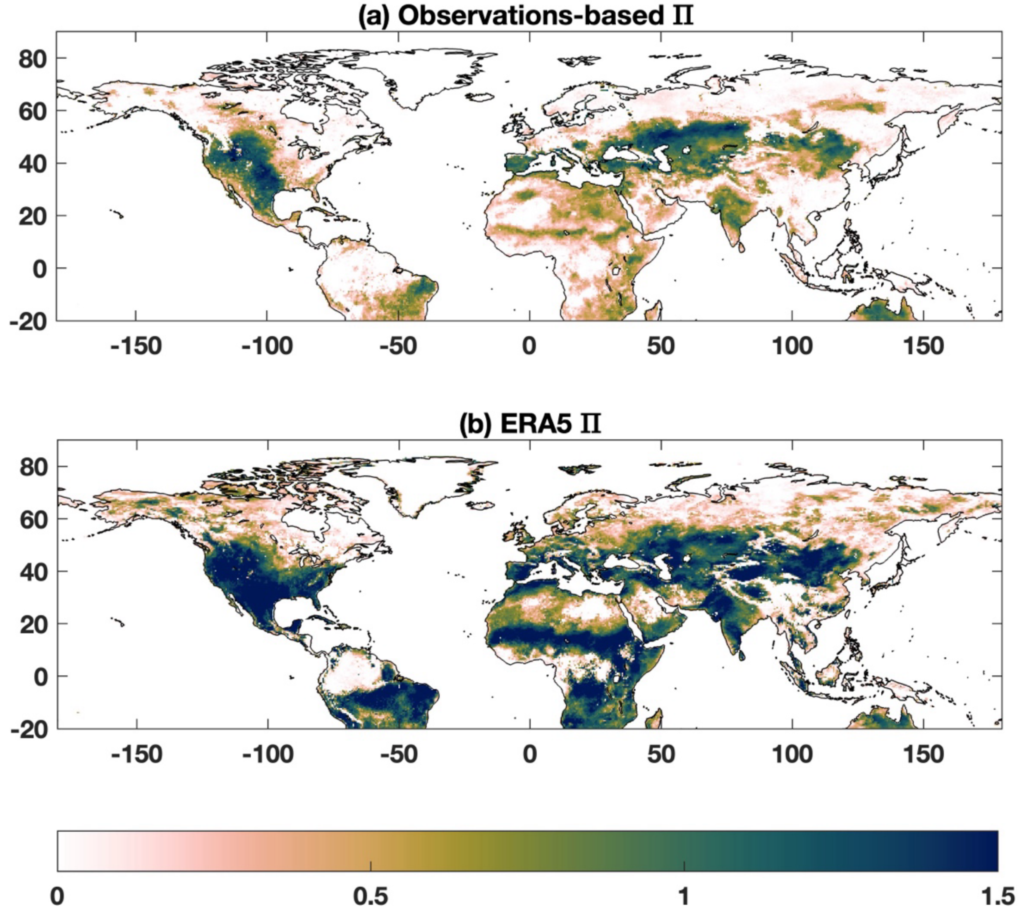

Fig. S1 | Observation- and reanalysis-based boreal summer SM–T coupling. The SM–T coupling in boreal summer (June, July, August) based on the Π diagnostic calculated using (a) T from CRU T and E from GLEAM E, and (b) ERA5 reanalysis outputs T and E during the historical period (1981–2014).

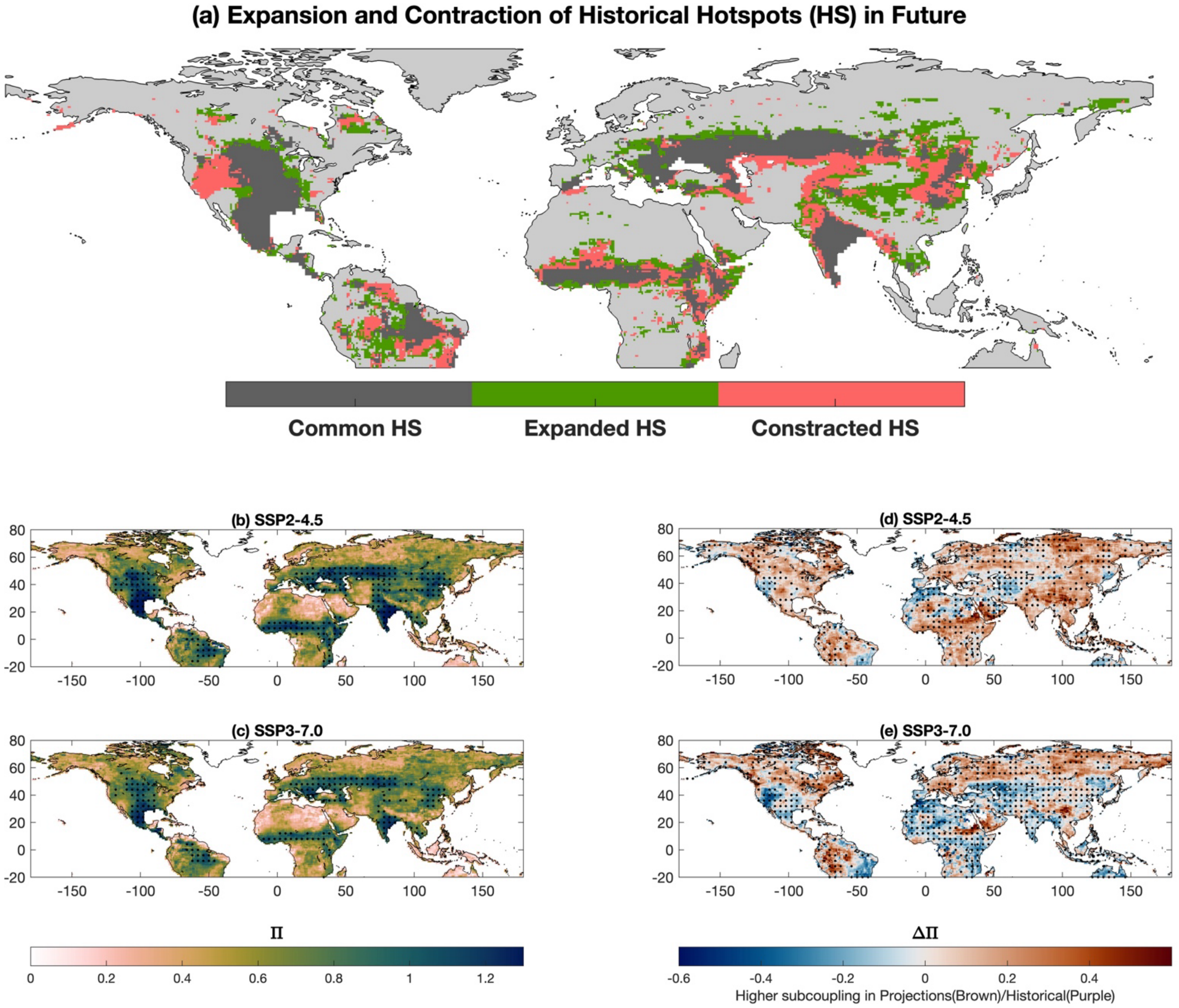

Fig. S2 | The SM–T coupling and projected changes in boreal summer. **a**, Expansion (green) and contraction (red) of the historical SM–T coupling hotspots (HS) based on SSP1-2.6 and SSP5-8.5. Common HS refers to hotspots present in both the historical and future (SSP1-2.6 and SSP5-8.5) periods. Ensemble mean of Π from CMIP6 projections (2015–2100) under **b**, SSP2-4.5 and **c**, SSP3-7.0. The dotted regions (i.e., the coupling 'hotspots') in Fig. 1a-e indicate where more than 70% of the models agree on Π >0.7. Change in Π (i.e., $\Delta\Pi$) during the period 2015–2100 under **d**, SSP2-4.5 and **e**, SSP3-7.0 relative to 1981–2014. Dots indicate regions where at least 70% of the models agree on the sign of the change in Π.

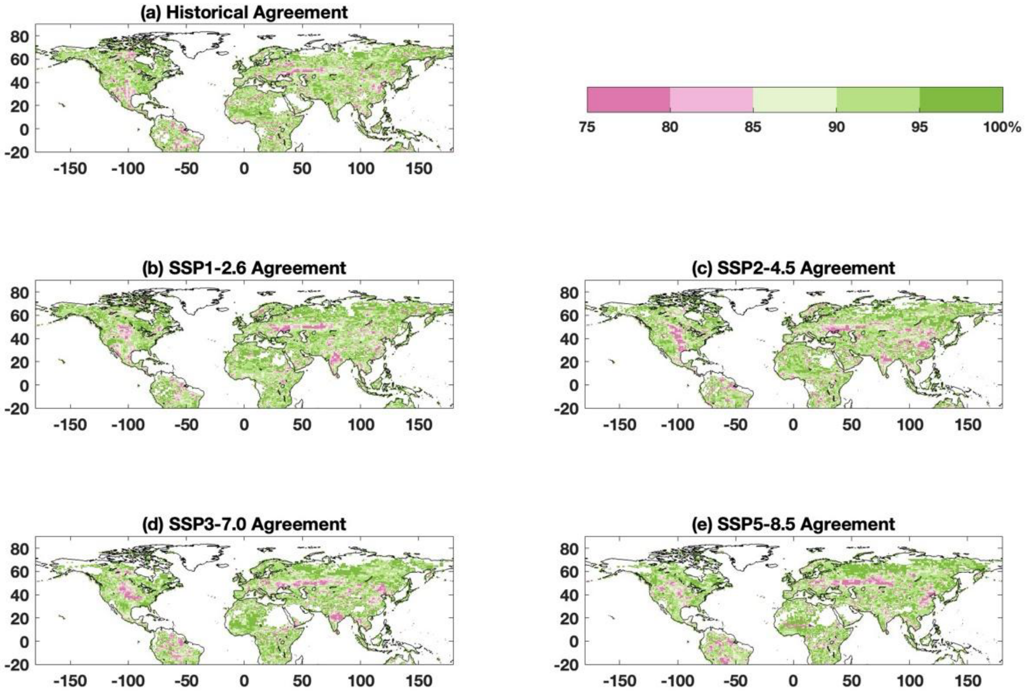

Fig. S3 | Inter–model agreement on Π distribution. Similarities maps of for Π results across the models for each one of the warming scenarios considered. White regions where results are not statistically significant. Similarities are computed based on inter–model variances per pixel, where higher(lower) variances are attributed to lower(higher) similarity in percentages[2].

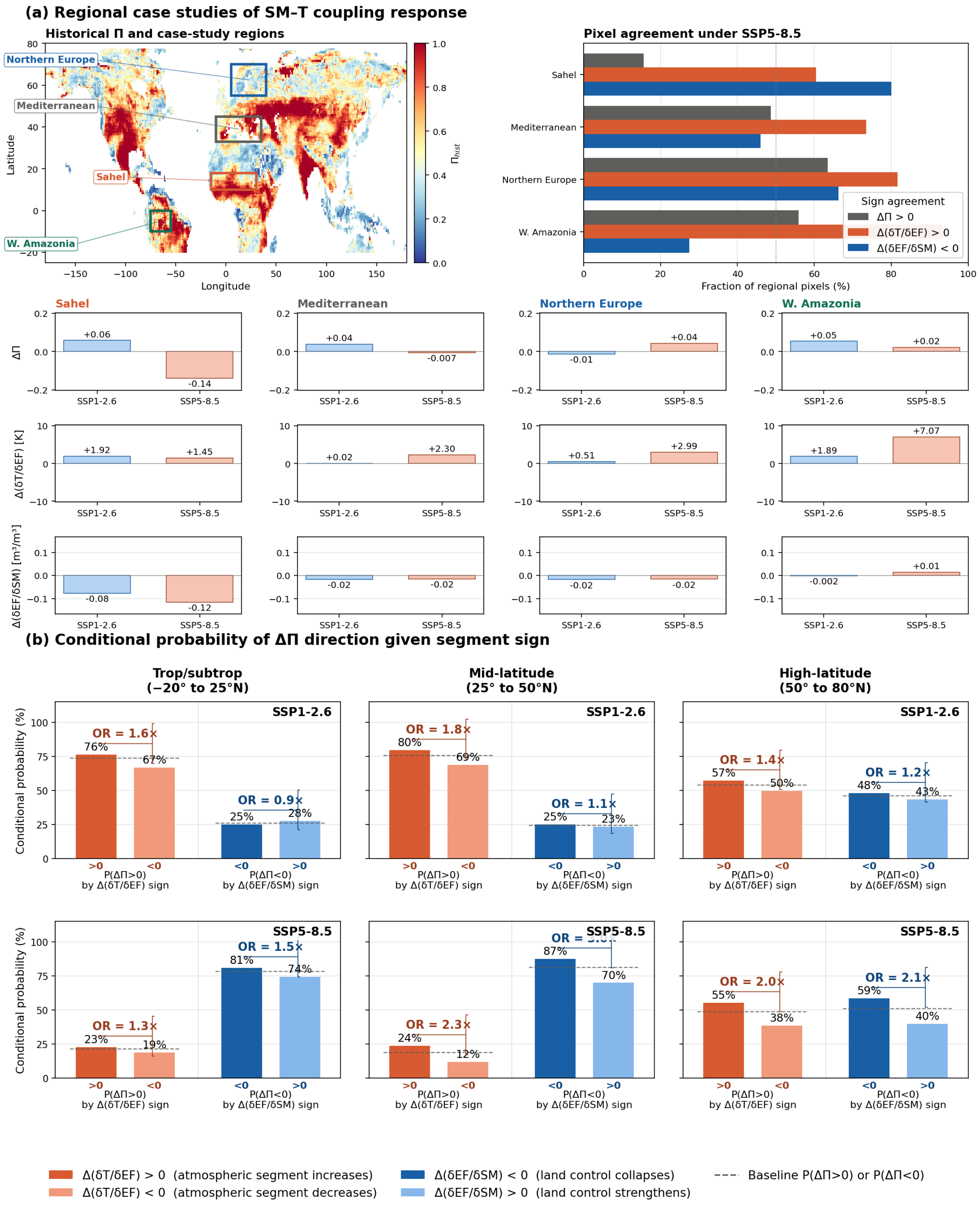

Fig. S4 | Regional case studies and conditional probability analysis of segment-driven Soil moisture–Temperature coupling change ($\Delta\Pi$). (a) Regional case studies for four canonical regions spanning the taxonomy of coupling responses: Sahel (weakening hotspot), Mediterranean (persistent hotspot), Northern Europe (emerging mid-to-high-latitude hotspot), and Western Amazonia (emerging intertropical hotspot). Top-left, historical ensemble-mean Π with bounding boxes of the four regions overlaid. Top-right, fraction of

regional pixels under SSP5-8.5 that exhibit $\Delta\Pi > 0$, $\Delta(\delta T/\delta EF) > 0$, and $\Delta(\delta EF/\delta SM) < 0$, indicating spatial coherence of the regional response. Bottom three rows, regional-mean values of $\Delta\Pi$, $\Delta(\delta T/\delta EF)$, and $\Delta(\delta EF/\delta SM)$ under SSP1-2.6 (blue) and SSP5-8.5 (orange) for each region; numeric labels show the actual regional means. **(b)** Conditional probability analysis applied to pixels falling within the top 60% of $|\Pi \text{hist}|$ (historical SM–T transition belt) and, within that subset, the top 60% of $|\Delta\Pi|$ within each latitude band — the same two-step filter as the main-text Fig. 2g–h analysis. Three latitude bands are shown: tropical/subtropical (−20° to 25°N), mid-latitude (25° to 50°N), and high-latitude (50° to 80°N), under SSP1-2.6 (top row) and SSP5-8.5 (bottom row). Within each panel, the left bar pair shows $P(\Delta\Pi > 0)$ conditional on the sign of $\Delta(\delta T/\delta EF)$ (atmospheric pathway), and the right bar pair shows $P(\Delta\Pi < 0)$ conditional on the sign of $\Delta(\delta EF/\delta SM)$ (land pathway). Dashed horizontal lines indicate the unconditional baseline probabilities $P(\Delta\Pi > 0)$ and $P(\Delta\Pi < 0)$ within each filtered subset. The odds ratios (OR) above each pair (computed with a continuity correction of 10^{-6}) quantify how much the segment sign shifts the odds of $\Delta\Pi$ direction relative to the alternative segment sign. Under SSP5-8.5, both pathways are diagnostically informative across all bands (OR = 1.3–3.0×); under SSP1-2.6, the atmospheric pathway is consistently engaged (OR = 1.4–1.8×) but the land pathway is statistically absent (OR ≈ 0.9–1.2×), indicating that strong warming is required to push regions across the soil moisture regime thresholds at which the land segment becomes diagnostic.

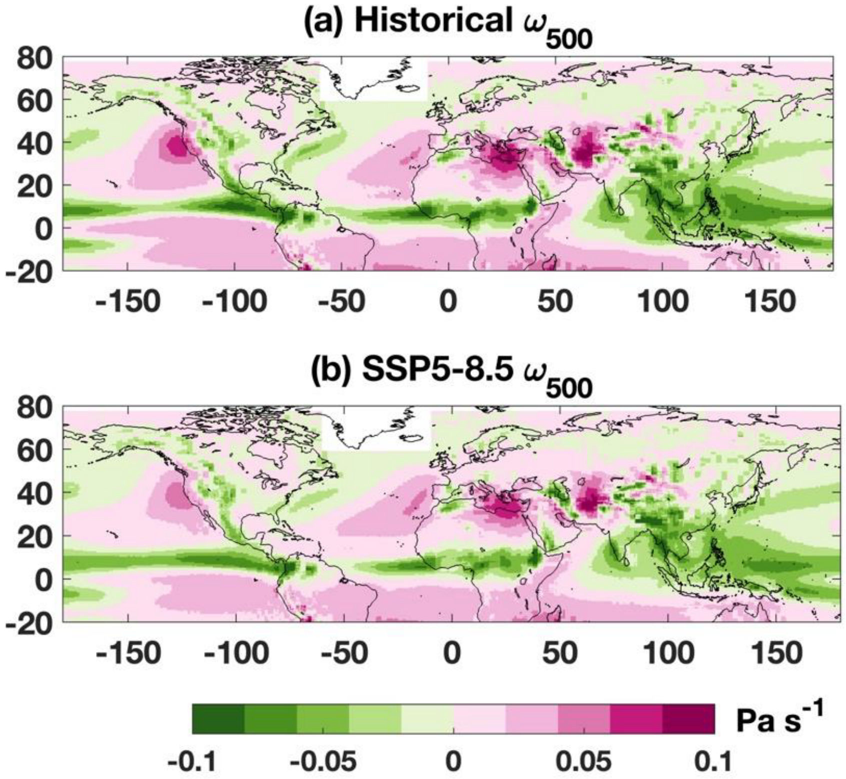

Fig. S5 | Historical and SSP5-8.5 mean vertical wind velocity for boreal summer. (a) Historical vertical wind velocity at the 500hPa level. Green denotes ascents and magenta denotes descents. Greenland is masked out. (b) same as (a) but for SSP5-8.5.

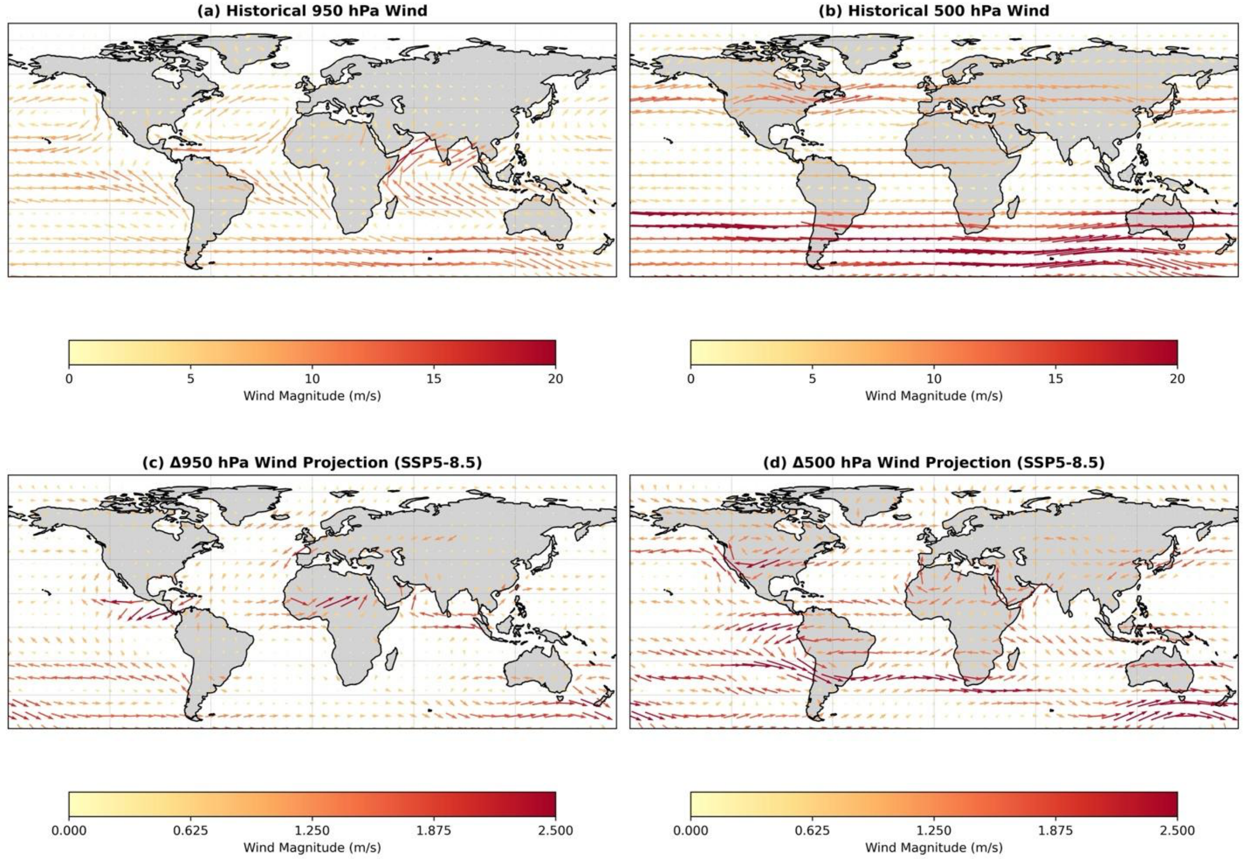

Fig. S6 | Horizontal wind velocity for boreal summer for the 950hPa and the 500hPa level. The horizontal winds at the 950hPa and the 500hPa level for the historical period is represented on **a** and **b**, respectively. Change in the future SSP5-8.5 horizontal winds relative to the historical period at **c,** 950hPa and **d**, 500hPa levels. Arrows in **a**–**d** are drawn at relative lengths. Color gradients represent wind magnitudes.

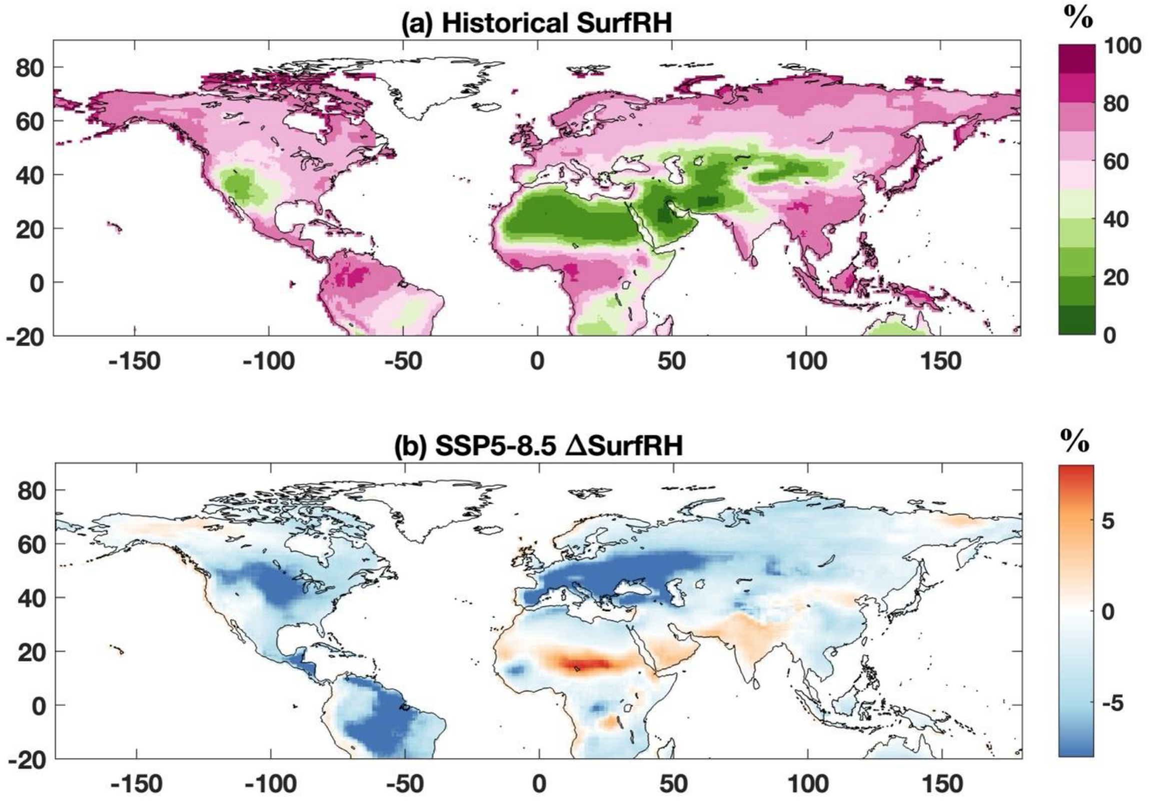

Fig. S7 | Near surface relative humidity for the boreal summer. (a) Historical near surface relative humidity for the historical baseline period. (b) Change in future near surface relative humidity (ΔRH) for SSP5-8.5 relative to the historical period.

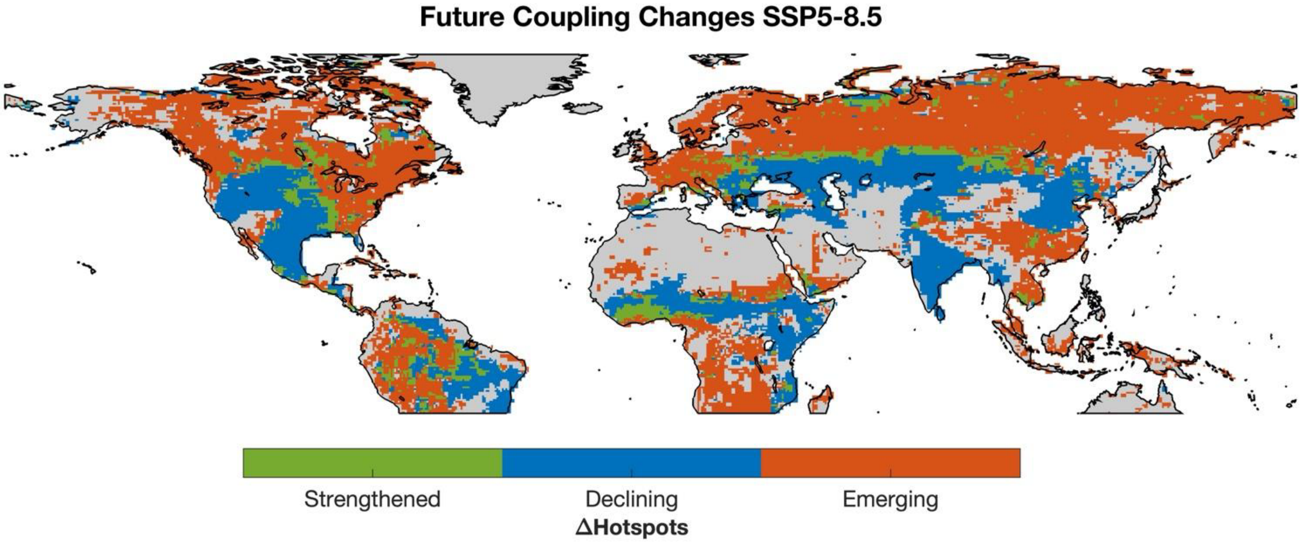

Figure S8 | Overview of the types of changes in the SM–T coupling in the future for SSP5-8.5. Regional responses are three-fold: (1) hotspot regions where the coupling remains strong in the future (green), (2) regions where hotspots are declining (blue) and (3) regions where the coupling is emerging (red).

Supplementary References

1 Alkama, R. *et al.* Vegetation-based climate mitigation in a warmer and greener World. *Nature Communications* **13**, 606 (2022). https://doi.org/10.1038/s41467-022-28305-9

2 Hagan, D. F. T. *et al.* Contrasting ecosystem constraints on seasonal terrestrial CO2 and mean surface air temperature causality projections by the end of the 21st century. *Environmental Research Letters* **17**, 124019 (2022). https://doi.org/10.1088/1748-9326/aca551

3 Denissen, J. M. C., Teuling, A. J., Reichstein, M. & Orth, R. Critical Soil Moisture Derived From Satellite Observations Over Europe. *Journal of Geophysical Research: Atmospheres* **125**, e2019JD031672 (2020). https://doi.org/https://doi.org/10.1029/2019JD031672
