# Supplementary material for: Divergent regional responses of soil moisture-air temperature coupling under future climate scenarios
Source: Nat Commun. 2026 Jun 8;17:7311. doi: 10.1038/s41467-026-74040-w (PMC13402682; doi:10.1038/s41467-026-74040-w)
Supplement: Supplementary file 2 — Transparent Peer Review file [file 41467_2026_74040_MOESM2_ESM.pdf]

nature portfolio

Peer Review File

Divergent regional responses of soil moisture-air temperature coupling under future climate scenarios

Corresponding Author: Dr Daniel Hagan

This file contains all reviewer reports in order by version, followed by all author rebuttals in order by version.

Attachments originally included by the reviewers as part of their assessment can be found at the end of this file.

Version 0:

Reviewer comments:

Reviewer #1

(Remarks to the Author)
Comments on the manuscript “Regional bifurcations of soil moisture–air temperature coupling under future climate warming scenarios” by Daniel F.T. Hagan, Guojie Wang, Alan T. Kennedy-Asser, João L. Geirinhas, Kirsten L. Findell, Mingxing Li, Chenxia Zhu, Shijie Li, Diego G. Miralles.

This study examines the response of soil moisture-temperature (SM–T) coupling under various emission scenarios. The authors combined observational, reanalysis, and model outputs to analyze the historical and projected changes in SM-T coupling. I believe this work would be a good contribution to our knowledge of land-atmosphere processes during climate change. However, while I think the analysis and methods used are appropriate, the manuscript has several issues, including a failure to address some questions. I believe this work might be considered for publication in Nature Communications, after a major revision. Please see below my comments and suggestions.

Major observations:
1. The authors used 11 CMIP6 models, which they listed in the supplementary materials. However, they don’t explain why they used these models, and do not even mention the members they used from each of them. What is the criterion the authors used to select these models? Although CMIP6 has over 30 models, the authors do not explain why they selected the models used in this work. I suggest including a more comprehensive explanation of how they selected the CMIP6 models they used.

2. The authors emphasize several times that changes in SM-T coupling influence climate extremes, such as heat waves and droughts. While they cite the papers with those findings, they do not explain the processes underpinning this increased risk of climate extremes from their results. They must explain further why changes in SM-T coupling would lead to an increased risk of heatwaves in certain regions. I did not see a comprehensive discussion about this implication, other than “An important implication of these findings is a higher likelihood of heatwaves and compound dry–hot events modulated by SM–T coupling changes in the emerging hotspot regions [20]. Under these conditions, temperature increases are expected to be amplified by land dry-outs.” For example, which regions may experience a higher risk of heatwaves?

3. Another question about this work concerns how the author addressed potential biases related to land used for agriculture, much of which is irrigated. How could this artificial soil moisture influence the results? Is there a way to estimate the margin of error due to this issue? The US central plains, for example, are mostly dedicated to agriculture, unlike other regions with high SM-T coupling, such as eastern equatorial Africa. As the authors stated, regions with strong SM-T coupling are mostly transition zones between dry and wet regions; however, many of these regions are also dedicated to agriculture and are irrigated. Some work about this include:

a) https://journals.ametsoc.org/view/journals/clim/30/10/jcli-d-15-0706.1.xml?alreadyAuthRedirecting

b) https://journals.ametsoc.org/view/journals/hydr/25/7/JHM-D-23-0187.1.xml

4. Not exactly an issue, but it would be great to compare the changes in SM-T coupling between warming scenarios and pi-control simulations to isolate the influence of climate change.

Minor:
L17-18. "Global hotspot regions where soil moisture (SM) controls air temperature (T) may shift and change in intensity under climate change, influencing the occurrence of hydroclimatic extreme events." I suggest mentioning whether these SM-T coupling patterns have been observed in instrumental records or only in GCMs.

L41. For land-atmosphere coupling and drought (and maybe for the overall results from their work), the authors may consider the following paper for the northeastern US:
https://agupubs.onlinelibrary.wiley.com/doi/full/10.1029/2021JD034740

L58. "In fact, these changes have already been detected in observations…" Like, for example? It would be great to see some examples of those changes here without reading the cited references. That would also make clearer the following clause, "while climate models predict their further intensification in the future, particularly in the Northern Hemisphere." It is unclear what the authors refer to as "their further intensification" without an example of the observed changes.

L65. Replace "foresee" by "expected"

L71-73. This sentence is unclear to me. For example, to what kind of sensitivity do the authors refer here? I also do not see any reference backing up these claims. I suggest a better organization of this sentence so it would be easier to follow the idea the authors want to say.

L74-75. "As such, changes in coupling could arise from changes in either or both components, particularly in response to global warming." How certain are the authors about this statement? As in my previous suggestion, I think this sentence would benefit from at least a reference. I would also suggest replacing "global warming" with "climate change", since (as the authors mentioned above) SM-T coupling depends on the projected changes in precipitation (i.e., not only temperature).

L82. Replace "have concentrated" by "have occurred"

L104-105. "revealing the general skill of CMIP6 climate models to represent this coupling." Agreed. However, these are only 11 CMIP6 models out of over 30 models. Things would be clearer if the authors explained why they selected these models.

L111 and Figure 1. Since the authors refer to changes in SM-T coupling between the equator and 40° North, it would be helpful to overlay these lines on the maps. Some readers may not be familiar with this concept.

L123. "…and the emergence of coupling over some intertropical humid regions, such as Amazonia and…" This is quite confusing to me. The authors say "the emergence of coupling over… Amazonia." However, per panels a-c, there is a strong SM-T coupling in the Amazon. Perhaps the authors mean "the strengthening" instead of "the emergence"?

L155. Delete one of the dots

L196. Add a comma before which

L222. Maybe say "convergence" and "divergence" instead of "ascent" and "descent"

Figure 4. It is unclear if by "strong warming" the author refers to SSP5-8.5 or to both of the emission scenarios they used. If so, I suggest saying this explicitly, since they showed different SM-T coupling pattern changes depending on the emission scenario. I assume they are referring to SSP5-8.5, but this could confuse some readers.

L271-272. Again, the author should specify if by "global warming" they mean either SSP5-8.5, SSP1-2.6, or both, as this is confusing. If this is the model to explain the changes in SM-T coupling with SSP5-8.5, what would be the one for SSP1-2.6? Why

L334-335. This is a minor suggestion, but it would be great if the author explain why they used the Priestley and Taylor to calculate Ep.

L351. "We follow the approach from previous studies [comma here] which suggested…" or "We follow the approach from previous studies that suggested…"

L351. To which previous studies the authors are referring here?

L358-359. "The first component can be assumed to be unitless, while the second component is in K." I might be wrong, but is not the opposite here? E.g., $\delta T = \delta T/\delta EF$, $\delta EF/\delta\theta$

Reviewer #2

(Remarks to the Author)
Review of manuscript entitled "Regional bifurcations of soil moisture–air temperature coupling under future climate warming scenarios" by Daniel F.T. Hagan, Guojie Wang, Alan T. Kennedy-Asser, João L. Geirinhas, Kirsten L. Findell, Mingxing Li, Chenxia Zhu, Shijie Li, and Diego G. Miralles
This manuscript presents an important investigation into the projected future changes in global hot-spots of soil moisture–air temperature (SM–T) coupling and the mechanisms driving these changes. Understanding how SM–T coupling will evolve under future climate warming is a critical scientific question, given its implications for heat extremes, land–atmosphere feedbacks, and regional climate impacts. I commend the authors for selecting this timely and highly relevant topic.
The manuscript is thoroughly written, with a clear and well-structured Abstract, Introduction, Results, and Discussion sections. The scientific content is rigorous, the methodology is sound, and the findings provide novel insights into regional bifurcations of SM–T coupling under warming scenarios. The figures are effective in illustrating the key results, and the discussion successfully links the outcomes to broader climate processes.
Overall, the paper makes a valuable contribution to advancing our understanding of land–atmosphere interactions in a changing climate. I find the manuscript to be of high scientific quality and well-suited for publication. Therefore, I strongly recommend acceptance of this manuscript.

Reviewer #3

(Remarks to the Author)
Overall comments
The paper looks at the impact of climate change on land-atmosphere coupling, which is a major source of uncertainty in 2m-temperature, projections, and the influence of SSP on these changes. It is found that high and low emissions pathways have quite different impacts on the change in SM-T coupling with coupling tending to be enhanced under low emissions pathways, such as SSP-1-2.6. In contrast, a more complex response is seen under high emissions pathways, such as SSP-5-8.5, where coupling is reduced in existing mid-latitude and sub-tropical hotspots due to a reduction in SM (due to an expanded Hadley circulation and higher precip), but enhanced at higher latitude as a result of soil-moisture drying there.

Overall, I found the results in the paper interesting, the conclusions plausible and a of significance. However, I found there was a general lack of statistical testing, making it hard to completely assess the robustness of the findings. For example, none of the maps of changes under the various emissions pathways give any measure of agreement in the sign of the change or it's statistical significance. As a result, one is left wondering if all the models have the same behaviour, or just one or two. There are also some missing methodological details that would make it hard to reproduce form this manuscript (but which can be easily corrected).

The paper would also benefit from more careful editorial work to improve the text and make it easier to follow. I provide some examples but don't make a comprehensive list.

I wasn't very keen on using the term "bifurcation" to describe different regional responses to climate change or to differences between emissions pathways. It sounds grander than "regional differences" or "differences between emission pathways" but the term has quite a specific definition in dynamical systems theory, and I found it a bit distracting. This terminology may also be physically wrong, for example, it may be that under SSP-5-8.5 the models undergo a strengthening in the existing hotspots in the early 21st century, before this weakening of existing hotspots and northward expansion (seen at the end of century) due to dynamical changes takes over. This would make it more of a transitional state than a bifurcation. This could be checked by looking at the earlier decades of the SSP-5-8.5 projections and would provide an indication of what the early warnings signs are of such a transition.

Specific comments
L21 (and elsewhere): "response of coupling to warming" suggest changing to "response of coupling to climate change" or to anthropogenic emissions.

L27: is "disproportionate" the right choice of word?

Fig 1: I think it would be useful to indicate some measure of agreement in the ensemble mean changes in , and also include a contour of in the historical runs in Fig1d and e so that it is easy to see how the changes in correspond to the present-day hotspots. Also for Fig S2.

L102-110: I found this paragraph a somewhat uncritical appraisal of the CMIP6 ensemble's ability to capture the strength of land-atmosphere coupling. It would be helpful if the colour bar in Fig 1a-c matched with Fig S1, but also useful to include a bias map for the metric in Fig S1, so which highlights the differences. By eye, there seems to be a general tendency to overestimate the coupling strength, compared to Fig S1a as also seen in Hsu and Dirmeyer (2022) for CMIP5 and also Day et al. (2025), for seasonal models. These studies both show that climate models tending to be in a soil-limited evaporation regime over a larger area of the mid-latitudes than the real world. Not having the historical runs in the correct SM-E regime will have some important implications for the ability to predict transitions from one regime to the other, so this caveat needs to be more critically discussed as a limitation of the study.

Fig S2: also need to make colourbars consistent with Fig's 1 and Fig S1.

L111: “weakening” to “weakened”

L153: This is very similar to the approach of Dirmeyer (2011) and Dirmeyer et al. (2014) who described a terrestrial and atmospheric leg to the coupling. Suggest citing these papers here and framing in terms of terrestrial-leg and atmospheric-leg.

L161: Rather than saying “negated” just put a minus in front of the expression.

Fig2: I think it would be better to use the same colour scheme for the lines in sub panels g and h, since I think they are showing the same variables.

L139-198: I found this section quite hard to follow. It also seems that the metrics used to measure the coupling strength in this section seem not to capture the same changes seen in the previous section with the   metric (figs 1d & e vs. Fig 2c-f). The section seems to be framed in terms of understanding changes in   with the two derivatives, but they measure quite different things. As a result, I think this section confuses things rather than explaining them. Then Fig S4 shows something quite similar to the change in .

L119: It would seem more appropriate to show the agreement in the change in coupling here, instead Fig S3 shows the agreement in the absolute coupling strength.

Fig 3: The maps are quite hard to read, particularly the contours showing the change in . I also thought it would be clearer to see where the meridional averages were coming from if the latitude ticks were aligned between a and c and b and d.

L299-322: It says that monthly data were used for obs but seasonal for CMIP models. Were they averaged to the same frequency before metrics were calculated?

L339: It says here that G was calculated as a fixed fraction of the net radiation. I can imagine this has a non-trivial impact when moving from one climate to another. For example if the future climate has lower wind, and reduced turbulent exchange, G will be higher (but not according to this calculation).

It should also be mentioned in the data section what depth of soil-moisture was used from CMIP and ERA5/GLEAM.

Version 1:

Reviewer comments:

Reviewer #1

(Remarks to the Author)
Comments on the manuscript “Divergent regional responses of soil moisture-air temperature coupling under future climate scenarios” by Daniel F.T. Hagan, Guojie Wang, Alan T. Kennedy-Asser, João L. Geirinhas, Kirsten L. Findell, Mingxing Li, Chenxia Zhu, Shijie Li, and Diego G. Miralles.

After a careful revision, I confirm that the authors have addressed most of my comments in this revised manuscript. I therefore suggest accepting the manuscript for publication in Nature Communications in its present form.

(Remarks on code availability)

Reviewer #3

(Remarks to the Author)

[Editorial Note: See end of file]

(Remarks on code availability)

Version 2:

Reviewer comments:

Reviewer #3

(Remarks to the Author)
I would like to thank the authors for the changes to the text and figures, but in my view the conclusions are still not completely well justified from the analysis.

The main outstanding issue for me is that the section on land and atmosphere segments is still hard to follow, and in particular it is hard to both understand Fig 2 and reconcile it with results seen in Fig 1 and in the summary of processes seen in the schematic in Fig 4. This has not really changes though the previous iterations of the manuscript.

In this version it is also still not clear why the coupling metrics are plotted against soil-moisture in the historical runs in Figs 2b,g and h. The explanation in the text: "We analyse both segments across historical SM climatology since patterns of strong SM–T couplings are well known across these SM regimes" is not really illuminating about the motivation of presenting it like this or a sufficient explanation. In what way are they 'well known' in? The authors provided detailed explanation of the metrics in the last response to my comments, but the important thing to explain is how plotting them like this (binning with soil moisture across models and regions) informs the physical interpretation of change in coupling regimes. Whether a location is in an energy or soil limited ET regime will depend not just on the soil moisture, but also the PWP and critical values of soil moisture. As a result grouping it like this is not really grouping by ET regime, so why is it shown like this? If this has been done elsewhere and gives some particular insight then say so and explain what it is. Otherwise, please consider presenting in another way, such as binning by latitude to enhance interpretation of the northward shift in coupling zones.

In my view it would be much easier to conclude something from this section if there was some statistical analysis to link these metrics with the changes in hotspot regions. For example what percentage of the weakening (and emerging) hotspots shown in the Fig 4 insert have weakening or strengthening del EF/delSM or delT/delEF. I think this type of contingency table would help to show that this analysis is informative. At the moment I am still not convinced that it is.

It is really not clear without this type of statistical analysis that the neat summary that the clear summary regarding the role of land and atmosphere coupling segments in the regime transitions shown in Fig 4 is supported by the evidence in this section. I don't think the argument that it is non-linear and we wouldn't expect 1-1 agreement is consistent with this clear picture and if things are more complicated, this needs to be stated clearly and understood through additional analysis before it is published.

(Remarks on code availability)

Version 3:

Reviewer comments:

Reviewer #3

(Remarks to the Author)
Firstly I would like to thank the authors for following my suggestion to present the latitudinally averaged coupling metrics and contingency table in Fig2. I think it makes it much clearer why the section is so confusing.

The latitudinal sections of del(dT/dET) and del(dEF/dSM) show quite similar changes for both SSP1-2.6 and SSP5-8.5, i.e. somewhat reduced del(dEF/dSM) and increased del(dT/dET). It seems that the main difference is that the magnitude of del(dT/dET) is different between the scenarios, but the del(dEF/dSM) looks identical between the two. From this it is very hard to understand the large reduction in Pi in the SSP5-8.5 run, in contrast to the increase in the other. If only the segment analysis, not the Pi metric, were presented, I would interpret this as showing an increase in SM-T in both scenarios, driven by an increase in the del(dT/dET) term.

However, this is not what the authors conclude, and the contingency table makes it very clear that there is not a good correspondence between delPi, del(dT/dET) and del(dEF/dSM) at the gridpoint level. For example, for a given gridpoint in SSP-2.6, where either both del(dT/dET) and del(dEF/dSM) are increasing (+,+) or both are decreasing (-,-) the change in Pi is positive or negative for about 50% of grid points respectively, suggesting that variations in these two properties do not explain much of the variability in Pi. As expected I think this makes the clean explanation of the regional divergence hard to justify based on this analysis.

I am not really in a position to explain these differences, this is really up to the authors to do, but It does make it quite hard to conclude much from this section since the metrics show quite different things.

(Remarks on code availability)

Version 4:

Reviewer comments:

Reviewer #1

(Remarks to the Author)

Following careful revision, the authors have successfully addressed all my suggestions and concerns. I recommend accepting the manuscript for publication in Nature Communications in its current form.

(Remarks on code availability)

Open Access This Peer Review File is licensed under a Creative Commons Attribution 4.0 International License, which permits use, sharing, adaptation, distribution and reproduction in any medium or format, as long as you give appropriate credit to the original author(s) and the source, provide a link to the Creative Commons license, and indicate if changes were made.
In cases where reviewers are anonymous, credit should be given to 'Anonymous Referee' and the source.
The images or other third party material in this Peer Review File are included in the article's Creative Commons license, unless indicated otherwise in a credit line to the material. If material is not included in the article's Creative Commons license and your intended use is not permitted by statutory regulation or exceeds the permitted use, you will need to obtain permission directly from the copyright holder.
To view a copy of this license, visit https://creativecommons.org/licenses/by/4.0/

Response to REVIEWER COMMENTS

We are grateful to the reviewers and editorial team for working hard to provide these helpful comments to improve our manuscript. We have attempted to respond to every comment faithfully below. All our responses are in blue fonts for clarity while the changes made in the manuscript's text are marked with yellow highlights.

Reviewer #1 (Remarks to the Author):

Comments on the manuscript "Regional bifurcations of soil moisture–air temperature coupling under future climate warming scenarios" by Daniel F.T. Hagan, Guojie Wang, Alan T. Kennedy-Asser, João L. Geirinhas, Kirsten L. Findell, Mingxing Li, Chenxia Zhu, Shijie Li, Diego G. Miralles.

This study examines the response of soil moisture-temperature (SM–T) coupling under various emission scenarios. The authors combined observational, reanalysis, and model outputs to analyze the historical and projected changes in SM-T coupling. I believe this work would be a good contribution to our knowledge of land-atmosphere processes during climate change. However, while I think the analysis and methods used are appropriate, the manuscript has several issues, including a failure to address some questions. I believe this work might be considered for publication in Nature Communications, after a major revision. Please see below my comments and suggestions.

Major observations:

1. The authors used 11 CMIP6 models, which they listed in the supplementary materials. However, they don't explain why they used these models, and do not even mention the members they used from each of them. What is the criterion the authors used to select these models? Although CMIP6 has over 30 models, the authors do not explain why they selected the models used in this work. I suggest including a more comprehensive explanation of how they selected the CMIP6 models they used.

Response: Thank you for this comment. The models used here were selected based on available variables for all four scenarios for at least, the first two realizations (r1i1p1f1 or r1i1p1f2, where r1i1p1f1 is unavailable), needed for our analysis at the time the study was conducted. This limited the number of models used. We have added more text to the manuscript to clarify this. Lines 313-315 now read:

"*For model consistencies, models with all the variables at the first (or second where the first is absent: r1i1p1f1 or r1i1p1f2) realization for all four scenarios were selected*

(Table S1)."

2. The authors emphasize several times that changes in SM-T coupling influence climate extremes, such as heat waves and droughts. While they cite the papers with those findings, they do not explain the processes underpinning this increased risk of climate extremes from their results. They must explain further why changes in SM-T coupling would lead to an increased risk of heatwaves in certain regions. I did not see a comprehensive discussion about this implication, other than "An important implication of these findings is a higher likelihood of heatwaves and compound dry–hot events modulated by SM–T coupling changes in the emerging hotspot regions [20]. Under these conditions, temperature increases are expected to be amplified by land dry-outs." For example, which regions may experience a higher risk of heatwaves?

Response: Thanks for this comment. The role of the SM–T coupling, where coupling changes could enhance land–atmosphere feedback during heatwaves and droughts, has been extensively presented in earlier studies. Very detailed mechanistic studies and associations of the Pi metric to hotspells have been presented in studies such as Miralles et al. (2012, 2014) and Dirmeyer et al. (2023). What still lacks more research is the nature of these changes. Thus, to better understand how these future extremes would be impacted, we build on these studies to understand the nature of the coupling changes based on the projections, as well as the potential mechanisms which lead to these changes. From these earlier studies, regions that are emerging as stronger Pi regions will be more prone to these extremes as well. We have added some text to further highlight the suggestion of the reviewer. Lines 294-299 now read:

An important implication of these findings is a higher likelihood of heatwaves and compound dry–hot events modulated by SM–T coupling changes in the emerging coupling regions. These appear to be found in humid high latitude and tropical humid areas via a disruptive partitioning of the surface heat fluxes[21]. Under these conditions, temperature increases are expected to be amplified by land dry-outs, which constrain evaporation and increase sensible heat flux, exacerbating heat extremes[3,59].

References

Miralles, D. G., van den Berg, M. J., Teuling, A. J. & de Jeu, R. A. M. Soil moisture-temperature coupling: A multiscale observational analysis. *Geophysical Research Letters* **39** (2012). https://doi.org/https://doi.org/10.1029/2012GL053703

Seneviratne, S. I. *et al.* Investigating soil moisture–climate interactions in a changing climate: A review. *Earth-Science Reviews* **99**, 125-161 (2010).

Chemke, R. & Polvani, L. M. Opposite tropical circulation trends in climate models and in reanalyses. *Nature Geoscience* **12**, 528-532 (2019). https://doi.org/10.1038/s41561-019-0383-x

3. Another question about this work concerns how the author addressed potential biases related to land used for agriculture, much of which is irrigated. How could this artificial soil moisture influence the results? Is there a way to estimate the margin of error due to this issue? The US central plains, for example, are mostly dedicated to agriculture, unlike other regions with high SM-T coupling, such as eastern equatorial Africa. As the authors stated, regions with strong SM-T coupling are mostly transition zones between dry and wet regions; however, many of these regions are also dedicated to agriculture and are irrigated. Some work about this include:

a) https://journals.ametsoc.org/view/journals/clim/30/10/jcli-d-15-0706.1.xml?alreadyAuthRedirecting

b) https://journals.ametsoc.org/view/journals/hydr/25/7/JHM-D-23-0187.1.xml

Response: Thank you for the comment. Indeed, this is becoming an increasingly important area of study in the field, with certainly a lot of uncertainties about the potential impacts or irrigation, namely on the occurrence of local and downwind. Potentially, over a long period of time, regions dominated by dry-hot extremes could even become humid-hot extremes due to irrigation practices. However, this warrants a very detailed study to adequately quantify the impacts. A detailed analysis of this nature is beyond the focus of this study. We are already looking into this direction in other studies which are currently under review. Please find excerpts in this abstract link: https://meetingorganizer.copernicus.org/EGU25/EGU25-7752.html, for which the full manuscript is currently under review. We have added some text to include this. Lines 299-302 now read:

Additionally, understanding SM–T coupling changes is crucial in formulating adaptation and mitigation strategies in agriculture to ensure food security and water availability in hotspot regions. To adequately quantify this, human activities such as irrigation and land use and cover changes may also have to be considered to fully capture changes in the coupling, especially in the terrestrial segment.

4. Not exactly an issue, but it would be great to compare the changes in SM-T coupling between warming scenarios and pi-control simulations to isolate the influence of climate change.
Response: Thank you for this comment. We believe this is something we can do by referencing the study by Hsu et al (2023: https://doi.org/10.1038/s41467-023-36794-5), which notably includes humid high latitude regions which transition into subhumid conditions. We have referenced this study appropriately in the manuscript. Lines 168-172 now read:

Figure 2c–d indicate a future weakening of δEF/δθ in the historical transitional regions associated with hotspots, particularly for SSP5-8.5, and an increase in higher-latitude humid regions like Western Europe. The latter indicates a likely shift towards sub-humid conditions and, thus, a more responsive relationship between EF and SM (Fig. 2a). This is consistent with recent findings pointing to the expansion of sub-humid climate zones at high latitudes[30,53].

References

Li, M., Wu, P., Sexton, D. M. H. & Ma, Z. Potential shifts in climate zones under a future global warming scenario using soil moisture classification. *Climate Dynamics* **56**, 2071-2092 (2021). https://doi.org/10.1007/s00382-020-05576-w

Dirmeyer, P. A., Wang, Z., Mbuh, M. J. & Norton, H. E. Intensified land surface control on boundary layer growth in a changing climate. *Geophysical Research Letters* **41**, 1290-1294 (2014). https://doi.org/https://doi.org/10.1002/2013GL058826

Minor:
L17-18. “Global may shift and change in intensity under climate change, influencing the occurrence of hydroclimatic extreme events.” I suggest mentioning whether these SM-T coupling patterns have been observed in instrumental records or only in GCMs.

Response: Thank you for this comment. While these long-term changes at a global scale have not been explicitly shown from observational records due to their limited coverage in space and time, some regional studies have shown changes from near-observation datasets such as ERA Interim and GLEAM (e.g. Yuan et al. (2020:) and Lu et al (under review in the Journal of Climate)). Studies like Hirshi et al. (2014: http://dx.doi.org/10.1016/j.rse.2014.08.030) used the CCI observations to correlate soil moisture with temperature, and found some decoupling in land–atmosphere coupling under extreme hot events, which was one of the main motivations for looking into different warming scenarios. Nonetheless, this is indeed, a chance for future studies to take advantage of recent increases in observational records to find these signals. We have included this in the text. It now reads:

Climate models project that hotspot regions where soil moisture (SM) influences air temperature (T) will shift and change in strength, affecting the occurrence of droughts and heatwaves.

L41. For land-atmosphere coupling and drought (and maybe for the overall results from their work), the authors may consider the following paper for the northeastern US: https://agupubs.onlinelibrary.wiley.com/doi/full/10.1029/2021JD034740

Response: Thank you. The reference has been included.

L58. “In fact, these changes have already been detected in observations…” Like, for example? It would be great to see some examples of those changes here without reading the cited references. That would also make clearer the following clause, “while climate models predict their further intensification in the future, particularly in the Northern Hemisphere.” It is unclear what the authors refer to as “their further intensification” without an example of the observed changes.

Response: The sentence refers to the changes already elaborated in earlier parts of the paragraph. We have made the changes to the text to highlight this. It now reads:

In fact, the above-mentioned changes have already been detected in observations[24,34,35], and climate models predict their further intensification in the future, particularly in the Northern Hemisphere[25].

References

Liu, J., Song, M., Hu, Y. & Ren, X. Changes in the strength and width of the Hadley Circulation since 1871. *Clim. Past* **8**, 1169-1175 (2012). https://doi.org/10.5194/cp-8-1169-2012

Lu, J., Vecchi, G. A. & Reichler, T. Expansion of the Hadley cell under global warming. *Geophysical Research Letters* **34** (2007). https://doi.org/https://doi.org/10.1029/2006GL028443

Lu, J., Deser, C. & Reichler, T. Cause of the widening of the tropical belt since 1958. *Geophysical Research Letters* **36** (2009). https://doi.org/https://doi.org/10.1029/2008GL036076

Seidel, D. J., Fu, Q., Randel, W. J. & Reichler, T. J. Widening of the tropical belt in a changing climate. *Nature Geoscience* **1**, 21-24 (2008). https://doi.org/10.1038/ngeo.2007.38

L65. Replace “foresee” by “expected”

Response: Done.

L71-73. This sentence is unclear to me. For example, to what kind of sensitivity do the authors refer here? I also do not see any reference backing up these claims. I suggest a better organization of this sentence so it would be easier to follow the idea the authors want to say.

Response: We have included a reference and edited the text. It now reads:

These challenges arise from the complex and nonlinear nature of the SM–T coupling, which results from the combined contribution of two segments, known as the terrestrial and the atmospheric segments[43,48,49]. The first relates to the influence of soil moisture on the surface energy balance, while the second addresses the ultimate impact of changes in the surface energy balance on air temperature.

References

Schwingshackl, C., Hirschi, M. & Seneviratne, S. I. Quantifying Spatiotemporal Variations of Soil Moisture Control on Surface Energy Balance and Near-Surface Air Temperature. *Journal of Climate* **30**, 7105-7124 (2017). https://doi.org/https://doi.org/10.1175/JCLI-D-16-0727.1

Dirmeyer, P. A. The terrestrial segment of soil moisture–climate coupling. *Geophysical Research Letters* **38** (2011). https://doi.org/https://doi.org/10.1029/2011GL048268

Dirmeyer, P. A., Jin, Y., Singh, B. & Yan, X. Trends in Land–Atmosphere Interactions from CMIP5 Simulations. *Journal of Hydrometeorology* **14**, 829-849 (2013). https://doi.org/https://doi.org/10.1175/JHM-D-12-0107.1

L74-75. "As such, changes in coupling could arise from changes in either or both components, particularly in response to global warming." How certain are the authors about this statement? As in my previous suggestion, I think this sentence would benefit from at least a reference. I would also suggest replacing "global warming" with "climate change", since (as the authors mentioned above) SM-T coupling depends on the projected changes in precipitation (i.e., not only temperature).

Response: We have changed it.

As such, changes in coupling in response to different scenarios could arise from changes in either or both segments[43,48].

References

Schwingshackl, C., Hirschi, M. & Seneviratne, S. I. Quantifying Spatiotemporal Variations of Soil Moisture Control on Surface Energy Balance and Near-Surface Air Temperature. *Journal of Climate* **30**, 7105-7124 (2017). https://doi.org/https://doi.org/10.1175/JCLI-D-16-0727.1

Dirmeyer, P. A. The terrestrial segment of soil moisture–climate coupling. *Geophysical Research Letters* **38** (2011). https://doi.org/https://doi.org/10.1029/2011GL048268

L82. Replace "have concentrated" by "have occurred"

Response: Done.

L104-105. "revealing the general skill of CMIP6 climate models to represent this coupling." Agreed. However, these are only 11 CMIP6 models out of over 30 models. Things would be clearer if the authors explained why they selected these models.

Response: Please see the response to the first major comment.

L111 and Figure 1. Since the authors refer to changes in SM-T coupling between the equator and 40° North, it would be helpful to overlay these lines on the maps. Some readers may not be familiar with this concept.

Response: Thank you for this comment. We have updated Fig. 1 to make this possible by including the coordinate information.

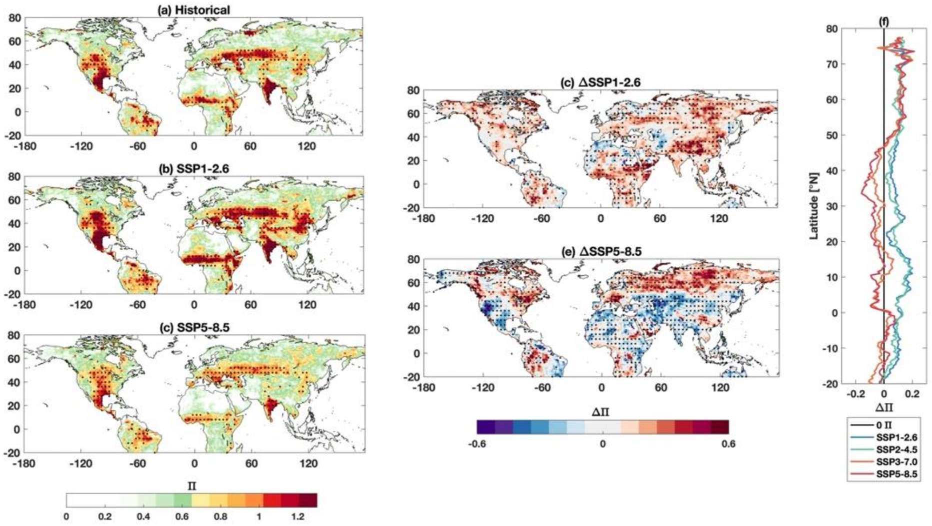

Fig. 1 | SM–T coupling and projected changes in boreal summer. a, SM–T coupling (Π) based on the CMIP6 ensemble mean of the historical period (1981–2014). **b-c**, Same but for future projections (2066–2100) under **b**, SSP1-2.6 and **c**, SSP5-8.5. Dots indicate hotspot regions where at least 8 of the models agree on $\Pi > 0.75$. **d–e**, Change in SM–T coupling (i.e., $\Delta\Pi$) during 2066–2100 relative to 1981–2014 under **d**, SSP1-2.6 and **e**, SSP5-8.5. Dots indicate regions where at least 8 of the models agree on the sign of the change in Π. **f**, Mean latitudinal profile of $\Delta\Pi$ for the future (SSP1-2.6, SSP2-4.5, SSP3-7.0 and SSP5-8.5) compared to the historical period. The correlations behind the Π metric are computed at a 0.01 significance level, and non-significant correlations are shown in white.

L123. "…and the emergence of coupling over some intertropical humid regions, such as Amazonia and…" This is quite confusing to me. The authors say "the emergence of coupling over… Amazonia." However, per panels a-c, there is a strong SM-T coupling in the Amazon. Perhaps the authors mean "the strengthening" instead of "the emergence"?

Response: Thank you for this. While we understand the reviewer, we prefer to keep the word "emergence" because find a shift, in red, from the east to the western humid part which is not water–limiting without the change computed. We have edited to the text to hopefully make it better. It now reads:

All projection scenarios reveal a poleward migration of SM–T coupling regions and the emergence of coupling over some intertropical humid regions, such as western Amazonia and southeast China; Fig. 1d–e (and Fig. S2d–e) illustrate the SM–T coupling differences between the future (2066–2100) and historical period (1981–2014).

L155. Delete one of the dots

Response: Done.

L196. Add a comma before which

Response: Done.

L222. Maybe say "convergence" and "divergence" instead of "ascent" and "descent"

Response: Thank you. We have chosen to use ascent and descent instead in keeping with the language used in the IPCC report.

Figure 4. It is unclear if by "strong warming" the author refers to SSP5-8.5 or to both of the emission scenarios they used. If so, I suggest saying this explicitly, since they showed different SM-T coupling pattern changes depending on the emission scenario. I assume they are referring to SSP5-8.5, but this could confuse some readers.

Response: This refers to SSP5-8.5; we have included some text in the caption of Figure 4 to clarify this. It now reads:

Fig. 4. | Regional divergence of SM–T coupling under global warming. *Warming in the future (under SSP5-8.5) yields distinct SM–T coupling responses. In historical hotspot regions with increased inland moisture transport and reduced subsidence in the future (top figure), precipitation is expected to increase, leading to wetter soil conditions. This reduces the sensitivity of evaporative fraction to soil moisture* (δEF/δθ) *and temperature to EF* (δT/δEF) *resulting in a weakening of SM–T coupling and a steering towards energy-limited conditions. In contrast, regions experiencing a future enhancement of land heating and atmospheric subsidence are expected to witness a heightened evaporation, drier soils and strengthened land–atmosphere feedbacks, reinforcing emergent SM–T coupling (bottom figure).*

L271-272. Again, the author should specify if by "global warming" they mean either SSP5-8.5, SSP1-2.6, or both, as this is confusing. If this is the model to explain the changes in SM-T coupling with SSP5-8.5, what would be the one for SSP1-2.6? Why

Response: We have specified the scenario now. It reads:

Under the high emission warming (SSP5-8.5), regions transitioning from stronger to weaker SM–T coupling (top panel) such as the Sahel and India, exhibit shifts in the large-scale atmospheric circulation, which might draw in more moisture from the ocean and enhanced vertical ascents (decreased subsidence).

L334-335. This is a minor suggestion, but it would be great if the author explain why they used the Priestley and Taylor to calculate Ep.

Response: Thank you. We followed Miralles et al (2012) to calculate Pi. We have added some text to explain this. It reads:

Potential evaporation, λE_p, is estimated based on the Priestley and Taylor (PT) formulation[51], following Miralles, et al.[21]. The PT formulation here uses R_n and T as inputs such that

$$\lambda E_p = \alpha \left(\frac{\Delta}{\Delta+\gamma}\right) R_n - G \qquad (2)$$

References

Miralles, D. G., van den Berg, M. J., Teuling, A. J. & de Jeu, R. A. M. Soil moisture-temperature coupling: A multiscale observational analysis. *Geophysical Research Letters* **39** (2012). https://doi.org/https://doi.org/10.1029/2012GL053703

PRIESTLEY, C. H. B. & TAYLOR, R. J. On the Assessment of Surface Heat Flux and Evaporation Using Large-Scale Parameters. *Monthly Weather Review* **100**, 81-92 (1972). https://doi.org/https://doi.org/10.1175/1520-0493(1972)100

L351. "We follow the approach from previous studies [comma here] which suggested…" or "We follow the approach from previous studies that suggested…"

Response: Done.

L351. To which previous studies the authors are referring here?

Response: We have inserted the relevant references.

L358-359. "The first component can be assumed to be unitless, while the second component is in K." I might be wrong, but is not the opposite here? E.g., δT = $\delta T/\delta EF$, $\delta EF/\delta\theta$

Response: Thank you for pointing this out. The reviewer is right. We have made the corrections. It now reads:

"The first segment is in Kelvin (K), while the second segment can be assumed to be unitless."

We are grateful to the anonymous Reviewer #1 for taking the time to help us improve the quality of the study and manuscript.

Reviewer #2 (Remarks to the Author):

Review of manuscript entitled “Regional bifurcations of soil moisture–air temperature coupling under future climate warming scenarios” by Daniel F.T. Hagan, Guojie Wang, Alan T. Kennedy-Asser, João L. Geirinhas, Kirsten L. Findell, Mingxing Li, Chenxia Zhu, Shijie Li, and Diego G. Miralles
This manuscript presents an important investigation into the projected future changes in global hot-spots of soil moisture–air temperature (SM–T) coupling and the mechanisms driving these changes. Understanding how SM–T coupling will evolve under future climate warming is a critical scientific question, given its implications for heat extremes, land–atmosphere feedbacks, and regional climate impacts. I commend the authors for selecting this timely and highly relevant topic.
The manuscript is thoroughly written, with a clear and well-structured Abstract, Introduction, Results, and Discussion sections. The scientific content is rigorous, the methodology is sound, and the findings provide novel insights into regional bifurcations of SM–T coupling under warming scenarios. The figures are effective in illustrating the key results, and the discussion successfully links the outcomes to broader climate processes.
Overall, the paper makes a valuable contribution to advancing our understanding of land–atmosphere interactions in a changing climate. I find the manuscript to be of high scientific quality and well-suited for publication. Therefore, I strongly recommend acceptance of this manuscript.

Response: We are very grateful to the Reviewer #2 for taking the time to go through the manuscript, and for such encouraging words.

Reviewer #3 (Remarks to the Author):

Overall comments
The paper looks at the impact of climate change on land-atmosphere coupling, which is a major source of uncertainty in 2m-temperature, projections, and the influence of SSP on these changes. It is found that high and low emissions pathways have quite different impacts on the change in SM-T coupling with coupling tending to be enhanced under low emissions pathways, such as SSP-1-2.6. In contrast, a more complex response is seen under high emissions pathways, such as SSP-5-8.5, where coupling is reduced in existing mid-latitude and sub-tropical hotspots due to a reduction in SM (due to an

expanded Hadley circulation and higher precip), but enhanced at higher latitude as a result of soil-moisture drying there.

Overall, I found the results in the paper interesting, the conclusions plausible and a of significance. However, I found there was a general lack of statistical testing, making it hard to completely assess the robustness of the findings. For example, none of the maps of changes under the various emissions pathways give any measure of agreement in the sign of the change or it's statistical significance. As a result, one is left wondering if all the models have the same behaviour, or just one or two. There are also some missing methodological details that would make it hard to reproduce form this manuscript (but which can be easily corrected).

Response: Thank you for these comments. We have updated Fig. 1 to include model agreement for the signs of the changes. Please see the updated figure below. Additionally, we have added included necessary details to improve reproducibility. Please see the details in the responses below.

The paper would also benefit from more careful editorial work to improve the text and make it easier to follow. I provide some examples but don't make a comprehensive list.

Response: Thank you. The authors have gone through to improve the textual integrity of the manuscript.

I wasn't very keen on using the term "bifurcation" to describe different regional responses to climate change or to differences between emissions pathways. It sounds grander than "regional differences" or "differences between emission pathways" but the term has quite a specific definition in dynamical systems theory, and I found it a bit distracting. This terminology may also be physically wrong, for example, it may be that under SSP-5-8.5 the models undergo a strengthening in the existing hotspots in the early 21st century, before this weakening of existing hotspots and northward expansion (seen at the end of century) due to dynamical changes takes over. This would make it more of a transitional state than a bifurcation. This could be checked by looking at the earlier decades of the SSP-5-8.5 projections and would provide an indication of what the early warnings signs are of such a transition.

Response: We are grateful to the reviewer for their insightful assessment. Indeed, we discussed at length about the terminology. Our eventual decision to use 'bifurcation 'highlights two main points: (1) the diverging transitions of the regions to either strengthen or weaken even under the same scenario, and (2) the seemingly contrasting transitions regions undergo due to different warming scenarios, especially in the low-

and high-end emission scenarios used here. We agree with the reviewer that the title might need a change. After a discussion, the authors have decided to change it to

“**Divergent regional responses of soil moisture-air temperature coupling under future climate scenarios**”.

We believe this is a better reflection of the findings and content of the study.

Specific comments
L21 (and elsewhere): “response of coupling to warming” suggest changing to “response of coupling to climate change” or to anthropogenic emissions.

Response: Thank you. We have adapted this suggestion.

L27: is “disproportionate” the right choice of word?

Response: Thank you. Our choice of this word is to highlight where increases in precipitation are outweighed by stronger rises in evaporation, thus, we considered ‘disproportionate’ to be more succinct.

Fig 1: I think it would be useful to indicate some measure of agreement in the ensemble mean changes in Π, and also include a contour of Π in the historical runs in Fig1d and e so that it is easy to see how the changes in Π correspond to the present-day hotspots. Also for Fig S2.

Response: Thank you. We ackowledge the reviewer’s suggestion. We note that in panels a-c from Figure 1 already used stippling to indicate agreement, as indicated by the figure caption: “Dots indicate hotspot regions where at least 8 of the models agree”. We have not added a contour as it was obscured by the stippling to show model agreement. We believe it is more important to highlight the model agreement to opted for stippling instead of the contour. We also agree that a similar scheme should also be included in panel (d) and (e) as well as in Fig. S2. Accordingly new versions of Fig.1 and Fig. S2 were included as below:

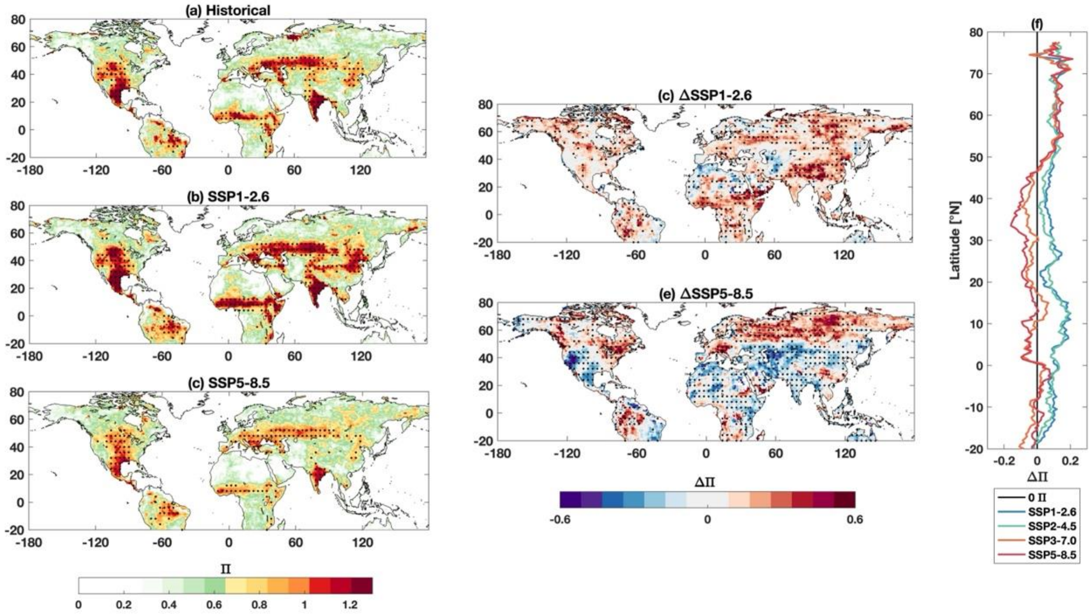

Fig. 1 | SM–T coupling and projected changes in boreal summer. a, SM–T coupling (Π) based on the CMIP6 ensemble mean of the historical period (1981–2014). **b-c**, Same but for future projections (2066–2100) under **b**, SSP1-2.6 and **c**, SSP5-8.5. Dots indicate hotspot regions where at least 8 of the models agree on $\Pi > 0.75$. **d–e**, Change in SM–T coupling (i.e., $\Delta\Pi$) during 2066–2100 relative to 1981–2014 under **d**, SSP1-2.6 and **e**, SSP5-8.5. Dots indicate regions where at least 8 of the models agree on the sign of the change in Π. **f**, Mean latitudinal profile of $\Delta\Pi$ for the future (SSP1-2.6, SSP2-4.5, SSP3-7.0 and SSP5-8.5) compared to the historical period. The correlations behind the Π metric are computed at a 0.01 significance level, and non-significant correlations are shown in white.

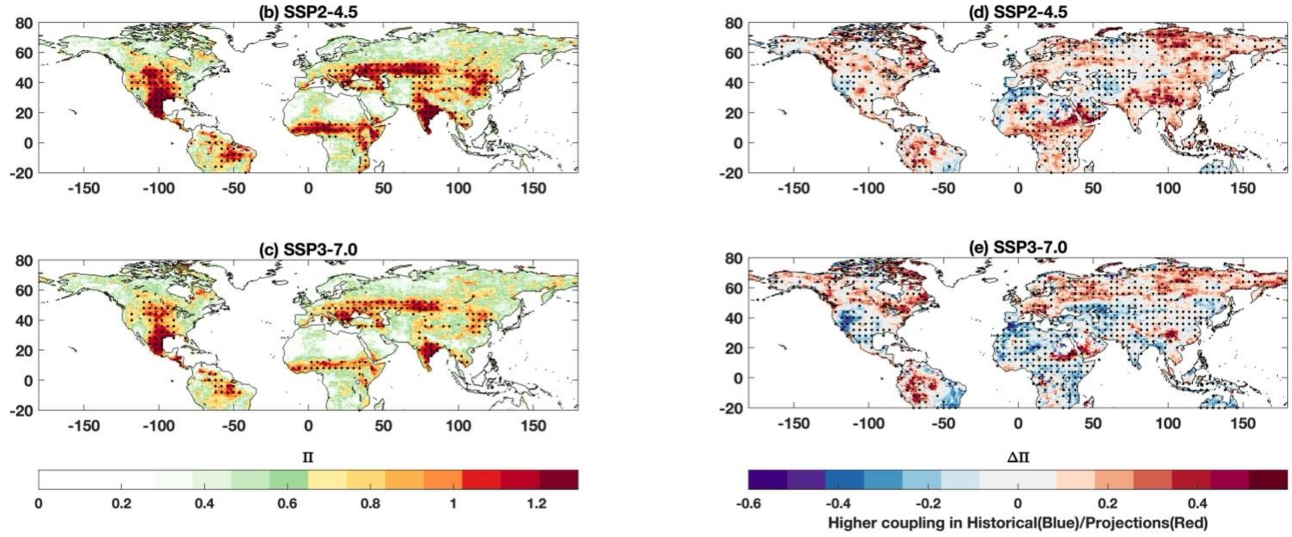

Fig. S2. **The SM–T coupling and projected changes in boreal summer. a**, Expansion (green) and contraction (red) of the historical SM–T coupling hotspots (HS) based on SSP1-2.6 and SSP5-8.5. Common HS refers to hotspots present in both the historical and future (SSP1-2.6 and SSP5-8.5) periods. Ensemble mean of Π from CMIP6 projections (2015–2100) under **b**, SSP2-4.5 and **c**, SSP3-7.0. The dotted regions (i.e., the coupling 'hotspots') in Fig. 1a-e indicate where more than 80% of the models agree on Π >0.7. Change in Π (i.e., $\Delta\Pi$) during the period 2015–2100 under **d**, SSP2-4.5 and **e**, SSP3-7.0 relative to 1981–2014. Dots indicate regions where at least 9 of the models agree on the sign of the change in Π.

L102-110: I found this paragraph a somewhat uncritical appraisal of the CMIP6 ensemble's ability to capture the strength of land-atmosphere coupling. It would be helpful if the colour bar in Fig 1a-c matched with Fig S1, but also useful to include a bias map for the metric in Fig S1, so which highlights the differences. By eye, there seems to be a general tendency to overestimate the coupling strength, compared to Fig S1a as also seen in Hsu and Dirmeyer (2022) for CMIP5 and also Day et al. (2025), for seasonal models. These studies both show that climate models tending to be in a soil-limited evaporation regime over a larger area of the mid-latitudes than the real world. Not having the historical runs in the correct SM-E regime will have some important implications for the ability to predict transitions from one regime to the other, so this caveat needs to be more critically discussed as a limitation of the study.

Response: Thank you for the comment. We consistently inspected our multi-model model results to verify that in each case, at least, 80% of the models were in agreement with respect to mean values and signs (e.g. the revised version of Fig.1 and Fig.S1).

Furthermore, we have added some text to highlight these limitations in the Data and Methods Section, under SM–T coupling. It now reads:

While we consistently inspect agreement in the CMIP6 multi-model analyses (at least, 80% of the models agree), CMIP6 ensembles are known to over-represent soil moisture constraints on evaporation and overestimate, which has implications for the SM–T coupling computations over mid-latitudes[37]*. While our historical evaluation against observations lends confidence, our findings should be interpreted as model-conditioned tendencies rather than absolute magnitudes, acknowledging that consensus can mask the structural biases shared across models.*

References

Hsu, H. & Dirmeyer, P. A. Deconstructing the Soil Moisture–Latent Heat Flux Relationship: The Range of Coupling Regimes Experienced and the Presence of Nonlinearity within the Sensitive Regime. *Journal of Hydrometeorology* **23**, 1041-1057 (2022). https://doi.org/https://doi.org/10.1175/JHM-D-21-0224.1

Fig S2: also need to make colourbars consistent with Fig's 1 and Fig S1.

Response: Thank you. We have set Fig S1a–b colour bars to be consistent with Fig 1. Please see the revised figure below.

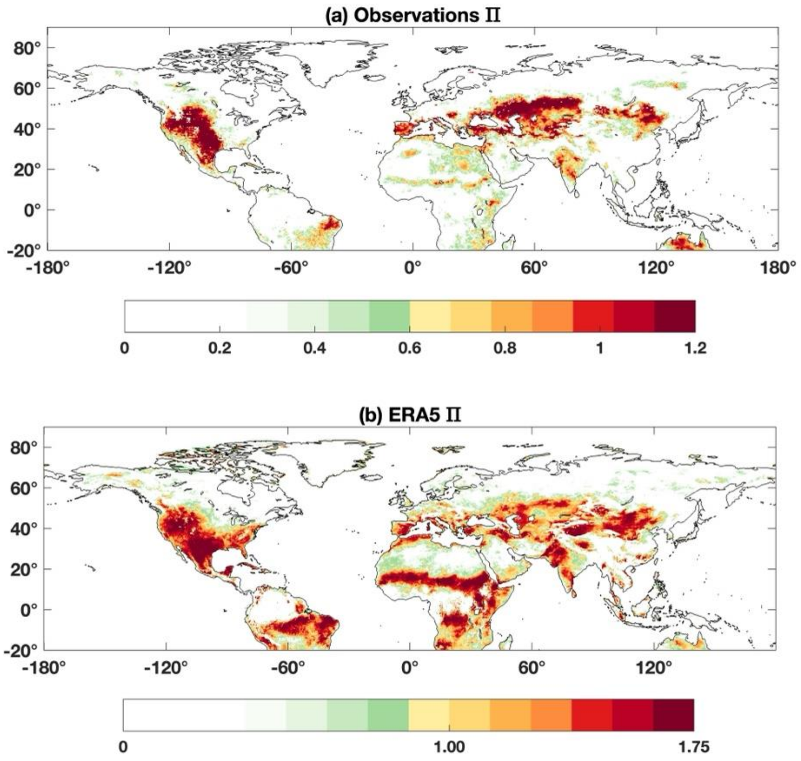

Fig. S1. **Observation- and reanalysis-based boreal summer SM–T coupling.** The SM–T coupling in boreal summer (June, July, August) based on the Π diagnostic calculated using (a) T from CRU and E from GLEAM, and (b) ERA5 reanalysis outputs during the historical period (1981–2014).

L111: "weakening" to "weakened"

Response: Done.

L153: This is very similar to the approach of Dirmeyer (2011) and Dirmeyer et al. (2014) who described a terrestrial and atmospheric leg to the coupling. Suggest citing these papers here and framing in terms of terrestrial-leg and atmospheric-leg.

Response: Indeed, the concept is similar, and while we specifically followed Schwingshackl et al. (2017, 2018) in this case, we agree to the importance of also citing Dirmeyer (2011,2014) where these ideas were earlier introduced. We also decided to use 'segments' in keeping with Dirmeyer (2011). We have added the references, which now reads:

As mentioned above, SM–T coupling is a nonlinear process that can be partitioned into land and atmospheric segments[18,43,48,53].

References

Schwingshackl, C., Hirschi, M. & Seneviratne, S. I. A theoretical approach to assess soil moisture–climate coupling across CMIP5 and GLACE-CMIP5 experiments. *Earth Syst. Dynam.* **9**, 1217-1234 (2018). https://doi.org/10.5194/esd-9-1217-2018

Schwingshackl, C., Hirschi, M. & Seneviratne, S. I. Quantifying Spatiotemporal Variations of Soil Moisture Control on Surface Energy Balance and Near-Surface Air Temperature. *Journal of Climate* **30**, 7105-7124 (2017). https://doi.org/https://doi.org/10.1175/JCLI-D-16-0727.1

Dirmeyer, P. A. The terrestrial segment of soil moisture–climate coupling. *Geophysical Research Letters* **38** (2011). https://doi.org/https://doi.org/10.1029/2011GL048268

L161: Rather than saying "negated" just put a minus in front of the expression.

Response: Done.

Fig2: I think it would be better to use the same colour scheme for the lines in sub panels g and h, since I think they are showing the same variables.

Response: Done. The revised figure is shown below.

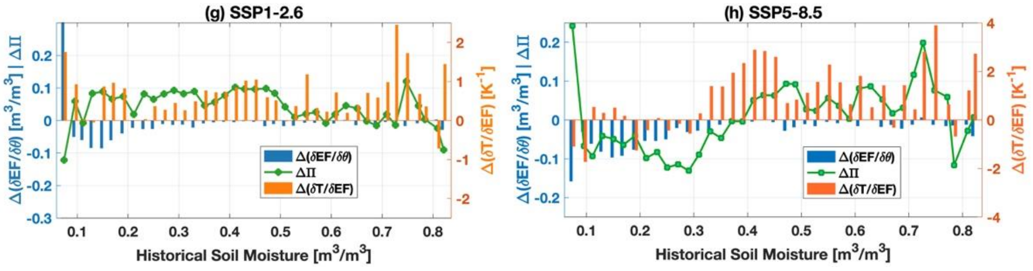

L139-198: I found this section quite hard to follow. It also seems that the metrics used to measure the coupling strength in this section seem not to capture the same changes seen in the previous section with the Π metric (figs 1d & e vs. Fig 2c-f). The section seems to be framed in terms of understanding changes in Π with the two derivatives, but they measure quite different things. As a result, I think this section confuses things rather than explaining them. Then Fig S4 shows something quite similar to the change in Π.

Response: Thank you for this comment. We agree with the reviewer this part could be clearer. The goal here is to unveil whether Π changes come from the and/or atmospheric legs, while using other independent metrics to corroborate the pattern changes in Π. We did not expect a 1:1 alignment, instead we anticipate some explainability between the two, which we have demonstrated both through our results and the texts. Given that this is a nonlinear process, which Pi and the CSM express in linear forms, it makes sense that we do not have a exact representation. Eventually, what we find is that both the partitioned results and the results of the changes in dynamics provide a wholesome explainability (with their individual limitations) to explain the changes in we find in Π. To make our arguments of the partitions stronger, we have added the partitions of the CSM method as well to Fig. S5 which largely validates both the changes in the hotspots and to segments of the coupling. The text in this section has also been updated.

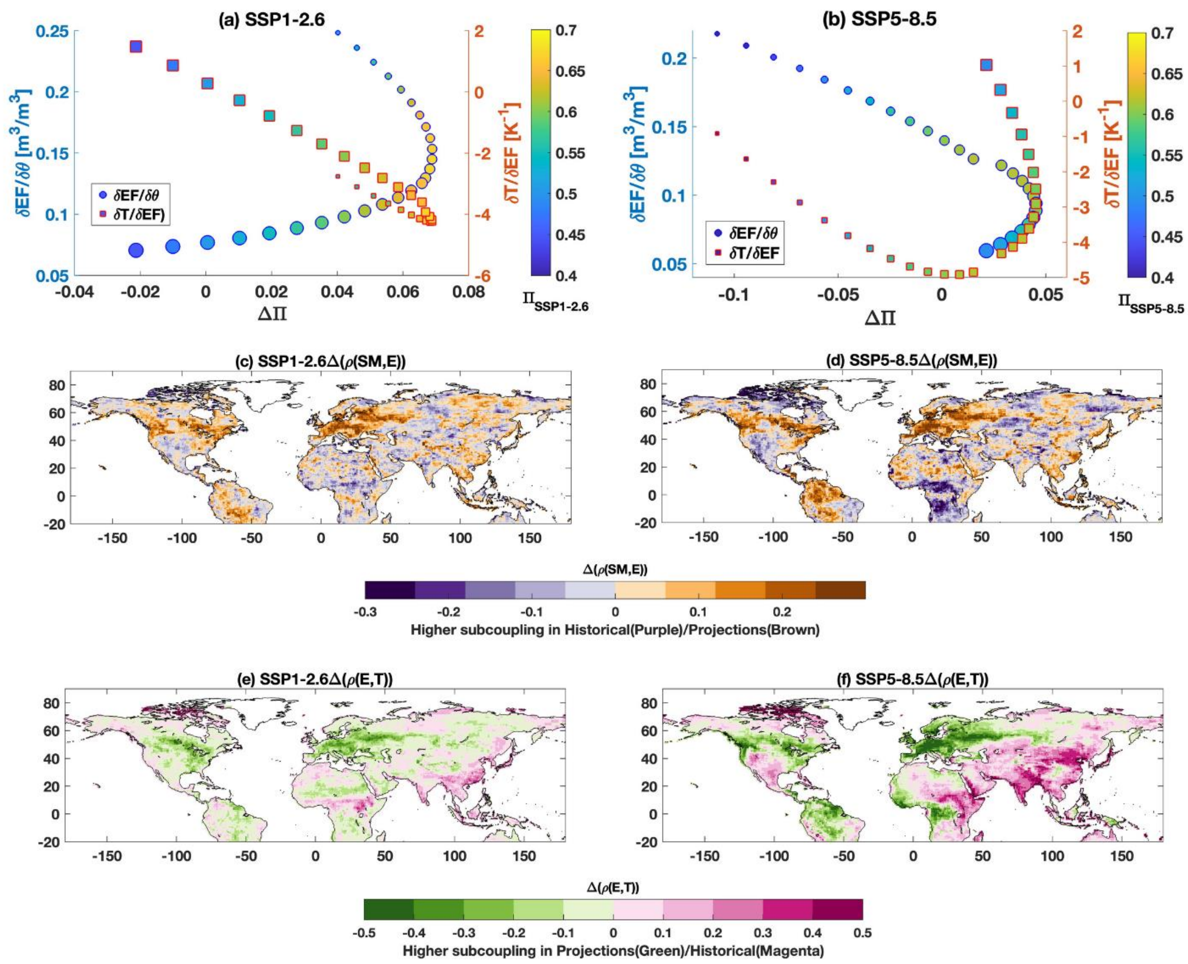

Fig. S5 a, Coupling Partitioning Analysis. Land (left y-axis, circles) and atmospheric (right y-axis, squares) legs of the coupling under SSP1-2.6 binned across historical soil moisture climatology, plotted as a function of change in future SM–T coupling ($\Delta\Pi$). Color shades represent absolute Π. **b**, same as **a**, but SSP5-8.5. The terrestrial leg changes of CSM are shown in **c–d**, and the atmospheric leg is shown in **e–f** for the two scenarios.

Lines 198-199 also read:

Furthermore, the segment-based analysis of CSM (Fig. S5c–f) also align with these findings to explain changes in the SM–T coupling.

L119: It would seem more appropriate to show the agreement in the change in coupling here, instead Fig S3 shows the agreement in the absolute coupling strength.

Response: Thank you for the comment. Indeed, this was a discussion we had during the early versions of the manuscript. At the time we concluded that information

regarding the model agreement in the change of Pi was already being provided in Figure 1 a-c (dots over hotspots). With the inclusion of the same scheme in panels d-e of Figure 1 that show the changes in Pi, we believe that this issue becomes fully and properly addressed.

Fig 3: The maps are quite hard to read, particularly the contours showing the change in Π. I also thought it would be clearer to see where the meridional averages were coming from if the latitude ticks were aligned between a and c and b and d.

Response: Thank you for the comment. We have aligned the meridional averages to the Fig. 3a&b as seen below.

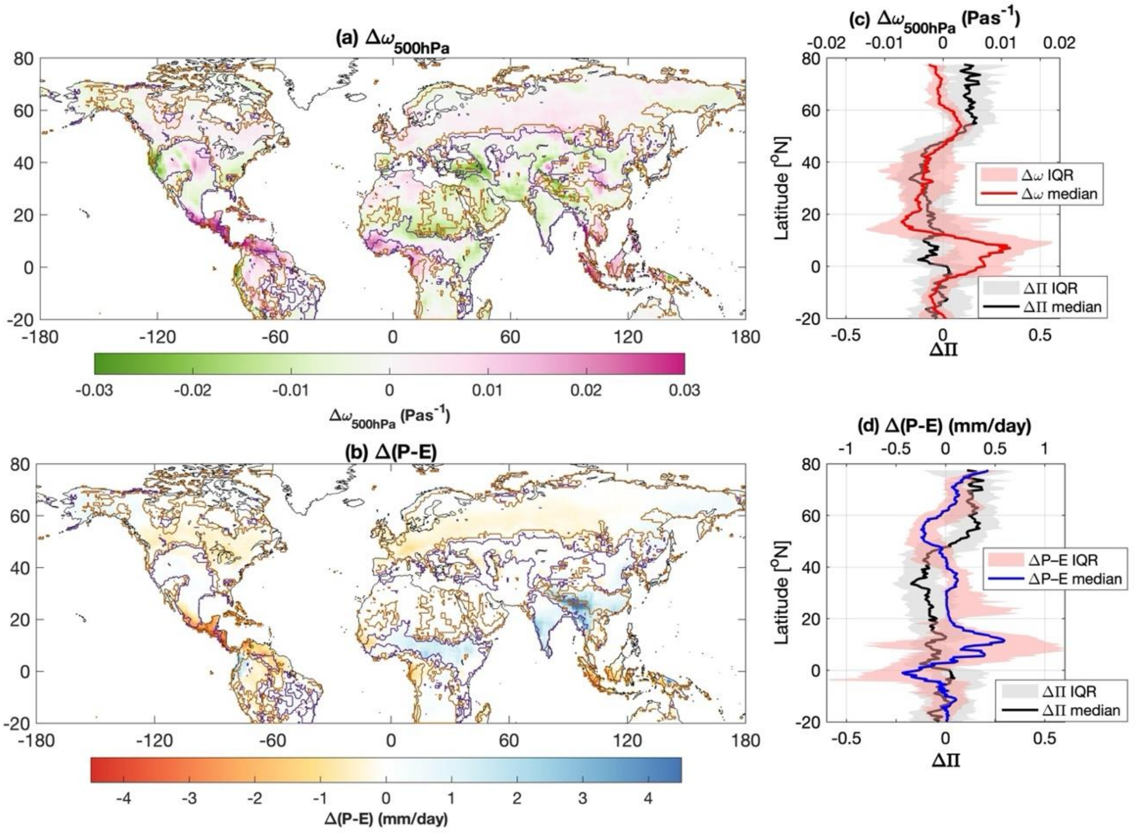

L299-322: It says that monthly data were used for obs but seasonal for CMIP models. Were they averaged to the same frequency before metrics were calculated?

Response: Yes, they were averaged to the same frequency before calculating the metrics. This has been made clearer in the data section.

The Data section reads:

We use seasonal mean net radiation, near-surface mean air temperature, and land surface evaporation to compute the Π coupling diagnostic of SM–T coupling for boreal summer (June, July, and August) at the annual scale.

Under the Observations and reanalysis products, the last line reads:

Parameter data preprocessing steps follow the steps used for the CMIP6 datasets.

L339: It says here that G was calculated as a fixed fraction of the net radiation. I can imagine this has a non-trivial impact when moving from one climate to another. For example if the future climate has lower wind, and reduced turbulent exchange, G will be higher (but not according to this calculation).

Response: We acknowledge the reviewer's concerns . Indeed, there are several uncertainties associated with the computation of potential E (Ep). This was one of the reasons why (1) we checked for consistency, both with GLEAM and ERA5. While we agree with the reviewer about the impact of changes in aerodynamics, we note that the consistency with the CSM, which does not rely on Ep, gives a level of confidence even when we consider the impact of climate change. Nonetheless, there is still more room to explore the impacts of this in future studies. We have made a note in the discussions of the added limitations.

Lines 362-365 now read:

"Furthermore, due to an assumption as the constant value of α within the PT formulation, the impact of climate change in the future SM-T coupling might be biased. Even so, the consistency of future SM-T estimates obtained using both the Π diagnostic and the CSM approach, which does not rely on λE_p, lends a certain level of confidence to our results."

It should also be mentioned in the data section what depth of soil-moisture was used from CMIP and ERA5/GLEAM.

Response: Thank you. Soil moisture is not used for ERA5 and GLEAM because Pi does not require this variable. For CMIP6, we used the *mrso* variable, which represents the *total soil moisture content*. We have clarified this in the data section.

Lines 308-309 now read:

We also use latent and sensible heat fluxes to compute the evaporative fraction, and total soil moisture content.

We are grateful to the anonymous Reviewer #3 for taking the time to help us improve the quality of the study and manuscript.

Dear Editor and Reviewer,

Thank you for the extended comments to clarify crucial points in our study and write up. We have responded in **purple** to all the comments raised by the reviewer in this second round (in ***green***) to make the flow of the conversation clearer. Revisions made from this round are ***italicized in purple*** in this response document and highlighted in the main manuscript.

Reviewer #3 (Remarks to the Author):

Round 2 comment: *I would like to thank the authors for their work to improve the manuscript. However, I still believe some additional changes are required before the manuscript is ready for publication. I found the section on land and atmosphere segments difficult to read, and the metric chosen in conflict with those in the supplementary material (Fig S5). I also found the conclusion that there is a fundamental difference in the response to low SSP and high SSP not well justified. This is because although the response in the Pi metric in Fig 1 shows a difference between the SSPs, the other metrics shown in Figs S4 and S5 show much more similarity between SSPs, with the SSP126 just showing a weaker response than SSP585 with a broadly similar pattern suggesting the Pi metric is really an outlier here.*

Response: We thank the reviewer for raising these helpful comments. We have revised the land and atmosphere segments section to (a) better justify the selection of Π as core metric, (b) explain that the study of $\delta EF/\delta SM$ and $\delta T/\delta EF$ is merely done to attribute changes in coupling to each of the two coupling legs, (c) discuss where changes in those legs can explain the observed changes in Π, and (d) clarify the scenario-dependent contrasts that are displayed. Detailed responses to these comments can be found below.

Round 2 comment: *It occurred to me when performing this second review that the choice of metrics is not really discussed. Some metrics are shown in the main paper, others shown in the supplement but without any justification for the choices of what metric is used where. The paper by Lorenz et al. (2015) might be useful to reference here as it includes a discussion about the impact the choice of metric has on estimates on the change in SM-atmosphere coupling.*

Response: We appreciate the reviewer for the thoughtful comments. We have now expanded the justification for using Π as the primary diagnostic and clarified the complementary roles of and the land – atmosphere segment partitioning ($\delta EF/\delta SM$, $\delta T/\delta EF$).

Moreover, the critical soil moisture (CSM) metric is no longer shown for comparison. Unlike Π, the CSM metric aims to separate water-limited from energy-limited conditions, rather than capturing the influence of SM on temperature, since the influence of radiation on the variability in both temperature and soil moisture and their correlation is not directly accounted for in this

metric. Therefore, only in cases in which SM–temperature coupling (Π) changes reveal changes in water limitation (instead of for instance changes in heat advection) the CSM metric will respond in a manner that is closely aligned with Π. Since both metrics are not showing the same sensitivities, we believe that including both is counterproductive, as hinted by the reviewer's comments. Regarding the close correspondence between CSM and the δEF/δSM and δT/δEF patterns (Fig. 2), this correspondence relates to the fact that the CSM metric is computed based on the correlation of E to SM and T, and thus explicitly reflects the covariance between these variables, while Π follows a process-based rationale to isolate the influence of other variables (like radiation) on these covariances.

Our rationale for selecting Π as core coupling metric is now also properly justified in the paper. The goal of Π is to quantify the improvement at explaining near-surface air temperature variability when using actual sensible heat (H) relative to using potential sensible heat (Hp) in the surface energy balance. The metric embeds a physical counterfactual experiment, enabling Π to directly isolate the moisture constraint on surface energy partitioning and temperature. As warming alters evaporative demand and shifts regions across wet, transitional, and dry soil-moisture regimes, the gap between H and Hp changes. Moreover, the influence of the local energy balance on local temperature may be shaped by changes in advection and cloudiness, affecting the correlations between temperature and the output from either of the two surface energy balances (i.e. H or Hp). Π was developed to detect exactly this type of regime-dependent response and isolate the influence of soil moisture regardless of other air temperature drivers. These characteristics make Π a great fit for studying scenario-based future SM–T coupling study in a non-stationary climate, while similar correlation-based diagnostics lack this counterfactual baseline.

Nonetheless, the metric also requires assumptions on linearity given the use of correlations and cannot be used to detect the direction of causality. More importantly, it does not enable the direct decomposition of the coupling signal into land and atmospheric segments (in the strictest sense), justifying the consideration of δEF/δSM and δT/δEF for this partitioning.

The following are revisions we have made in the manuscript to justify our choice of all the approaches used.

For Π (Lines 97–104)

To explore SM–T coupling, we employ the Π diagnostic, which quantifies the influence of SM on T based on the differential skill of actual and potential evaporation to explain T anomalies[21]. This metric leverages the additional explanatory power of sensible heat flux on temperature relative to a hypothetical surface without moisture constraints, while isolating it from the influence of

fluctuations in net radiation. As such, Π captures the influence of long-term changes in water limitation as well as trends in cloudiness and advection[7,21]. Π is a dimensionless quantity, with higher positive values indicating a stronger influence of SM on T. It indirectly embeds the two segments of the coupling between SM and T: the SM constraint on the surface energy balance partitioning and the subsequent response of air temperature.

SM–T partitioning (Lines 157–169)

As mentioned above, SM–T coupling is a nonlinear process that can be partitioned into land and atmospheric segments[18,43,49,55]. Both segments are expected to change under global warming, reflecting the influence of thermodynamic and dynamical perturbations on SM–T coupling. Here, we untangle these two segments of SM–T coupling to unravel the reasons for the changes in the coupling hotspot regions shown in Fig. 1. To do this, we select a metric that explicitly separates the coupling between land and atmosphere segments. The land segment is evaluated by exploring the sensitivity of Evaporative Fraction (EF, the ratio between latent heat over net radiation) to changes in SM (i.e., δEF/δSM), and the atmospheric segment is assessed by exploring the sensitivity of air temperature to changes in EF (i.e., δT/δEF, expressed as negative for visual consistency)[43,49]. In nature, strong SM–T coupling is expected where both sensitivities are favourable, capped by physical limitations in SM and net radiation[12]. Furthermore, given that the two segments are hinged on the EF, changes in the EF, and shifts in regions where it is limited either by SM or net radiation, determine how the coupling would evolve in the future (Fig. 2a–b). This complements the more process-based Π metric while enabling the direct decomposition of coupling into its two segments.

SM–T Partitioning (Methods), Lines 383-387:

Unlike correlation-based metrics which may respond linearly to background warming, obscuring regime-dependent nonlinearities that dominate land–atmosphere interactions, this approach explicitly computes the sensitivity of EF to SM changes across dry, transitional and wet regimes[18,43,48]. Thus, it provides a more physically nuanced view of the terrestrial segment of the SM–T coupling under climate change.

Round 1 Comment

Round 1 Comment: I wasn't very keen on using the term "bifurcation" to describe different regional responses to climate change or to differences between emissions pathways. It sounds grander than "regional differences" or "differences between emission pathways" but the term has quite a specific definition in dynamical systems theory, and I found it a bit distracting. This terminology may also be physically wrong, for example, it

may be that under SSP-5-8.5 the models undergo a strengthening in the existing hotspots in the early 21st century, before this weakening of existing hotspots and northward expansion (seen at the end of century) due to dynamical changes takes over. This would make it more of a transitional state than a bifurcation. This could be checked by looking at the earlier decades of the SSP-5-8.5 projections and would provide an indication of what the early warnings signs are of such a transition.

Response: We are grateful to the reviewer for their insightful assessment. Indeed, we discussed at length about the terminology. Our eventual decision to use 'bifurcation 'highlights two main points: (1) the diverging transitions of the regions to either strengthen or weaken even under the same scenario, and (2) the seemingly contrasting transitions regions undergo due to different warming scenarios, especially in the low- and high-end emission scenarios used here. We agree with the reviewer that the title might need a change. After a discussion, the authors have decided to change it to

"**Divergent regional responses of soil moisture-air temperature coupling under future climate scenarios**".

We believe this is a better reflection of the findings and content of the study.

Round 2 comment: *The response here missed my second point about the possibility that the enhanced midlatitude coupling being a transition state.*

Response: We apologise for missing that point. Indeed, this was a concern for the authors at the initial stages of the study, and it motivated an analysis of a 20-year running window of Π estimates from 2015 to 2100. The results showed that these changes occurred well within the first 40 years of the period and stabilized all the way to the end of the century, implying that it is not a transition state. As a result, the authors chose to mainly focus on the last 4 decades of the century. These were performed both on CMIP5 and CMIP6 datasets with the latter showing more levelled changes by the end of the century.

We now mentioned this under the Data and Methods section (CMIP6 projections). Lines 310–312 now read:

The future period (2066–2100) is chosen because Π is stable during this timeframe in the scenarios after a period of transitioning.

Round 1 comment: Fig 1: I think it would be useful to indicate some measure of agreement in the ensemble mean changes in Π, and also include a contour of Π in the historical runs in Fig1d and e so that it is easy to see how the changes in Π correspond to the present-day hotspots. Also for Fig S2.

Round 1 Response: Thank you. We ackowledge the reviewer's suggestion. We note that in panels a-c from Figure 1 already used stippling to indicate agreement, as indicated by the figure caption: "Dots indicate hotspot regions where at least 8 of the models agree". We have not added a contour as it was obscured by the stippling to show model agreement. We believe it is more important to highlight the model agreement to opted for stippling instead of the contour. We also agree that a similar scheme should also be included in panel (d) and (e) as well as in Fig. S2. Accordingly new versions of Fig.1 and Fig. S2 were included as below:

Round 2 comment: *Suggest stating the percentage of models agreeing on the sign of the response as a percentage as well, so the casual reader doesn't have to search for the number of models used.*

Round 2 Response: Thank you for the suggestion. We have revised them accordingly for Fig. 1 and Fig S2 (see below).

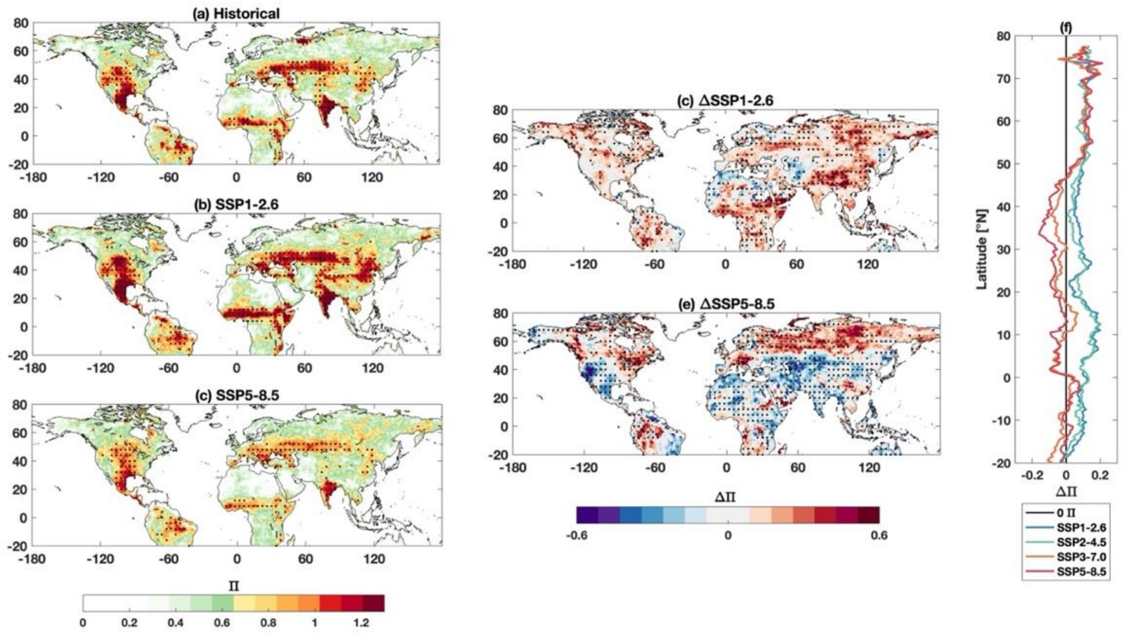

***Fig. 1 | SM–T coupling and projected changes in boreal summer. a**, SM–T coupling (Π) based on the CMIP6 ensemble mean of the historical period (1981–2014). **b-c**, Same but for future projections (2066–2100) under **b**, SSP1-2.6 and **c**, SSP5-8.5. Dots indicate hotspot regions where at least 70% of the models agree on Π > 0.75. **d–e**, Change in SM–T coupling (i.e., ΔΠ) during 2066–2100 relative to 1981–2014 under **d**, SSP1-2.6 and **e**, SSP5-8.5. Dots indicate regions where at least 70% of the models agree on the sign of the change in Π. **f**, Mean latitudinal profile of ΔΠ for the future (SSP1-2.6, SSP2-4.5, SSP3-7.0 and SSP5-8.5) compared to the historical period. The*

correlations behind the Π metric are computed at a 0.01 significance level, and non-significant correlations are shown in white.

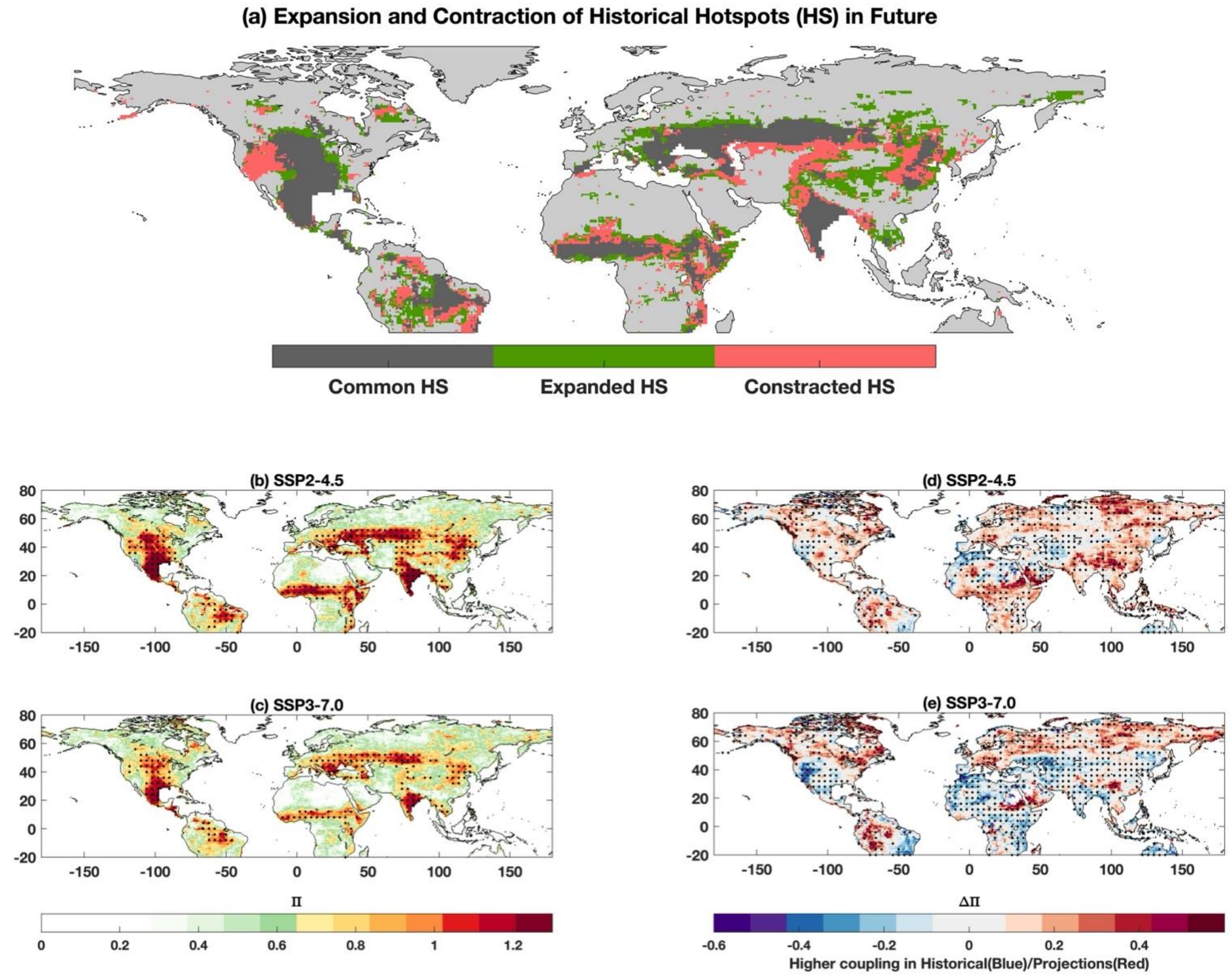

***Fig. S2 | The SM–T coupling and projected changes in boreal summer. a**, Expansion (green) and contraction (red) of the historical SM–T coupling hotspots (HS) based on SSP1-2.6 and SSP5-8.5. Common HS refers to hotspots present in both the historical and future (SSP1-2.6 and SSP5-8.5) periods. Ensemble mean of Π from CMIP6 projections (2015–2100) under **b**, SSP2-4.5 and **c**, SSP3-7.0. The dotted regions (i.e., the coupling 'hotspots') in Fig. 1a-e indicate where more than 70% of the models agree on Π >0.7. Change in Π (i.e., $\Delta\Pi$) during the period 2015–2100 under **d**, SSP2-4.5 and **e**, SSP3-7.0 relative to 1981–2014. Dots indicate regions where at least 70% of the models agree on the sign of the change in Π.*

Round 1 comment: Fig S2: also need to make colourbars consistent with Fig's 1 and Fig S1.

Response: Thank you. We have set Fig S1a–b colour bars to be consistent with Fig 1.

Round 2 comment: *Please also make the fig S1b colourbar consistent.*

Round 2 Response: Thank you for the suggestion. We have revised them accordingly as show in Fig. S1.

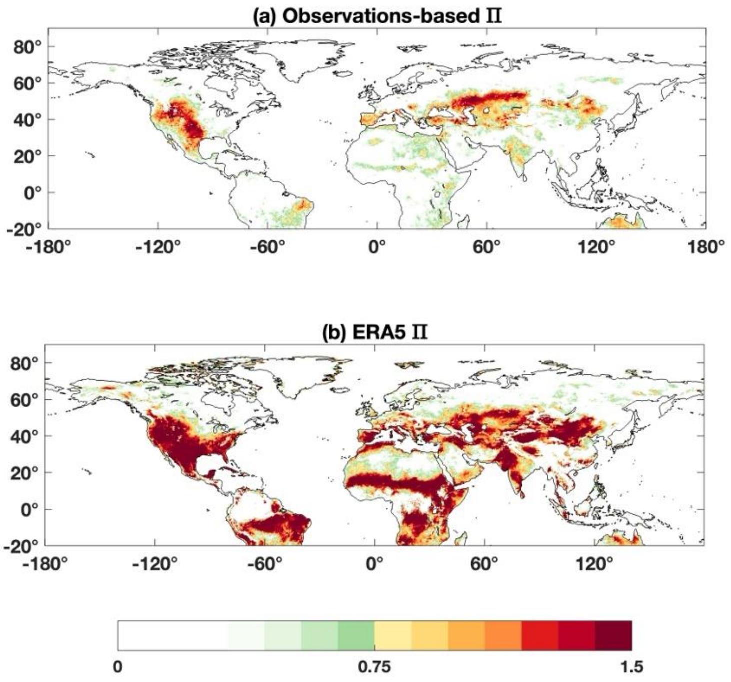

Fig. S1 | Observation- and reanalysis-based boreal summer SM–T coupling. *The SM–T coupling in boreal summer (June, July, August) based on the Π diagnostic calculated using (a) CRU T and GLEAM E, and (b) ERA5 T and E during the historical period (1981–2014).*

Round 1 comment: L161: Rather than saying "negated" just put a minus in front of the expression.

Response: Done.

Round 2 comment: *Also change associated figure caption*

Response: Thank you. The caption of Fig. 2 has been revised. It now reads:

***Fig. 2 | Changes in the projected land and atmosphere segments of SM–T coupling. a**, Idealized evaporative fraction (EF)–soil moisture (SM) relationship. **b**, Π as a function of historical SM climatology for the historical period, SSP1-2.6 and SSP5-8.5 scenarios. **c**, Changes in the land segments of the coupling, expressed as the EF sensitivity to SM changes (ΔδEF/δSM, m^3m^{-3}), for the period 2066–2100 relative to 1981–2014 under SSP1-2.6. **d**, same as **c**, but for SSP5-8.5. **e**, Changes in the atmospheric segments of the coupling, expressed as T sensitivity to EF changes (ΔδT/δEF, K), for the period 2066–2100 relative to 1981–2014 under SSP1-2.6. **f**, same as **e**, but for SSP5-8.5. -δT/δEF is used here for visual consistency. **g**, Land (left y-axis) and atmospheric (right y-axis) segments of the coupling under SSP1-2.6 binned across historical soil moisture climatology. Line plots show change in future SM–T coupling (ΔΠ). **h**, same as **g**, but SSP5-8.5. The spread in **b** shows the standard deviation of the 11 climate models as an estimate of model uncertainty. Dots indicate regions where at least 8 of the models agree on the sign of the change in the segments. All the segment computations are done at a 0.01 significance level based on a z-test.*

Round 1 comment: L139-198: I found this section quite hard to follow. It also seems that the metrics used to measure the coupling strength in this section seem not to capture the same changes seen in the previous section with the Π metric (figs 1d & e vs. Fig 2c-f). The section seems to be framed in terms of understanding changes in Π with the two derivatives, but they measure quite different things. As a result, I think this section confuses things rather than explaining them. Then Fig S4 shows something quite similar to the change in Π.

Round 1 Response: Thank you for this comment. We agree with the reviewer this part could be clearer. The goal here is to unveil whether Π changes come from the and/or atmospheric legs, while using other independent metrics to corroborate the pattern changes in Π. We did not expect a 1:1 alignment, instead we anticipate some explainability between the two, which we have demonstrated both through our results and the texts. Given that this is a nonlinear process, which Pi and the CSM express in linear forms, it makes sense that we do not have a exact representation. Eventually, what we find is that both the partitioned results and the results of the changes in dynamics provide a wholesome explainability (with their individual limitations) to explain the changes in we find in Π. To make our arguments of the partitions stronger, we have added the partitions

of the CSM method as well to Fig. S5 which largely validates both the changes in the hotspots and to segments of the coupling. The text in this section has also been updated.

Lines 198-199 also read:

Furthermore, the segment-based analysis of CSM (Fig. S5c–f) also align with these findings to explain changes in the SM–T coupling.

Round 2 comment: *Unfortunately, I still found this section very difficult to follow. I understand that the changes seen in the linear segment analysis in Fig 2 will not match the changes in Π (Fig 1) perfectly,*
but they don't look to me like they match what is seen in Fig 1 at all. In contrast the change in correlation metrics shown in Fig S5d & f look to capture a similar pattern to Fig 1e quite clearly, i.e. the dipoles in Π North America and Eurasia. As a result, I think the section on the land and atmosphere, which is supposed to elucidate the mechanisms, actually confuses things greatly. For example, in fig 1e there is a clear latitudinal dependence to the change in coupling strength, with the northern mid-latitudes reducing and the higher latitudes increasing. I can't see this basic pattern in Fig 2. It is also not clear to me why the changes in this metric are expressed as a function of the SM in the historical run. It is not clear to me what insight this gives compared to plotting as a function of latitude, which would make the comparison with Fig 1 easier to see.
After having a closer look at Fig S5, I am also slightly concerned that the papers main conclusion (RE divergent responses to different scenarios) may be quite metric dependent. This is because the correlation metrics used in Fig S5 show much more similarity between the response under the SSP1-2.6 scenario and the SSP5-8.5 scenario than in Fig 1. The change in both metrics have a dipole over Eurasia and north America.

Lorenz, R., A. J. Pitman, A. L. Hirsch, and J. Srbinovsky, 2015: Intraseasonal versus Interannual Measures of Land–Atmosphere Coupling Strength in a Global Climate Model: GLACE-1 versus GLACE-CMIP5 Experiments in ACCESS1.3b. Journal of Hydrometeorology, 16, 2276–2295, https://doi.org/10.1175/JHM-D-14-0206.1.

Round 2 Response: We are grateful to the reviewer for the careful read and thoughtful comments. We acknowledge how the points raised may appear confusing and have revised the manuscript to clarify three main aspects: (i) why Π and the partitioning metric are not expected to show identical spatial patterns, (ii) why the partitioning is analysed as a function of historical soil moisture, and (iii) why scenario-based differences are not merely a metric artefact. Below, we first explain the conceptual distinctions, and then summarise the revisions implemented in the manuscript.

(i) Both Π and the partitioning by Schwingshakl et al. The partitioning separates the SM–T coupling chain **in complementary but physically distinct ways** because they

emphasize different physical quantities. Π measures how strongly soil moisture limitations alter the contrast between actual and potential evaporation, and how this altered flux partitioning influences temperature. Thus, its sensitivity stems from changes in the gap between H and Hp and from shifts in the H–T relationship. The partitioning quantifies the sensitivity of evaporative fraction to soil moisture ($\delta EF/\delta\theta$) and then evaluates how these EF perturbations influence temperature. It is therefore more directly sensitive to soil-moisture regime occupancy, whereas Π responds most strongly when SM deficits affect the energy balance (H-Hp). This implies that while their climatological patterns would be similar, the overall regional patterns of future changes may not completely align. Nonetheless, since they also highlight similar changes in relevant regimes, the partitioning can be used to provide a first-order SM driven interpretation of the changes in Π. In the manuscript, we have discussed Fig. 2 to do this, along with revisions to clarify these.

The discussion on Fig. 2 has also been revised where we mainly focus on the changes in the historical hotspots and main emerging regions. They revisions in Lines 170-203 read as follows:

Overall, the patterns in Fig. 2c–f help attribute the changes in the historical hotspot regions in Fig. 1d–e. Figure 2c–d indicate a future weakening of δEF/δSM in the historical transitional regions associated with hotspots, particularly for SSP5-8.5, and an increase in higher-latitude humid regions like Western Europe, the Amazon basin and North America. The latter indicates a likely shift towards sub-humid conditions and, thus, a more responsive relationship between EF and SM (Fig. 2a). This is consistent with recent findings pointing to the expansion of sub-humid climate zones at high latitudes[30,56]. Meanwhile, there is a widespread increase in the atmospheric segment (i.e., δT/δEF) in SSP1-2.6 projections (Fig. 2e), as seen in Π (Fig. 1b, 2b). in high latitudes and tropical regions, where SM–T coupling is expected to increase. This increase in δT/δEF indicates a higher sensitivity of near surface air temperature to changes in the local energy balance and lower sensitivity to other dynamical processes such as advection and subsidence. However, the changes in the δT/δEF of SSP5-8.5 are more localised with stronger gradients, explaining why the SSP5-8.5 Π is not increasing as widely, but mostly localised in the noted humid regions such as Europe, the Amazon basin, Southeast China (Fig. 2f). On the other hand, historical transitional regions such as Central China (Huabei) and India show δT/δEF decreases under SSP5-8.5 (Fig. 2f). Figure 2f also shows drying patterns in western Sahel (increased δT/δEF), and moistening patterns towards the east (weakened δT/δEF)[38] in SSP5-8.5. The westward shift in the emerging regions in the SSP5-8.5 Π is also well explained by increased sensitivities in δEF/δSM and δT/δEF, epsecially across Europe, the Amazon basin and China. Overall, regions of increasing SM–T coupling appear to be driven more by changes in the atmospheric segments, with changes in the land segment mainly driving weakening trends.

We further examine future $\Delta\delta EF/\delta SM$ and $\Delta\delta T/\delta EF$ binned across historical SM climatology, linked to changes in the future SM–T coupling ($\Delta\Pi$, green lines in Fig. 2g–h) for SSP1-2.6 and SSP5-8.5 (Fig. 2g–h, Fig. S5a–b). Overall, Figure 2g–h shows that future change in the SM–T coupling is shaped by a tug-of-war between the changes in the land and atmospheric segments capped by SM conditions (Fig. S5a–b). Under SSP1-2.6, moderate changes in the segments, especially $\Delta\delta T/\delta EF$ lead to stronger SM–T coupling ($+\Delta\Pi$), mostly over the intermediate soil moisture conditions (Fig. 2g). On the other, larger $\Delta\delta T/\delta EF$ variations under SSP5-8.5 appear to influence more nuanced changes in the SM–T coupling characterized by reduced coupling in some intermediate SM conditions (transition regions) where atmospheric drying reduces, and emerging stronger coupling in the sub-humid and humid SM conditions (Fig. 2h, Fig. S5b). Both scenarios indicate that the emergence of SM–T coupling can be linked to stronger drying patterns in $\Delta\delta T/\delta EF$ capped by $\Delta\delta EF/\delta SM$ changes as a response to different degrees of warming. More importantly, increases in the coupling appear to fall within intermediate conditions of both the land and atmospheric segments (Fig. S5), highlighting the nonlinear nature of the coupling. Thus, patterns in both segments align closely with the simulated shifts in total SM–T coupling (Fig. 1), which highlight their crucial role in shaping future hotspot patterns under global warming.

In the Methods section for the SM–T Partitioning, Lines 388-390 now read:

Nonetheless, differences between the changes obtained from this partitioning and those in $\Delta\Pi$ are expected, as the former is intrinsically more responsive to changes in soil-moisture regime occupancy, while Π reflects shifts in the actual–versus–potential evaporation contrast.

(ii) The EF–SM relationship is fundamentally SM-regime dependent. Furthermore, historical SM regimes also correspond to the well-established historical SM–T coupling hotspots, making them meaningful references for analysing how the coupling evolves. Moreover, Pi shifts are not purely latitudinal; they also reflect zonal, as noted in Fig. 1d–e, although the former is more dominant. Plotting Fig. 2 against latitude would therefore obscure important changes in newly emerging transitional regions. This is now explained in both the Methods and the Results sections.

To highlight why historical SM is used in Fig. 2, we have revised the text.

Lines 167-168 now read:

We analyse the coupling and the segments across historical SM climatology since patterns of strong SM–T couplings are well known across these SM regimes[3,43].

From points (i) and (ii), it follows that the partitioning does not reproduce the high-latitude latitudinal dipole in ΔΠ to the same degree as Π or CSM because partitioning bins responses by soil-moisture regime, making it more responsive to projected shifts in SM trends, which are rather nuanced. In contrast, both Π and CSM respond strongly to thermodynamic intensification of evaporative demand: Π captures this explicitly through the H–Hp contrast, while CSM reflects it indirectly through shifts in the balance between energy- and moisture-controlled evaporation. Even so, the partitioning still captures the key regional and regime-specific changes in evaporative sensitivity and atmospheric response. These mechanistic shifts align with—and help explain—the spatial redistribution of SM–T coupling seen in ΔΠ. This is why we use the partitioning to interpret why the Π patterns emerge, even if the precise spatial gradients differ.

(iii) Regarding the concern that scenario-dependent differences may be metric dependent:

We agree that correlation-based metrics in Fig. S5 show greater similarity between SSP1-2.6 and SSP5-8.5 than Π does. We now clarify why this behaviour is expected. Correlation diagnostics blend soil-moisture-driven and radiation-driven co-variability and therefore respond largely linearly to background warming. As a result, they smooth over the nonlinear regime transitions that occur when regions shift between energy-limited and moisture-limited evaporative states. Π, by contrast, is constructed to detect departures from an energy-limited reference state by contrasting actual and potential evaporation. It is therefore inherently more sensitive to scenario-dependent thermodynamic intensification of evaporative demand.

The CSM metric, which also evaluates soil-moisture control relative to an energy-limited baseline, exhibits scenario contrasts consistent with Π (Fig. S4b–c). This independent agreement confirms that the SSP divergence is not entirely an artefact of Π, but reflects physically coherent changes in the balance between energy and moisture control on E.

Once again, we are very grateful for the reviewer’s careful engagement.

Response to Reviewer

We are grateful to the editor and reviewer for helping us bring our manuscript to this point. In this round, the reviewer's comments are left in black text and our response is provided in blue, and changes in the manuscript are written in blue.

Comment: I would like to thank the authors for the changes to the text and figures, but in my view the conclusions are still not completely well justified from the analysis.

The main outstanding issue for me is that the section on land and atmosphere segments is still hard to follow, and in particular it is hard to both understand Fig 2 and reconcile it with results seen in Fig 1 and in the summary of processes seen in the schematic in Fig 4. This has not really changes though the previous iterations of the manuscript.

Response: We appreciate the reviewer for carefully going through our previous response and the manuscript to point out further inconsistencies. Below, we provide more details of the changes we have made following the reviewer's suggestions.

Comment: In this version it is also still not clear why the coupling metrics are plotted against soil-moisture in the historical runs in Figs 2b,g and h. The explanation in the text: "We analyse both segments across historical SM climatology since patterns of strong SM–T couplings are well known across these SM regimes" is not really illuminating about the motivation of presenting it like this or a sufficient explanation. In what way are they 'well known' in? The authors provided detailed explanation of the metrics in the last response to my comments, but the important thing to explain is how plotting them like this (binning with soil moisture across models and regions) informs the physical interpretation of change in coupling regimes. Whether a location is in an energy or soil limited ET regime will depend not just on the soil moisture, but also the PWP and critical values of soil moisture. As a result grouping it like this is not really grouping by ET regime, so why is it shown like this? If this has been done elsewhere and gives some particular insight then say so and explain what it is. Otherwise, please consider presenting in another way, such as binning by latitude to enhance interpretation of the northward shift in coupling zones.

Response: We appreciate the reviewer's helpful suggestions, and we agree that the suggestion provided would help improve the consistency of our findings. We have now replaced the soil moisture binned analyses with latitudinal/zonal analysis to make the flow of the story more coherent. In the revised version, we have included zonal variations of the partitioned analysis to highlight how it relates to the SM–T coupling. This is now represented in Fig. 2e–f as shown below. Additionally, we have streamlined and sharpened most of the text for Fig. 2 to make the interpretation clearer and better highlight our analyses. Please see the details at the end of the comments.

Comment: In my view it would be much easier to conclude something from this section if there was some statistical analysis to link these metrics with the changes in hotspot regions. For example what percentage of the weakening (and emerging) hotspots shown in the Fig 4 insert have weakening or strengthening del EF/delSM or delT/delEF. I think this type of contingency table would help to show that this analysis is informative. At the moment I am still not convinced that it is.

It is really not clear without this type of statistical analysis that the neat summary that the clear summary regarding the role of land and atmosphere coupling segments in the regime transitions shown in Fig 4 is supported by the evidence in this section. I don't think the argument that it is non-linear and we wouldn't expect 1-1 agreement is consistent with this clear picture and if things are more complicated, this needs to be stated clearly and understood through additional analysis before it is published.

Response: Thank you again for these helpful insights and suggestions. Following the suggestions, we have quantified the association between Δ(δEF/δSM) and Δ(δT/δEF) and regions where Pi strengthens and weakens, indicated in Fig. 2e–f. Below, we show the full changes in Fig. 2 and changes made in the manuscript on Fig. 2a–h. Edits have also been propagated into Fig. 4 and its text.

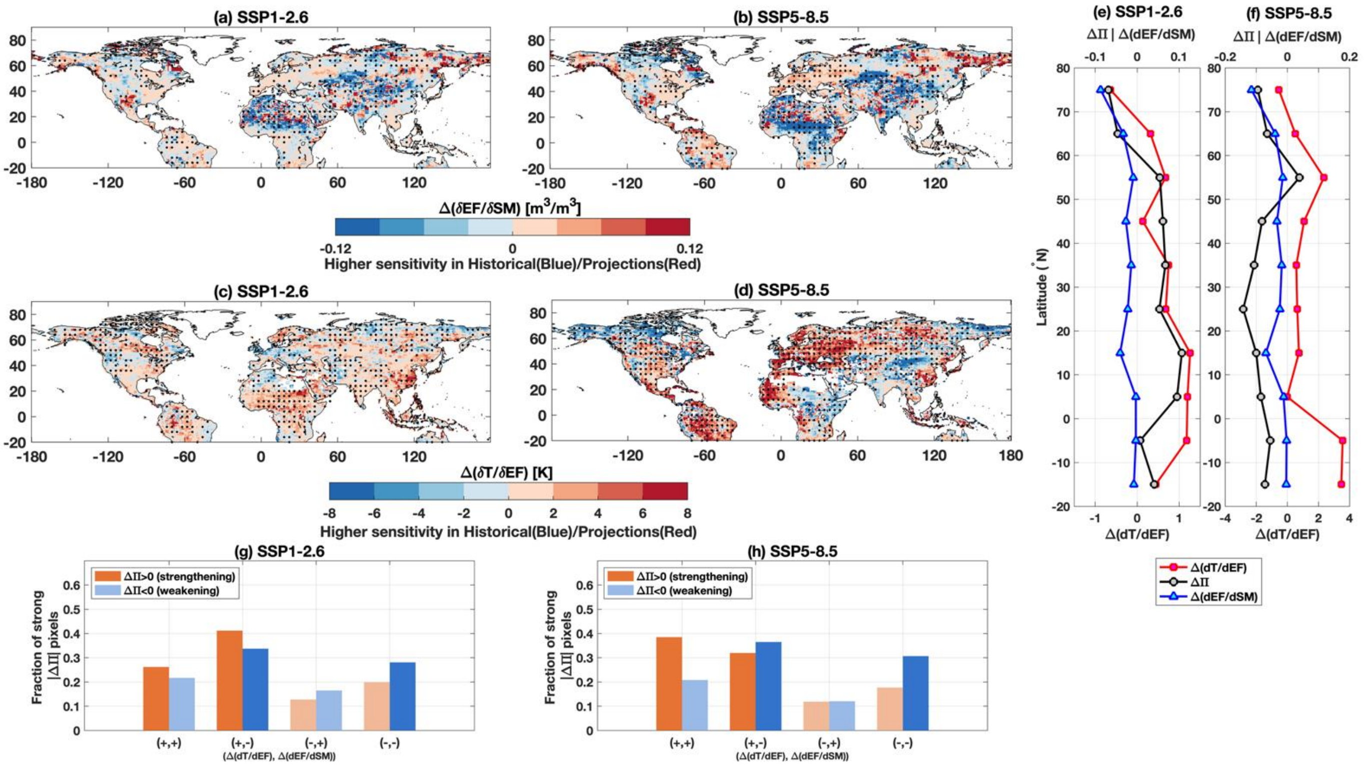

Fig. 2 | Future changes in the land and atmosphere segments of SM–T coupling. a, Changes in the land segments of the coupling, expressed as the EF sensitivity to SM changes (Δ(δEF/δSM), m^3m^{-3}), for the period 2066–2100 relative to 1981–2014 under SSP1-2.6. **b**, same as **a**, but for SSP5-8.5. **c**, Changes in the atmospheric segments of the coupling, expressed as T sensitivity to EF changes (Δ(δT/δEF), K), for the period 2066–2100 relative to 1981–2014 under SSP1-2.6. **d**, same as **c**, but for SSP5-8.5. –(δT/δEF) is used here for visual consistency. Dots indicate regions where at least 8 of the models agree on the sign of the change in the segments. All the segment computations are done at a 0.01 significance level based on a z-test. Latitude-binned median changes (10° bins) in ΔΠ, Δ(δEF/δSM), and Δ(δT/δEF), computed within the top 60% of historical |Π| for **e**, SSP1-2.6 and **f**, SSP5-8.5. Quadrant analysis of co-occurring segment changes for the strongest 60% of |ΔΠ| pixels for **g**, SSP1-2.6 and **h**, SSP5-8.5. Bars show the fraction of strengthening (ΔΠ > 0; orange) and weakening (ΔΠ < 0; blue) pixels falling into each combination of Δ(δT/δEF) and Δ(δEF/δSM) signs. Darker colored bars highlight the dominant co-occurrence patterns that most strongly characterise Π strengthening and weakening.

As discussed above, SM–T coupling is a nonlinear process that can be partitioned into land and atmospheric segments[18,43,49,55]. Both segments are expected to change under global warming, reflecting the influence of thermodynamic and dynamical perturbations on SM–T coupling. Here, we untangle these two segments of SM–T coupling to unravel the reasons for the changes in the coupling hotspot regions shown in Fig. 1. To do this, the land segment is evaluated by exploring the sensitivity of Evaporative Fraction (EF, the ratio between latent heat over net radiation) to changes in SM (i.e., δEF/δSM), and the atmospheric segment is assessed by exploring the sensitivity of air temperature to changes in EF (i.e., δT/δEF, expressed as negative for visual consistency)[43,49]. In nature, strong SM–T coupling is expected to occur where both sensitivities are favourable, capped by physical limitations in soil water availability and available energy[12].

Figure 2a–d show the projected changes in the two segments for SSP1-2.6 and SSP5-8.5, while Fig. 2e–g summarise their zonal behaviour and co-occurrence with ΔΠ. Importantly, the purpose of the segment-based analysis is not to reproduce the spatial pattern of ΔΠ, but to diagnose which physical segment—land or atmospheric—drives the changes in Π. Overall, the spatial patterns

suggest that warming modifies the two segments in distinct and regionally structured ways (Fig. 2a–d). Under SSP1-2.6 (Fig. 2a,c), the changes in both segments are generally small and spatially heterogeneous, which reflect modest adjustments in either land- or atmosphere-mediated sensitivity. By contrast, under SSP5-8.5 (Fig. 2b,d), the atmospheric segment shows stronger changes, with spatially coherent strengthening across large parts of the northern mid-latitudes (30–60°N). Here, $\Delta\delta T/\delta EF$ increases, demonstrating that a given perturbation in EF would produce a larger temperature response than in the future. Meanwhile, several semi-arid and subtropical regions exhibit reductions in $\delta EF/\delta SM$, suggesting that SM anomalies there become less effective in modulating EF than in the past, which indicates a weakening of land control. Together, these suggest that in emerging coupling hotspots, the dominant signal is enhanced atmospheric sensitivity, whereas in collapsing hotspots the dominant signal is reduced land control on EF which reflect a partial decoupling of EF from SM.

To diagnose which segment primarily underlies the changes in $\Delta\Pi$, we first aggregated all grid cells lying within the top 60% of historical Π, to represent the full historical SM–T transition belt. We then computed the median latitude-binned changes in $\Delta(\delta EF/\delta SM)$, $\Delta(\delta T/\delta EF)$, and $\Delta\Pi$ (Fig. 2e–f). Under SSP1-2.6, the three fields exhibit a moderate latitudinal structure, consistent with the muted changes in Π (Fig. 1b). This muted behaviour reflects the milder dynamic and thermodynamic changes under weak warming. By contrast, under SSP5-8.5 (Fig. 2f), mid- and high-latitude bands exhibit pronounced positive $\Delta(\delta T/\delta EF)$, while $\Delta(\delta EF/\delta SM)$ remains comparatively moderate. The zonal-mean $\Delta\Pi$ closely follows the trend of $\Delta(\delta T/\delta EF)$, indicating that the latitudinal shift and amplification of Π are primarily tied to changes in the atmospheric segment. Meanwhile, variations in $\delta EF/\delta SM$ contribute more selectively, particularly in regions where Π weakens or shifts poleward.

Finally, the co-variation between the two segments and Π strengthening ($\Delta\Pi>0$) or weakening ($\Delta\Pi<0$) is assessed by classifying the top 60% of $\Delta\Pi$ according to the sign of $\Delta(\delta T/\delta EF)$ and $\Delta(\delta EF/\delta SM)$ (Fig. 2g–h). Within each group, we compute the fractions of pixels falling into the four quadrants defined by the signs of $\Delta(\delta T/\delta EF)$ and $\Delta(\delta EF/\delta SM)$: (+,+), (+,−), (−,+), and (−,−). Under SSP1-2.6, the four sign combinations occur in broadly similar proportions for both Π strengthening and weakening. Strengthening pixels show a modest tendency toward $\Delta(\delta T/\delta EF)>0$,

whereas $\Delta(\delta EF/\delta SM)$ is split between positive and negative values, suggesting no dominant driving mechanism (Fig. 2g). Under SSP5-8.5, a clearer asymmetry emerges (Fig. 2h), where 70.4% of Π strengthening cases coincide with $\Delta(\delta T/\delta EF) > 0$, consistent with an enhanced sensitivity of near-surface temperature to evaporative variability. A substantial subset of these pixels also exhibits $\Delta(\delta EF/\delta SM) > 0$, suggesting that, in some regions, land and atmospheric segments reinforce each other. Conversely, weakening Π preferentially co-occurs with $\Delta(\delta EF/\delta SM) < 0$ (67.1%), demonstrating that a decline in the efficiency with which SM anomalies modulate EF is the dominant signature of hotspot weakening.

Overall, the contingency analysis (Fig.2g–h) highlights two complementary pathways of regime change. Hotspot strengthening occurs primarily where the atmosphere becomes more sensitive to EF, enhancing the temperature response to surface moisture variability. Hotspot weakening, by contrast, reflects a loss of land-surface control, where SM anomalies translate less efficiently into evaporative changes. These demonstrate the contrasting spatial evolution of SM–T coupling under weak and strong warming scenarios. Fig. 2 shows that the two segments play distinct roles, such that under strong warming, the emergence and poleward migration of coupling hotspots is primarily associated with increased atmospheric sensitivity to EF ($\Delta(\delta T/\delta EF)>0$), whereas the decay of historically strong hotspots is more often linked to a loss of SM control on EF ($\Delta(\delta EF/\delta SM)<0$).

Response to Reviewer

We thank the reviewer and editor for their continued engagement with the manuscript and for the constructive feedback provided in this round. We have carefully considered the concerns raised and have revised both analyses and text to improve clarity, interpretation, and presentation. Responses are provided in blue text and changes in the manuscript are written in *blue italics*.

Reviewer comment

Firstly I would like to thank the authors for following my suggestion to present the latitudinally averaged coupling metrics and contingency table in Fig2. I think it makes it much clearer why the section is so confusing.

Response

We thank the reviewer for this positive feedback. We agree that the revised presentation improves the clarity of the section, and we appreciate the suggestion to shift from soil-moisture binning to latitudinal/zonal analyses. This change has allowed us to more directly align Fig. 2 with the spatial patterns of $\Delta\Pi$ shown in Fig. 1 and with the conceptual framework in Fig. 4.

Reviewer comment

The latitudinal sections of del(dT/dET) and del(dEF/dSM) show quite similar changes for both SSP1-2.6 and SSP5-8.5, i.e. somewhat reduced del(dEF/dSM) and increased del(dT/dET). It seems that the main difference is that the magnitude of del(dT/dET) is different between the scenarios, but the del(dEF/dSM) looks identical between the two. From this it is very hard to understand the large reduction in Pi in the SSP5-8.5 run, in contrast to the increase in the other.

Response

We appreciate this important observation. We agree with the reviewer that, when viewed only in latitude-binned averages, $\Delta(\delta EF/\delta SM)$ appears similar between scenarios, while $\Delta(\delta T/\delta EF)$ differs mainly in magnitude. We further agree that this makes it difficult to infer the contrasting $\Delta\Pi$ responses from the segment plots alone.

However, we emphasize that the purpose of the segment analysis is *diagnostic rather than predictive*. The goal of Fig. 2 is not to reconstruct $\Delta\Pi$, but to diagnose which physical component of the coupling (land or atmospheric) dominates the changes under different scenarios. Π is a nonlinear metric that depends on multiple interacting processes (soil moisture

climatology, available energy, variability structure, and circulation changes). Therefore, one-to-one correspondence between segment changes and $\Delta\Pi$ is not expected.

To address the reviewer's concern, we have made several revisions to the manuscript to explicitly clarify this point and better explain why similar segment changes can still produce very different $\Delta\Pi$ outcomes. We have reframed the objectives leading to Fig. 2, added an explanation that the much larger magnitude of Δ(δT/δEF) under SSP5-8.5, together with regime shifts and circulation changes examined in Fig. 3, leads to a distinct Π response even when Δ(δEF/δSM) appears similar between scenarios, and clarified the nonlinearity of Π. The revised sections in the manuscript now read:

"As discussed above, SM–T coupling is inherently nonlinear and can be decomposed into a land segment and an atmospheric segment[18,43,49,55]. Both segments are expected to adjust under global warming as thermodynamic constraints (soil water availability and radiative energy) and dynamical conditions (boundary-layer and circulation influences) evolve. Here, we use this decomposition as a diagnostic framework to interpret the hotspot changes in Π shown in Fig. 1, rather than to provide a direct reconstruction of $\Delta\Pi$ from the two sensitivities. Specifically, we characterise the land segment by the sensitivity of evaporative fraction (EF; latent heat divided by net radiation) to soil moisture, δEF/δSM, and the atmospheric segment by the sensitivity of near-surface air temperature to EF variability, δT/δEF (plotted with reversed sign for visual consistency)[43,49]. In physical terms, strong SM–T coupling is most likely where (i) EF responds appreciably to soil moisture anomalies and (ii) temperature responds appreciably to EF fluctuations, subject to constraints imposed by soil water supply and available energy[12].

Figure 2a–d present the projected changes in the two segment sensitivities under SSP1-2.6 and SSP5-8.5, while Fig. 2e–h summarise their zonal behaviour and co-occurrence with $\Delta\Pi$. Importantly, the purpose of this analysis is to diagnose dominant physical sensitivities, not to reproduce the spatial pattern of $\Delta\Pi$ itself. The maps reveal that global warming modifies the land and atmospheric segments in distinct and regionally structured ways (Fig. 2a–d). Under SSP1-2.6 (Fig. 2a,c), changes in both segments are generally weak and spatially heterogeneous, indicating only modest adjustments in the sensitivities governing SM–T interactions. The atmospheric segment shows limited coherent change, and variations in the land segment are largely mixed in sign, consistent with the muted and spatially patchy $\Delta\Pi$ responses in this scenario. Under SSP5-8.5 (Fig. 2b,d), however, a clearer pattern emerges. The atmospheric segment exhibits widespread strengthening, particularly across the northern mid-latitudes (30–60°N), where Δ(δT/δEF) becomes more positive. This indicates that, in these regions, a given perturbation in EF would elicit a larger temperature response in the future climate, implying that temperature becomes more tightly controlled by local surface fluxes and less moderated by non-local atmospheric processes. In contrast, several semi-arid and subtropical regions display reductions in δEF/δSM, implying that

soil moisture anomalies become less effective at modulating EF than in the historical period—an indication of weakening land surface control. Taken together, these patterns suggest two contrasting tendencies: in regions where coupling hotspots emerge or intensify, the dominant signature is enhanced atmospheric sensitivity to EF variability, whereas in regions where hotspots weaken, the prevailing signal is a decline in the efficiency with which SM anomalies translate into evaporative changes, reflecting a lower coupling of EF from SM."

(more in the responses below)

Reviewer comment

If only the segment analysis, not the Pi metric, were presented, I would interpret this as showing an increase in SM-T in both scenarios, driven by an increase in the del(dT/dET) term.

However, this is not what the authors conclude

Response

We agree with the reviewer that interpreting the Jacobians alone could lead to that impression. For this reason, we explicitly avoid relying on them in isolation. The contingency analysis in Fig. 2g–h indicate that segment changes do not map one-to-one onto $\Delta\Pi$ at the grid scale. This is expected, because Π integrates multiple nonlinear processes beyond the two sensitivities.

To avoid any unintended interpretation that segment changes uniquely determine $\Delta\Pi$, we have revised the text to emphasize that Fig. 2 identifies aggregate tendencies, not direct causation. The updated text now states:

"*To diagnose which segment primarily underlies changes in Π, we focused on grid cells within the top 60% of historical $|\Pi|$, representing the principal historical SM–T transition belt. Within this subset, we computed median latitude-binned changes in $\Delta(\delta EF/\delta SM)$, $\Delta(\delta T/\delta EF)$, and $\Delta\Pi$ (Fig. 2e–f). This aggregation indicates a large-scale perspective on how the dominant segment sensitivities evolve across latitude, while reducing local noise and model-specific variability. Under SSP1-2.6 (Fig. 2e), all three fields display only weak and irregular latitudinal structure, consistent with the generally modest changes in Π shown in Fig. 1b. The limited coherence of both $\Delta(\delta T/\delta EF)$ and $\Delta(\delta EF/\delta SM)$ reflects the relatively small thermodynamic and dynamical perturbations under weak warming, consistent with coupling responses remaining spatially heterogeneous. Under SSP5-8.5 (Fig. 2f), a much clearer signal emerges. Mid- and high-latitude bands exhibit pronounced positive $\Delta(\delta T/\delta EF)$, indicating a systematic increase in atmospheric sensitivity to evaporative variability. In contrast, $\Delta(\delta EF/\delta SM)$ remains comparatively moderate and mixed in sign, with only localized reductions in land segment sensitivity. The latitude-binned $\Delta\Pi$ closely tracks the pattern of $\Delta(\delta T/\delta EF)$, consistent with the large-scale amplification and poleward expansion of coupling hotspots being primarily associated with enhanced atmospheric responsiveness, rather than with*

uniform changes in land surface control. At the same time, deviations between ΔΠ and Δ(δT/δEF) in specific latitude bands highlight that segment changes alone do not uniquely determine ΔΠ, consistent with the nonlinear and regime-dependent nature of the Π metric."

Reviewer comment

the contingency table makes it very clear that there is not a good correspondence between delPi, del(dT/dET) and del(dEF/dSM) at the gridpoint level. For example, for a given gridpoint in SSP-2.6, where either both del(dT/dET) and del(dEF/dSM) are increasing (+,+) or both are decreasing (-,-) the change in Pi is positive or negative for about 50% of grid points respectively, suggesting that variations in these two properties do not explain much of the variability in Pi. As expected I think this makes the clean explanation of the regional divergence hard to justify based on this analysis.

Response

We agree that the contingency analysis reveals imperfect grid-scale correspondence, and we now explicitly acknowledge this in the manuscript. However, the analysis also reveals robust scenario-dependent asymmetries that are highly informative.

Under SSP5-8.5:

- 70.4% of Π strengthening pixels coincide with Δ(δT/δEF) > 0,
- 67.1% of Π weakening pixels coincide with Δ(δEF/δSM) < 0.

These directional tendencies indicate that, although individual grid cells behave nonlinearly, the two segments play distinct and complementary roles when considered in aggregate. We have therefore reframed our conclusions to emphasize tendencies rather than deterministic explanations.

The revised Results section now reads:

"Finally, the co-variation between segment changes and Π strengthening (ΔΠ>0) or weakening (ΔΠ<0) is examined using a contingency-style analysis applied to the top 60% of |ΔΠ| pixels, representing locations with the strongest coupling changes (Fig. 2g–h). For each scenario, pixels are classified according to the signs of Δ(δT/δEF) and Δ(δEF/δSM), yielding four possible sign combinations: (+,+), (+,−), (−,+) and (−,−). Within the strengthening and weakening subsets, we compute the fraction of pixels falling into each quadrant. Under SSP1-2.6 (Fig. 2g), the four sign combinations occur in broadly comparable proportions for both Π strengthening and weakening. Although strengthening pixels exhibit a modest preference for Δ(δT/δEF)>0, Δ(δEF/δSM) is nearly evenly split between positive and negative values. This lack of a dominant quadrant indicates that,

under weak warming, no single segment consistently governs coupling changes, consistent with the muted and spatially heterogeneous $\Delta\Pi$ patterns. Under SSP5-8.5 (Fig. 2h), a markedly different structure emerges. Among Π strengthening pixels, 70.4% coincide with $\Delta(\delta T/\delta EF)>0$, indicating that coupling intensification preferentially occurs where the atmosphere becomes more sensitive to evaporative variability. A substantial subset of these pixels also exhibits $\Delta(\delta EF/\delta SM)>0$, suggesting that in some regions land and atmospheric segments act in concert. In contrast, Π weakening pixels show a strong preference for $\Delta(\delta EF/\delta SM)<0$ (67.1%), demonstrating that a reduction in the efficiency with which soil moisture anomalies modulate EF is the dominant signature of hotspot weakening. Importantly, these associations emerge at the aggregate level despite imperfect grid-scale correspondence, highlighting that Π responds nonlinearly to segment changes but nonetheless exhibits clear, scenario-dependent tendencies."

Reviewer comment

"It does make it quite hard to conclude much from this section since the metrics show quite different things."

Response

We respectfully disagree that little can be concluded. Rather, the analysis shows that:

1. Segment changes alone cannot fully explain $\Delta\Pi$,
2. The atmospheric segment undergoes much larger perturbations under strong warming,
3. Strengthening and weakening of Π are governed by different physical levers,
4. Dynamical processes (Fig. 3) play a key mediating role.

To make this logic clearer, we have:

- Rewritten the Fig. 2 Results section to explicitly state the diagnostic purpose of the analysis,
- Added text explaining why similar $\Delta(\delta EF/\delta SM)$ between scenarios does not imply similar $\Delta\Pi$,
- Clarified that Fig. 3 provides the dynamical explanation for the divergent responses,
- Harmonized the x-axes of all panels as requested by the editor.

Furthermore, following the editor's guidance, we have also:

- Used consistent x-axis ranges for both scenarios in Fig. 2,

- Updated the contingency analysis to focus on the top 60% of $|\Delta\Pi|$,
- Revised the figure caption and text to avoid implying a direct deterministic relationship between segment changes and Π.

The revised manuscript now (i) defines the diagnostic purpose of the segment analysis, (ii) presents harmonized latitude-binned comparisons, (iii) introduces a formal contingency framework to quantify co-occurrence patterns, and (iv) links these thermodynamic tendencies with dynamical mechanisms in Fig. 3. Furthermore, the residual discrepancies between segment changes and $\Delta\Pi$ are addressed through the new Fig. 3 analysis, which demonstrates that circulation-driven shifts in ω_{500} and P–E modulate how segment sensitivities translate into actual coupling responses. We believe these revisions directly address the reviewer's concerns and provide a balanced and transparent interpretation.

Once again, we are grateful to the reviewer for prompting these clarifications. The revisions have helped us present Fig. 2 in a more transparent and balanced way. We believe the manuscript now clearly communicates that:

- Segment sensitivities provide thermodynamic context,
- Π responds nonlinearly to these sensitivities,
- Dynamical processes (Fig. 3) primarily determine how these changes translate into hotspot shifts.

We hope that these revisions satisfactorily address the reviewer's concerns and improve the clarity and rigor of the study.

Editor's comment

You will see that, the reviewer still raises some important concerns, therefore, you will need to elaborate on the discrepancies highlighted by the reviewer and clarify how your conclusions account for the variation in del(Pi) between SSP1-2.6 and SSP5-8.5, while maintaining a constant del(dEF/dSM). Ideally, you should use the same x-axis values for both scenarios, as this would make it easier to compare the differences in the metrics.

Response

Thank you for the helpful suggestion. Please find the clarification of our conclusions as addressed in the responses above. The suggestion with regards to Figure 2 has also been implemented. Please see the revised figure below:

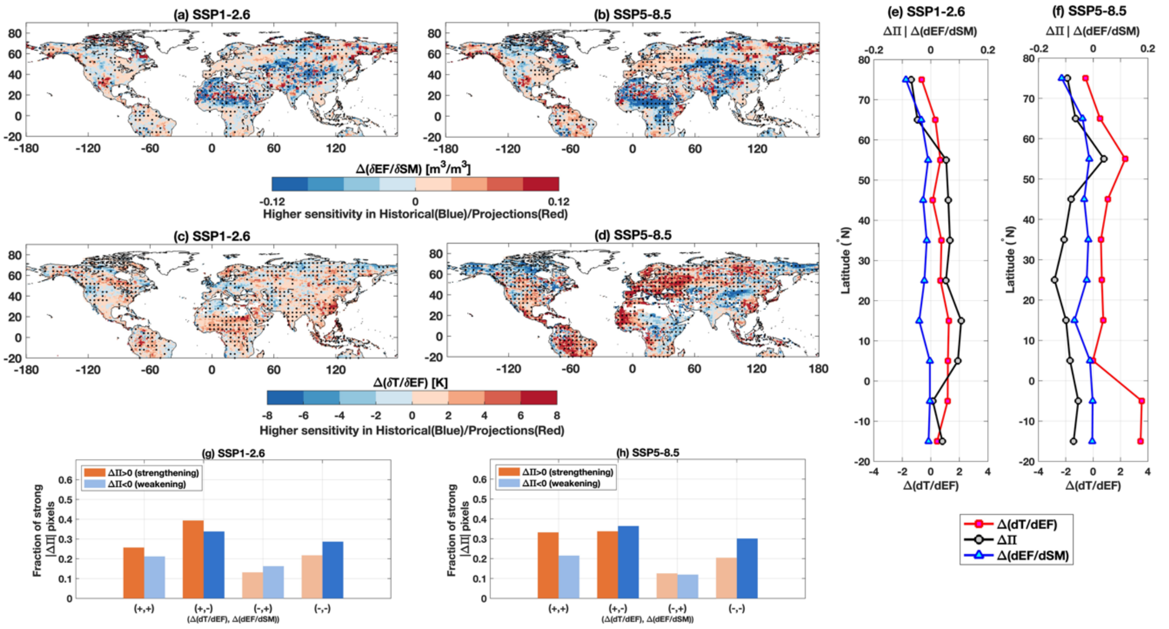

Fig. 2 | Future changes in the land and atmosphere segments of SM–T coupling. a, Changes in the land segment of the coupling, expressed as the EF sensitivity to SM changes (Δ(δEF/δSM), m^3m^{-3}), for the period 2066–2100 relative to 1981–2014 under SSP1-2.6. **b**, same as **a**, but for SSP5-8.5. **c**, Changes in the atmospheric segment of the coupling, expressed as T sensitivity to EF changes (Δ(δT/δEF), K), for the period 2066–2100 relative to 1981–2014 under SSP1-2.6. **d**, same as **c**, but for SSP5-8.5. –(δT/δEF) is used here for visual consistency. Dots indicate regions where at least 8 of the models agree on the sign of the change in the segments. All the segment computations are done at a 0.01 significance level based on a z-test. Latitude-binned median changes (10° bins) in ΔΠ, Δ(δEF/δSM), and

$\Delta(\delta T/\delta EF)$, computed within the top 60% of historical $|\Pi|$ for **e**, SSP1-2.6 and **f**, SSP5-8.5. Quadrant analysis of co-occurring segment changes for the strongest 60% of $|\Delta\Pi|$ pixels for **g**, SSP1-2.6 and **h**, SSP5-8.5. Bars show the fraction of strengthening ($\Delta\Pi > 0$; orange) and weakening ($\Delta\Pi < 0$; blue) pixels falling into each combination of $\Delta(\delta T/\delta EF)$ and $\Delta(\delta EF/\delta SM)$ signs. Darker colored bars highlight the dominant co-occurrence patterns that most strongly characterize Π strengthening and weakening.

I would like to thank the authors for their work to improve the manuscript. However, I still believe some additional changes are required before the manuscript is ready for publication. I found the section on land and atmosphere segments difficult to read, and the metric chosen in conflict with those in the supplementary material (Fig S5). I also found the conclusion that there is a fundamental difference in the response to low SSP and high SSP not well justified. This is because although the response in the Pi metric in Fig 1 shows a difference between the SSPs, the other metrics shown in Figs S4 and S5 show much more similarity between SSPs, with the SSP126 just showing a weaker response than SSP585 with a broadly similar pattern suggesting the Pi metric is really an outlier here.

It occurred to me when performing this second review that the choice of metrics is not really discussed. Some metrics are shown in the main paper, others shown in the supplement but without any justification for the choices of what metric is used where. The paper by Lorenz et al. (2015) might be useful to reference here as it includes a discussion about the impact the choice of metric has on estimates on the change in SM-atmosphere coupling.

Overall comments

The paper looks at the impact of climate change on land-atmosphere coupling, which is a major source of uncertainty in 2m-temperature, projections, and the influence of SSP on these changes. It is found that high and low emissions pathways have quite different impacts on the change in SM-T coupling with coupling tending to be enhanced under low emissions pathways, such as SSP-1-2.6. In contrast, a more complex response is seen under high emissions pathways, such as SSP-5-8.5, where coupling is reduced in existing mid-latitude and sub-tropical hotspots due to a reduction in SM (due to an expanded Hadley circulation and higher precip), but enhanced at higher latitude as a result of soil-moisture drying there.

Overall, I found the results in the paper interesting, the conclusions plausible and a of significance. However, I found there was a general lack of statistical testing, making it hard to completely assess the robustness of the findings. For example, none of the maps of changes under the various emissions pathways give any measure of agreement in the sign of the change or it's statistical significance. As a result, one is left wondering if all the models have the same behaviour, or just one or two. There are also some missing methodological details that would make it hard to reproduce form this manuscript (but which can be easily corrected).

Response: Thank you for these comments. We have updated Fig. 1 to include model agreement for the signs of the changes. Please see the updated figure below. Additionally, we have added included necessary details to improve reproducibility. Please see the details in the responses below.

The paper would also benefit from more careful editorial work to improve the text and make it easier to follow. I provide some examples but don't make a comprehensive list.

Response: Thank you. The authors have gone through to improve the textual integrity of the manuscript.

I wasn't very keen on using the term "bifurcation" to describe different regional responses to climate change or to differences between emissions pathways. It sounds grander than "regional differences" or "differences between emission pathways" but the term has quite a specific definition in dynamical systems theory, and I found it a bit distracting. This terminology may also be physically wrong, for example, it may be that under SSP-5-8.5 the models undergo a strengthening in the existing hotspots in the early 21st century, before this weakening of existing hotspots and northward expansion (seen at the end of century) due to dynamical changes takes over. This would make it more of a transitional state than a bifurcation. This could be checked by looking at the earlier decades of the SSP-5-8.5 projections and would provide an indication of what the early warnings signs are of such a transition.

Response: We are grateful to the reviewer for their insightful assessment. Indeed, we discussed at length about the terminology. Our eventual decision to use 'bifurcation 'highlights two main points: (1) the diverging transitions of the regions to either strengthen or weaken even under the same scenario, and (2) the seemingly contrasting transitions regions undergo due to different warming scenarios, especially in the low- and high-end emission scenarios used here. We agree with the reviewer that the title might need a change. After a discussion, the authors have decided to change it to

"**Divergent regional responses of soil moisture-air temperature coupling under future climate scenarios**".

We believe this is a better reflection of the findings and content of the study.

The response here missed my second point about the possiblility that the enhanced mid-latitude coupling being a transition state.

Specific comments

Fig 1: I think it would be useful to indicate some measure of agreement in the ensemble mean changes in Π, and also include a contour of Π in the historical runs in Fig1d and e so that it is easy to see how the changes in Π correspond to the present-day hotspots. Also for Fig S2.

Response: Thank you. We ackowledge the reviewer's suggestion. We note that in panels a-c from Figure 1 already used stippling to indicate agreement, as indicated by the figure caption: "Dots indicate hotspot regions where at least 8 of the models agree". We have not added a contour as it was obscured by the stippling to show model agreement. We believe it is more important to highlight the model agreement to opted for stippling instead of the contour. We also agree that a similar scheme should also be included in panel (d) and (e) as well as in Fig. S2. Accordingly new versions of Fig.1 and Fig. S2 were included as below:

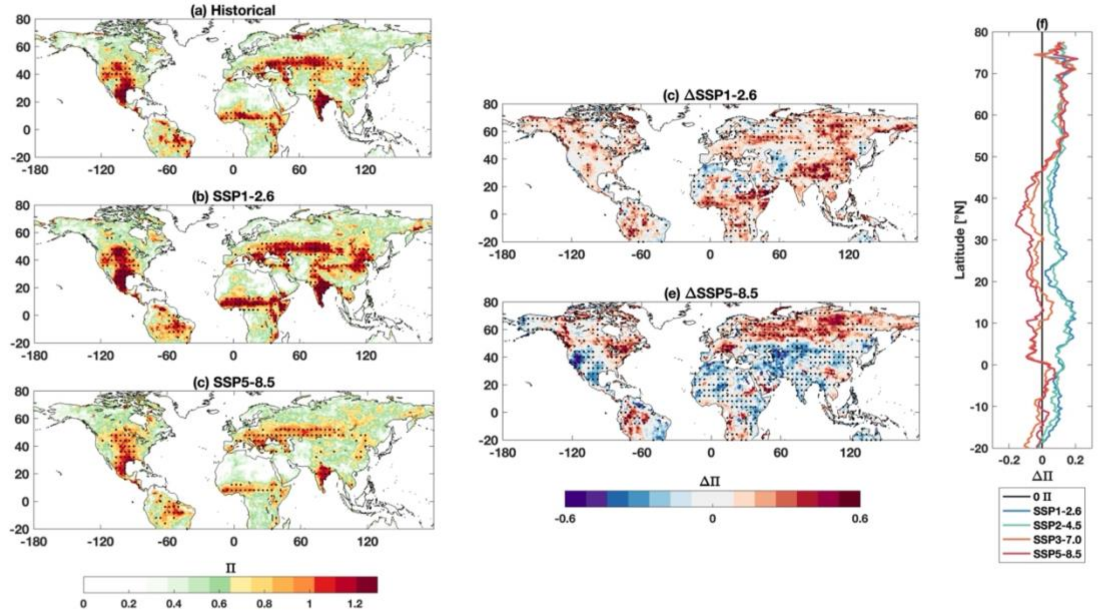

Fig. 1 | SM–T coupling and projected changes in boreal summer. a, SM–T coupling (Π) based on the CMIP6 ensemble mean of the historical period (1981–2014). **b-c**, Same but for future projections (2066–2100) under **b**, SSP1-2.6 and **c**, SSP5-8.5. Dots indicate hotspot regions where at least 8 of the models agree on Π > 0.75. **d–e**, Change in SM–T coupling (i.e., $\Delta\Pi$) during 2066–2100 relative to 1981–2014 under **d**, SSP1-2.6 and **e**, SSP5-8.5. Dots indicate regions where at least 8 of the models agree on the sign of the change in Π. **f**, Mean latitudinal profile of $\Delta\Pi$ for the future (SSP1-2.6, SSP2-4.5, SSP3-7.0 and SSP5-8.5) compared to the historical period. The

correlations behind the Π metric are computed at a 0.01 significance level, and non-significant correlations are shown in white.

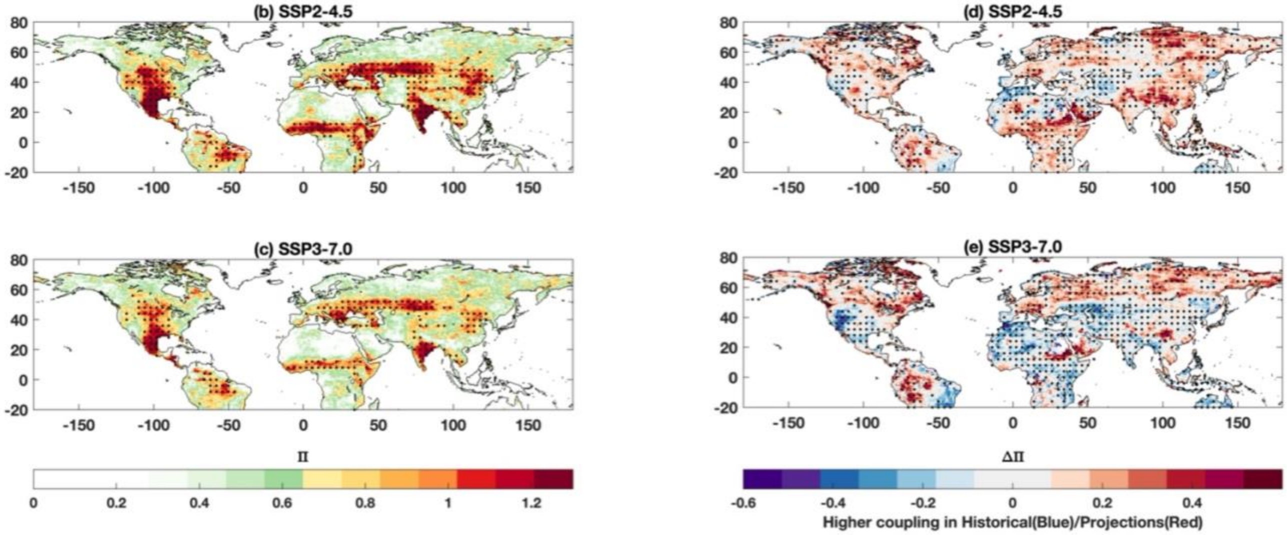

Fig. S2. **The SM–T coupling and projected changes in boreal summer. a**, Expansion (green) and contraction (red) of the historical SM–T coupling hotspots (HS) based on SSP1-2.6 and SSP5-8.5. Common HS refers to hotspots present in both the historical and future (SSP1-2.6 and SSP5-8.5) periods. Ensemble mean of Π from CMIP6 projections (2015–2100) under **b**, SSP2-4.5 and **c**, SSP3-7.0. The dotted regions (i.e., the coupling 'hotspots') in Fig. 1a-e indicate where more than 80% of the models agree on Π >0.7. Change in Π (i.e., $\Delta\Pi$) during the period 2015–2100 under **d**, SSP2-4.5 and **e**, SSP3-7.0 relative to 1981–2014. Dots indicate regions where at least 9 of the models agree on the sign of the change in Π.

Suggest stating the percentage of models agreeing on the sign of the response as a percentage as well, so the casual reader doesn't have to search for the number of models used.

L102-110: I found this paragraph a somewhat uncritical appraisal of the CMIP6 ensemble's ability to capture the strength of land-atmosphere coupling. It would be helpful if the colour bar in Fig 1a-c matched with Fig S1, but also useful to include a bias map for the metric in Fig S1, so which highlights the differences. By eye, there seems to be a general tendency to overestimate the coupling strength, compared to Fig S1a as also seen in Hsu and Dirmeyer (2022) for CMIP5 and also Day et al. (2025), for seasonal models. These studies both show that climate models tending to be in a soil-limited evaporation regime over a larger area of the mid-latitudes than the real world. Not having the historical runs in

the correct SM-E regime will have some important implications for the ability to predict transitions from one regime to the other, so this caveat needs to be more critically discussed as a limitation of the study.

Response: Thank you for the comment. We consistently inspected our multi-model model results to verify that in each case, at least, 80% of the models were in agreement with respect to mean values and signs (e.g. the revised version of Fig.1 and Fig.S1). Furthermore, we have added some text to highlight these limitations in the Data and Methods Section, under SM–T coupling. It now reads:

While we consistently inspect agreement in the CMIP6 multi-model analyses (at least, 80% of the models agree), CMIP6 ensembles are known to over-represent soil moisture constraints on evaporation and overestimate, which has implications for the SM–T coupling computations over mid-latitudes[37]. While our historical evaluation against observations lends confidence, our findings should be interpreted as model-conditioned tendencies rather than absolute magnitudes, acknowledging that consensus can mask the structural biases shared across models.

References

Hsu, H. & Dirmeyer, P. A. Deconstructing the Soil Moisture–Latent Heat Flux Relationship: The Range of Coupling Regimes Experienced and the Presence of Nonlinearity within the Sensitive Regime. *Journal of Hydrometeorology* **23**, 1041-1057 (2022). https://doi.org/https://doi.org/10.1175/JHM-D-21-0224.1

Fig S2: also need to make colourbars consistent with Fig's 1 and Fig S1.

Response: Thank you. We have set Fig S1a–b colour bars to be consistent with Fig 1. Please see the revised figure below.

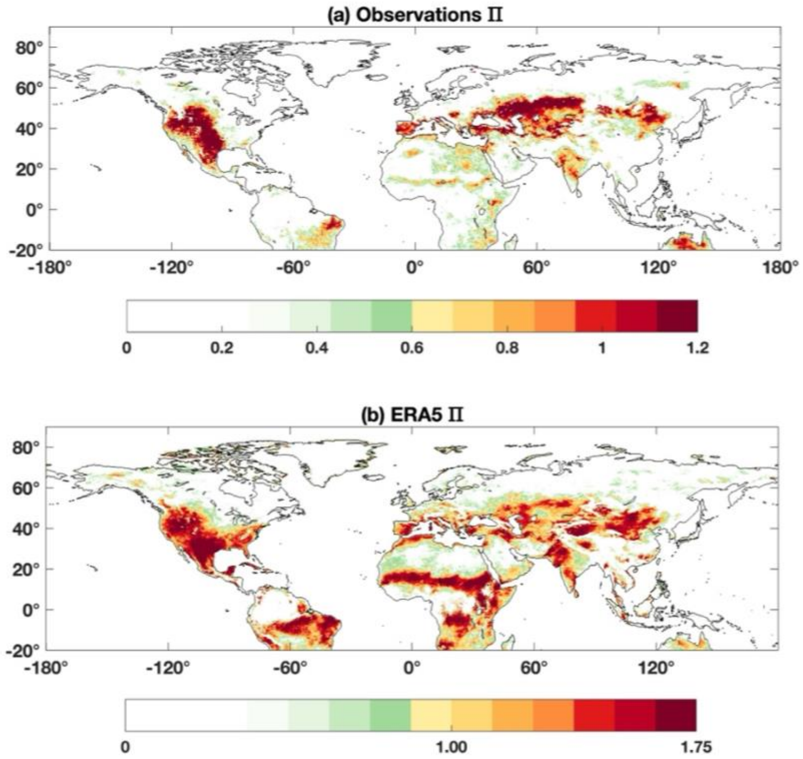

Fig. S1. **Observation- and reanalysis-based boreal summer SM–T coupling.** The SM–T coupling in boreal summer (June, July, August) based on the Π diagnostic calculated using (a) T from CRU and E from GLEAM, and (b) ERA5 reanalysis outputs during the historical period (1981–2014).

Please also make the fig S1b colourbar consistent.

L111: “weakening” to “weakened”

Response: Done.

L153: This is very similar to the approach of Dirmeyer (2011) and Dirmeyer et al. (2014) who described a terrestrial and atmospheric leg to the coupling. Suggest citing these papers here and framing in terms of terrestrial-leg and atmospheric-leg.

Response: Indeed, the concept is similar, and while we specifically followed Schwingshackl et al. (2017, 2018) in this case, we agree to the importance of also citing Dirmeyer (2011,2014) where these ideas were earlier introduced. We also decided to use ‘segments’ in keeping with Dirmeyer (2011). We have added the references, which now reads:

As mentioned above, SM–T coupling is a nonlinear process that can be partitioned into land and atmospheric segments[18,43,48,53].

References

Schwingshackl, C., Hirschi, M. & Seneviratne, S. I. A theoretical approach to assess soil moisture–climate coupling across CMIP5 and GLACE-CMIP5 experiments. *Earth Syst. Dynam.* **9**, 1217-1234 (2018). https://doi.org/10.5194/esd-9-1217-2018

Schwingshackl, C., Hirschi, M. & Seneviratne, S. I. Quantifying Spatiotemporal Variations of Soil Moisture Control on Surface Energy Balance and Near-Surface Air Temperature. *Journal of Climate* **30**, 7105-7124 (2017). https://doi.org/https://doi.org/10.1175/JCLI-D-16-0727.1

Dirmeyer, P. A. The terrestrial segment of soil moisture–climate coupling. *Geophysical Research Letters* **38** (2011). https://doi.org/https://doi.org/10.1029/2011GL048268

L161: Rather than saying "negated" just put a minus in front of the expression.

Response: Done.

Also change associated figure caption

L139-198: I found this section quite hard to follow. It also seems that the metrics used to measure the coupling strength in this section seem not to capture the same changes seen in the previous section with the Π metric (figs 1d & e vs. Fig 2c-f). The section seems to be framed in terms of understanding changes in Π with the two derivatives, but they measure quite different things. As a result, I think this section confuses things rather than explaining them. Then Fig S4 shows something quite similar to the change in Π.

Response: Thank you for this comment. We agree with the reviewer this part could be clearer. The goal here is to unveil whether Π changes come from the and/or atmospheric legs, while using other independent metrics to corroborate the pattern changes in Π. We did not expect a 1:1 alignment, instead we anticipate some explainability between the two, which we have demonstrated both through our results and the texts. Given that this is a nonlinear process, which Pi and the CSM express in linear forms, it makes sense that we do not have a exact representation. Eventually, what we find is that both the partitioned results and the results of the changes in dynamics provide a wholesome explainability (with their individual limitations) to explain the changes in we find in Π. To make our arguments of the partitions stronger, we have added the partitions of the CSM method as well to Fig. S5 which largely validates both the changes in the hotspots and to segments of the coupling. The text in this section has also been updated.

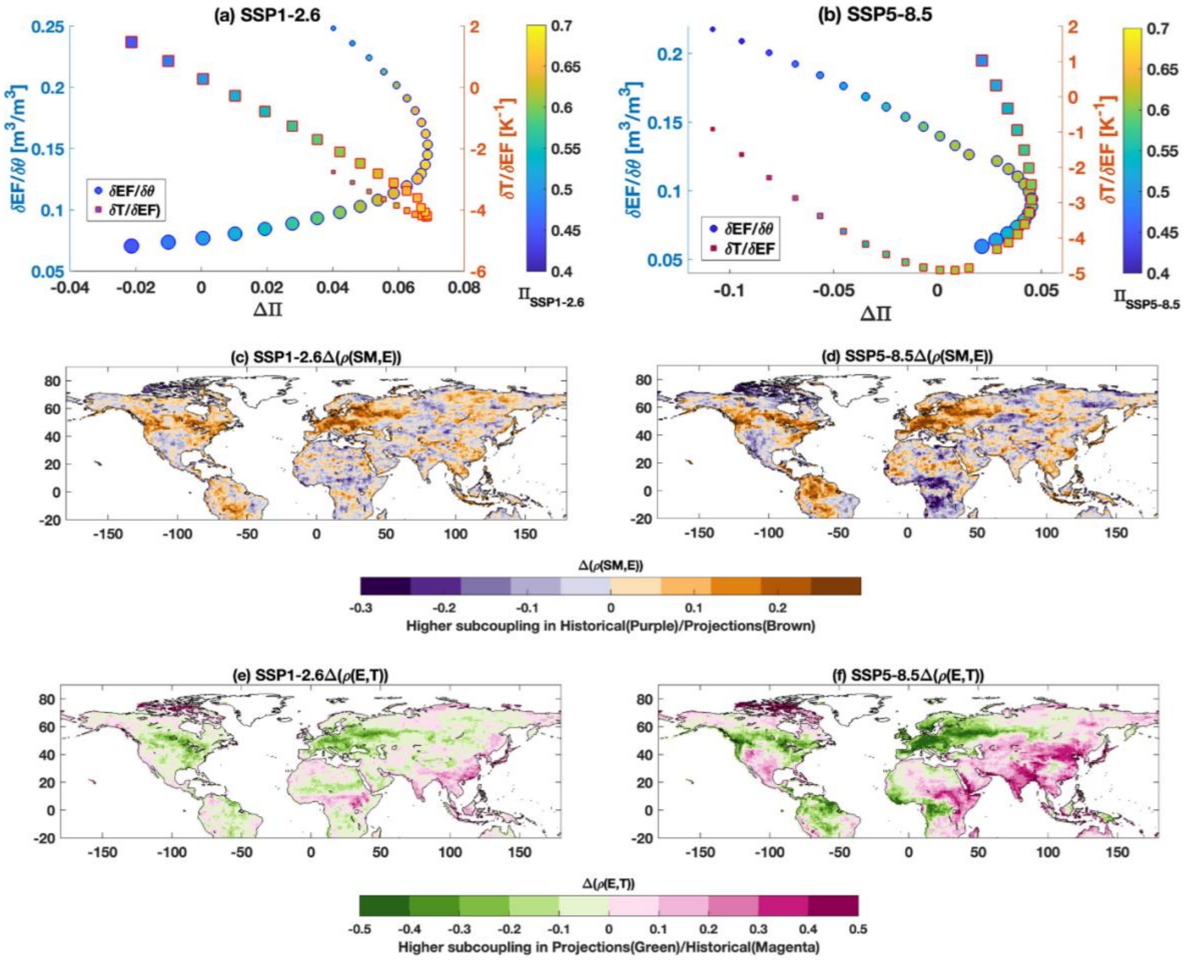

Fig. S5 a, Coupling Partitioning Analysis. Land (left y-axis, circles) and atmospheric (right y-axis, squares) legs of the coupling under SSP1-2.6 binned across historical soil moisture climatology, plotted as a function of change in future SM–T coupling ($\Delta\Pi$). Color shades represent absolute Π. **b**, same as **a**, but SSP5-8.5. The terrestrial leg changes of CSM are shown in **c–d**, and the atmospheric leg is shown in **e–f** for the two scenarios.

Lines 198-199 also read:

Furthermore, the segment-based analysis of CSM (Fig. S5c–f) also align with these findings to explain changes in the SM–T coupling.

Unfortunately, I still found this section very difficult to follow. I understand that the changes seen in the linear segment analysis in Fig 2 will not match the changes in Π (Fig 1) perfectly, but they don't look to me like they match what is seen in Fig 1 at all. In contrast the change in correlation metrics shown in Fig S5d & f look to capture a similar pattern to Fig 1e quite clearly, i.e. the dipoles in Π North America and Eurasia. As a result, I think the section on

the land and atmosphere, which is supposed to elucidate the mechanisms, actually confuses things greatly. For example, in fig 1e there is a clear latitudinal dependence to the change in coupling strength, with the northern mid-latitudes reducing and the higher latitudes increasing. I can't see this basic pattern in Fig 2. It is also not clear to me why the changes in this metric are expressed as a function of the SM in the historical run. It is not clear to me what insight this gives compared to plotting as a function of latitude, which would make the comparison with Fig 1 easier to see.

After having a closer look at Fig S5, I am also slightly concerned that the papers main conclusion (RE divergent responses to different scenarios) may be quite metric dependent. This is because the correlation metrics used in Fig S5 show much more similarity between the response under the SSP1-2.6 scenario and the SSP5-8.5 scenario than in Fig 1. The change in both metrics have a dipole over Eurasia and north America.

Lorenz, R., A. J. Pitman, A. L. Hirsch, and J. Srbinovsky, 2015: Intraseasonal versus Interannual Measures of Land–Atmosphere Coupling Strength in a Global Climate Model: GLACE-1 versus GLACE-CMIP5 Experiments in ACCESS1.3b. *Journal of Hydrometeorology*, **16**, 2276–2295, https://doi.org/10.1175/JHM-D-14-0206.1.
